# Midbrain PAG Astrocytes Modulate Mouse Defensive and Panic‐Like Behaviors

**DOI:** 10.1002/advs.202506062

**Published:** 2026-02-04

**Authors:** Ellane Barcelon, Kyungchul Noh, Minkyu Hwang, Yoon‐Jung Kim, Unjin Lee, Yeon Joo Ryu, Je‐Kyung Ryu, Sang Beom Jun, Se‐Young Choi, Woo‐Hyun Cho, Sung Joong Lee

**Affiliations:** ^1^ Department of Physiology and Neuroscience Dental Research Institute Seoul National University School of Dentistry Seoul Republic of Korea; ^2^ Spatial Navigation and Memory Unit National Institute of Neurological Disorders and Stroke National Institutes of Health Bethesda Maryland USA; ^3^ Department of Pharmacology Ajou University School of Medicine Suwon Republic of Korea; ^4^ Department of Brain and Cognitive Neurosciences College of Natural Sciences Seoul National University Seoul Republic of Korea; ^5^ Interdisciplinary Program in Neuroscience College of Natural Sciences Seoul National University Seoul Republic of Korea; ^6^ Department of Physics and Astronomy Seoul National University Seoul Republic of Korea; ^7^ Department of Electronic and Electrical Engineering Ewha Womans University Seoul Republic of Korea; ^8^ Graduate Program in Smart Factory Ewha Womans University Seoul Republic of Korea; ^9^ Department of Brain and Cognitive Sciences Ewha Womans University Seoul Republic of Korea; ^10^ Institute for Neurological Therapeutics Rutgers‐Robert Wood Johnson Medical School Piscataway New Jersey USA

**Keywords:** PAG, astrocyte, defensive behavior, midbrain, panic behavior

## Abstract

Defensive behaviors against threatening situations are crucial for survival, and maladaptation of neural functions involved in defensive behavior may result in panic‐like behavior. Defensive behaviors must be optimized for animals to efficiently avoid danger and maximize their chance of survival. The midbrain periaqueductal gray (PAG) controls defensive behaviors. However, the substrate of dysregulated panic‐like defensive responses remains unknown. Using in vivo calcium imaging and recordings in mice, we found that PAG astrocytes are activated during threatening situations and trigger defensive behaviors. Using optogenetic astrocyte modulation and electrophysiological experiments, we provide evidence that PAG astrocyte activation and subsequent ATP release are required for optimal defensive behavior; aberrant activation of PAG astrocytes leads to maladaptive defensive behavior resembling panic‐like behavior. Our results suggest that PAG astrocytes are neurobiological substrates underlying defensive dysregulation and might be an important cue in panic‐related behaviors via increased calcium activity and ATP release.

## Graphical abstract

Astrocytes in the midbrain periaqueductal gray (PAG) dynamically encode threat intensity and shape defensive action selection in mice. Real‐time Ca^2^^+^ imaging reveals robust astrocytic activation during predator odor and CO_2_ exposure. Aberrant astrocytic Ca^2^^+^ overactivation disrupts goal‐directed escape, biases behavior toward freezing, and induces panic‐like responses through ATP release.

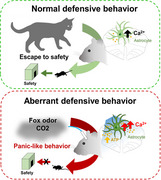

## 1 Introduction

Efficient and appropriate behaviors to avoid threats are fundamental for survival. Dysregulated threat responses can interfere with goal‐directed activities such as avoiding and reducing potential harm, safety learning, foraging, and other behaviors that are highly relevant to animal survival. Dysregulated fear and behavioral responses against threats, in the form of heightened fear or inefficient safety‐seeking, are the hallmark of pathological fear and anxiety disorders such as panic‐related behaviors [1, 2].

Previous studies showed that the midbrain periaqueductal gray (PAG) area is actively involved in detecting threats, judging their imminence, and generating various defensive responses, such as risk assessment, freezing, flight, and fight [3, 4, 5]. It is also a functional interface between the forebrain and brainstem that integrates emotion and cognition to compute certain tasks that animals must complete to respond to threatening stimuli in the environment [6, 7]. Studies have demonstrated increased BOLD activity in the midbrain PAG upon threat imminence and freezing in humans [8, 9, 10]. Moreover, the PAG is also implicated in panic‐like disorder [2, 11, 12, 13, 14]. It was reported that neurosurgical patients who underwent electrical stimulation of their PAG experienced panic‐related symptoms described as intense fear [15]. In rodents, electrical and chemical stimulation of the PAG elicited panic‐like behaviors and heightened defensive behaviors [13, 16]. Also, the use of panicogenic stimuli such as CO_2_ induced increased c‐Fos expression in the PAG and elicited jumping and freezing behaviors [17, 18]. Therefore, it was proposed that dysregulation of PAG‐mediated defensive behavior results in panic‐like disorders [14, 16]. However, the cellular substrate and regulatory mechanisms in PAG that orchestrate defensive behavior versus panic‐like behavior remain unclear.

Astrocytes, a component of tripartite synapses, play critical roles in brain function. Through their perisynaptic processes, astrocytes engage in bidirectional communication with pre‐ and post‐synaptic neurons to regulate synaptic transmission and thereby affect animal behavior [19, 20, 21, 22]. For example, astrocytes in the ventral tegmental area (VTA) orchestrate avoidance and approach behaviors by influencing extracellular glutamate levels via the astrocytic glutamate transporter GLT‐1 [23]. In addition, chemogenetic activation of astrocytes in the central amygdala reduced freezing behaviors in a cued fear conditioning experiment, decreasing the firing rate of central medial amygdala neurons by enhancing inhibitory synapses from the lateral central amygdala [24]. Studies have reported that astrocytes in the midbrain PAG are involved in facilitating descending nociceptive pathway and emotional motivation in neuropathic pain [25, 26].

Although these studies indicate that PAG astrocytes can modulate nociception and motivational processes, their potential role in ethologically relevant defensive and panic‐like behaviors has not been explored. Recent single‐cell and spatial transcriptomics data suggest a potential role for PAG astrocytes in a variety of survival and reproductive behaviors in mice, including aggression, parenting, mating behavior, and predator response [27]. In particular, no study has provided real‐time in vivo measurements of PAG astrocyte Ca^2^^+^ dynamics during threat exposure or established a causal link between astrocyte activity and panic‐like responses. Thus, it is critical to determine how astrocyte‐neuron interactions regulate PAG computations beyond nociception, particularly under panicogenic conditions.

We investigated whether PAG astrocyte activity is involved in defensive responses against threatening situations by measuring mouse PAG astrocyte calcium activity in vivo using time‐correlated single photon counting (TCSPC). We also investigated the in vivo role of PAG astrocyte activity through optogenetic and pharmacologic manipulation. Our findings demonstrate that PAG astrocytes increase Ca^2+^ activity in response to threatening situations, allowing mice to display appropriate defensive responses. We also show that aberrant PAG astrocyte activity disrupts appropriate and goal‐directed defensive responses against threatening situations in ways that resemble panic‐like behavior. We thus propose that aberrant Ca^2+^ activity and subsequent ATP release from PAG astrocytes might underlie maladaptive defensive responses and panic‐related behaviors.

## 2 Results

### 2.1 PAG Astrocyte Ca^2+^ Activity Increases in Threatening Situations

**FIGURE 1:**
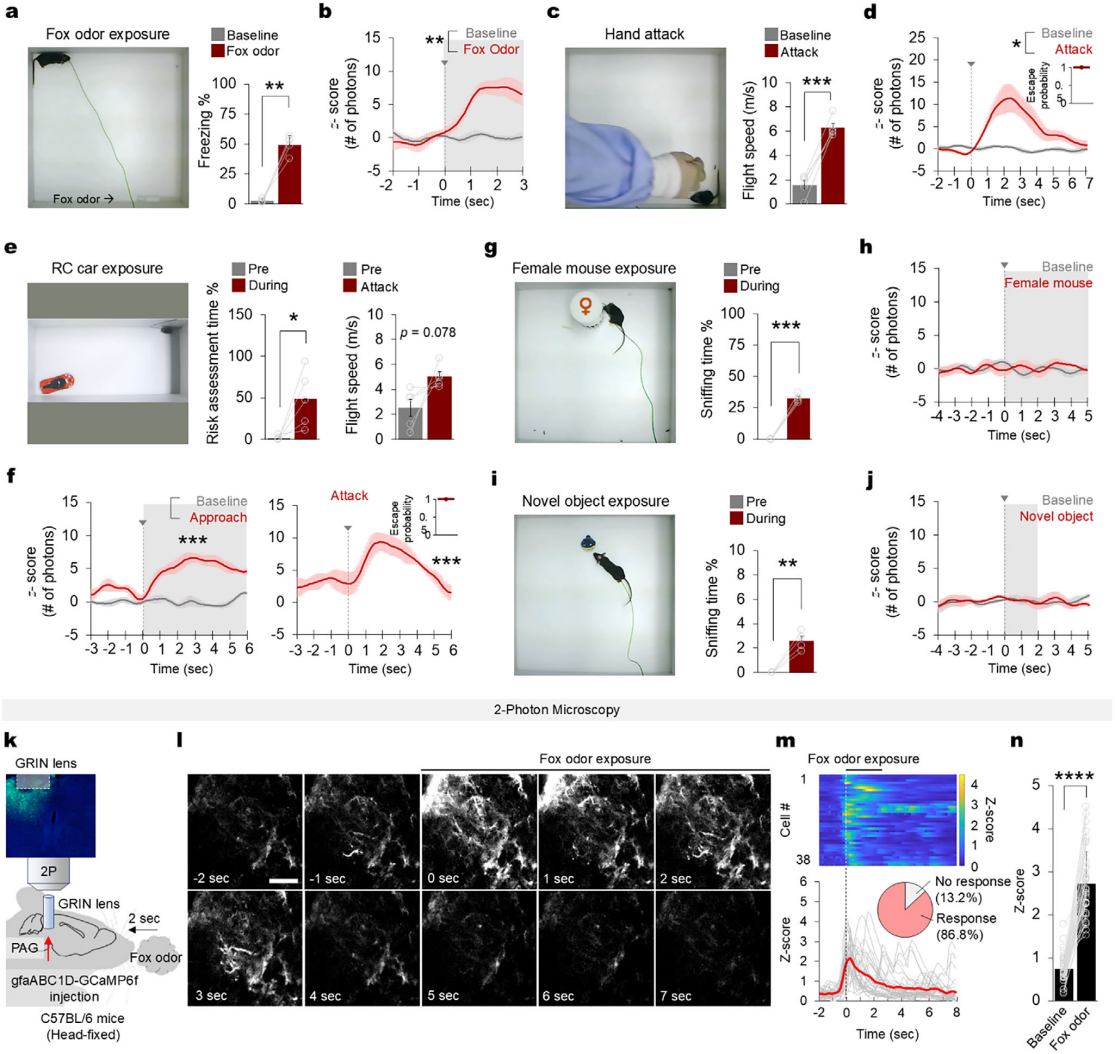
PAG astrocyte Ca^2+^ activity increases in threatening situations. (a) Fox odor exposure assay (left) and freezing (percentage of total time) upon fox odor exposure (right, *n* = 6, two‐tailed paired t‐test ^**^ *p* = 0.002). (b) The mean trace of PAG astrocyte Ca^2+^ activity (Gaussian filtered, *n* = 6 mice) presented as a z‐score before (baseline, gray line) and during (red line) the fox odor test. The onset of the fox odor exposure (0 s) is indicated with gray shading (*n* = 6, two‐tailed paired t‐test ^**^ *p* = 0.009). (c) The hand attack assay (left) and mouse flight speed before and upon hand attack (right, *n* = 7, Two‐tailed paired t‐test ^***^ *p* < 0.001). (d) PAG astrocyte Ca^2+^ activity before and upon hand attack (*n* = 7, two‐tailed paired t‐test ^*^ *p* < 0.012). Inset boxplot shows mice escape probability upon hand attack. (e) RC car assay (left), percentage duration of risk assessment behavior before and during RC car exposure (middle, Two‐tailed paired t‐test *p* = 0.043), and mouse flight speed before and upon RC car attack (right, *n* = 7, Two‐tailed paired t‐test *p* = 0.078). (f) PAG astrocyte Ca^2+^ activity before (baseline, gray line) and during risk assessment (approach, red line; left, two‐tailed paired t‐test *p* < 0.001). Right panel shows PAG astrocyte Ca^2+^ activity upon RC car attack (*n* = 7, two‐tailed paired t‐test ^***^ *p* < 0.001). Inset boxplot shows mice escape probability upon RC car attack. (g) Female mouse exposure assay (left) and the duration (percentage of total time) of sniffing behavior before and during exposure (right, *n* = 7, two‐tailed paired t‐test ^***^ *p* < 0.001) (h) PAG astrocyte Ca^2+^ activity before and during exposure to female mouse (*n* = 7, two‐tailed paired t‐test *p* = 0.437). (i) Novel object exposure assay (left) and the duration (percentage of total time) of mouse sniffing behavior before and during exposure to the novel object (right, *n* = 7, two‐tailed paired t‐test ^**^ *p* = 0.001). (j) PAG astrocyte Ca^2+^ activity before and during exposure to the novel object (*n* = 7, two‐tailed paired t‐test *p* = 0.959). (k) Experimental setup for two‐photon microscopy imaging of PAG astrocytes in head‐fixed mice. (l) Changes in Ca^2+^ activity in the PAG during 2‐second exposure to fox odor. Scale bar, 200 mm. (m) Ca^2+^ activity changes in individual PAG astrocytes (gray) and the mean activity (red). The inset diagram shows the percentage of responsive (86.8%) and non‐responsive (13.2%) astrocytes in the PAG region. (n) Z‐scored Ca^2+^ amplitudes were compared between the baseine period and the fox odor‐exposure period (two‐tailed paired t‐test ^****^ *p* < 0.0001). Data are presented as the mean ± s.e.m. See Table S1 for detailed values and statistics.

To test whether astrocytes in the PAG are involved in mouse defensive responses to threatening situations, we used TCSPC, a highly sensitive in vivo fiber photometry method (Figure 1a), to measure PAG astrocyte calcium activity as mice were exposed to various threat stimuli. The threat stimuli were validated by c‐Fos expression in the PAG neurons, as previously reported [17, 18] (Figure S1). First, we assessed astrocyte density and found that S100b^+^ astrocytes are evenly distributed along the PAG columns (Figure S2). We targeted PAG astrocytes with a local injection of adeno‐associated viruses (AAVs) expressing GfaABC_1_D‐GCaMP6f followed by the implantation of a TCSPC optic fiber probe (Figure S3a,b,h). The histology data show that GCaMP6 was mostly colocalized with S100b^+^ (> 96.5%) and GFAP^+^ (82.9%) astrocytes with negligible expression in NeuN^+^ neurons (< 0.6 %, Figure S3d,f). Then, we exposed those mice to various threatening situations and measured PAG astrocyte calcium activity during their defensive responses.

We exposed the mice to a predator odor, 2,5‐dihydro‐2,4,5‐trimethylthiazoline (TMT; Figure 1a, left panel), a component of fox feces (referred to as ‘fox odor’). Previous studies have shown that TMT induces fear and defensive behaviors in mice, indicating that it is an innate threat stimulus [28]. Compared with the baseline level (before the introduction of fox odor), exposure to fox odor increased mouse freezing responses (Figure 1a, right panel), which was accompanied by an increase in Ca^2+^ activity in PAG astrocytes (Figure 1b; Movie S1). In another assay, we applied a threat stimulus by quickly touching the back of each mouse with the experimenter's hand [29, 30], referred to as the “hand attack” (Figure 1c, left panel). The hand attack evoked escape or fleeing behaviors in the mice and a strong increase in PAG astrocyte Ca^2+^ activity (Figure 1c,d). We also exposed the mice to a remote‐controlled (RC) car (Figure 1e, left panel), which posed the innate threat of being a large moving object in an open arena [31, 32]. The presence of the RC car in the open arena (Figure 1e, left panel) evoked vigilance and risk assessment behavior in the mice (Figure 1e, middle panel). Mice who approached and assessed the risk posed by the RC car were randomly attacked by it, which led the mice to flee from it (Figure 1e, right panel; Movie S1). Correspondingly, the in vivo fiber photometry data show increases in PAG astrocyte Ca^2+^ activity when the mice started to assess the risk and approach the RC car (Figure 1f, left panel), and fleeing from the RC car attack evoked a strong increase in Ca^2+^ activity (Figure 1f, right panel) that returned to the baseline level when the mouse reached the corner of the open arena (Movie S1). This increase in Ca^2+^ activity was not observed when the mice were exposed to a small inanimate object (Figure 1i,j; Movie S2). These results indicate that PAG astrocytes respond to various threat stimuli by increasing the intracellular Ca^2+^ activity. On the other hand, PAG astrocyte Ca^2+^ activity did not increase from the baseline level when the mice interacted with a female conspecific or explored the open arena with a small novel object (Figure 1g–j; Movie S2), and did not correlate with mice locomotor activity in an open arena (Figure S3g), suggesting that the Ca^2+^ elevations in PAG astrocytes are specifically evoked by threatening stimuli. To measure Ca^2+^ activity in astrocytes at the single‐cell level, we performed two‐photon imaging of AAV‐GfaABC_1_D‐GCaMP6f‐injected mice with gradient refractive index (GRIN) lens implantation (Figure 1k). The majority of astrocytes (86.8%) exhibited a significant increase in Ca^2+^ activity immediately following fox odor exposure (Figure 1l–n). These data show that PAG astrocytes actively respond to threat stimuli.

### 2.2 PAG Astrocytes are Required for an Optimal Defensive Response to a Threat

**FIGURE 2:**
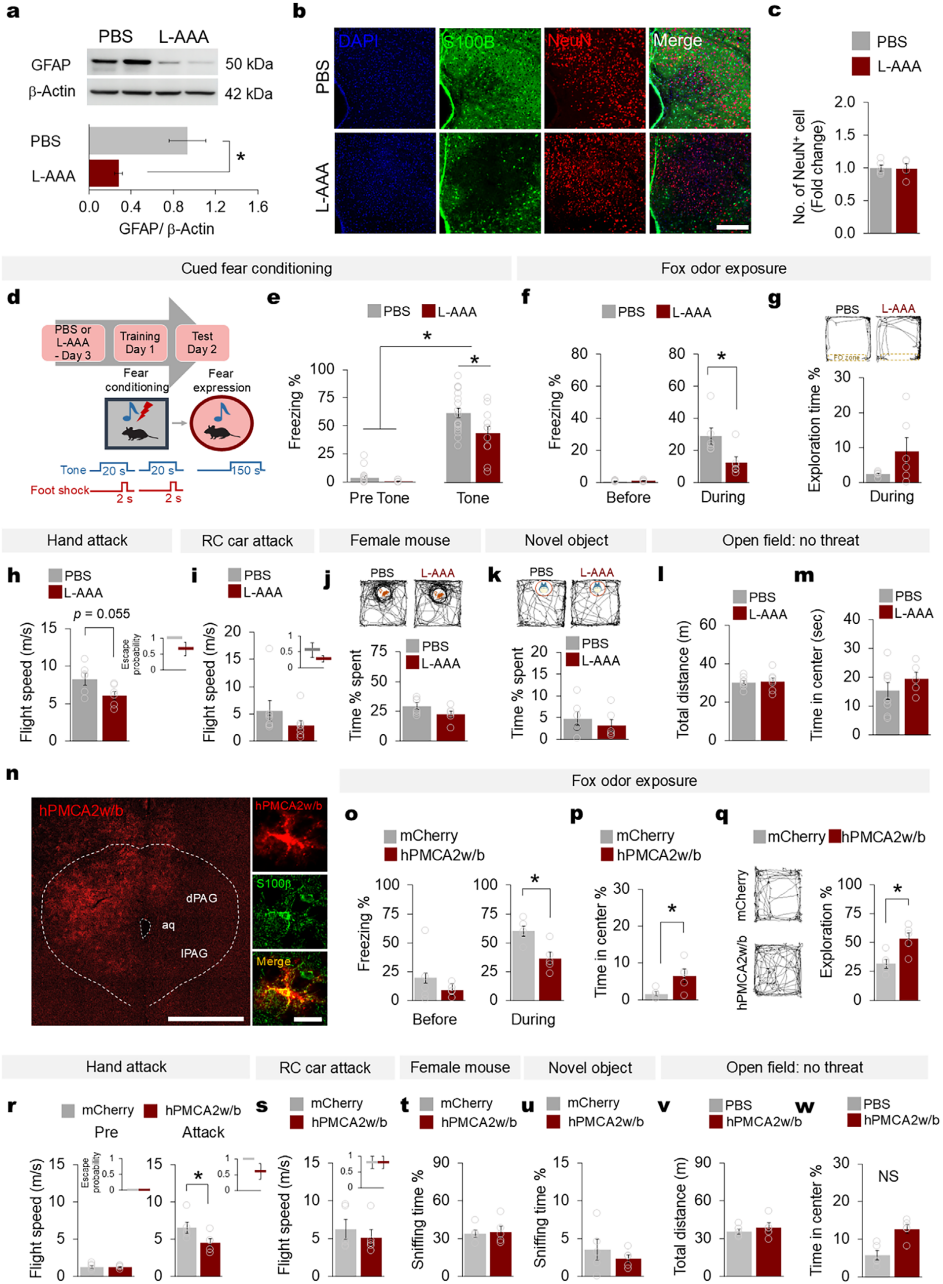
PAG astrocyte ablation reduces mouse optimal defensive response against threat. (a) Representative western blots of PAG GFAP and b‐actin expression in PBS‐ and L‐AAA‐injected mice (upper). Quantified GFAP expression over b‐actin presented as fold change between groups (bottom, *n* = 4 mice per group, two‐tailed Mann‐Whitney *U*‐test ^*^ *p* = 0.021). (b) Representative images of PAG tissue from mice injected with PBS or L‐AAA showing S100b^+^ astrocytes (green) and NeuN^+^ cells (red). Scale bar, 200 mm. (c) Quantification of NeuN^+^ cells in the PAG areas of mice injected with PBS (*n* = 5) or L‐AAA (*n* = 4, two‐tailed Mann‐Whitney *U*‐test *p* = 0.806). Fold‐change indicates the ratio of the mean of L‐AAA mice to the mean of PBS mice. The PBS error bar is computed using the standard error of the mean to account for and visualize variability of the data. (d) Experimental scheme for auditory fear conditioning of mice injected with PBS or L‐AAA. (e) Freezing (percentage of total time) in the auditory fear conditioning test: pre‐auditory cue exposure and auditory cue exposure sessions (PBS n = 11 mice, L‐AAA n = 18 mice, two‐way repeated ANOVA, ^***^ *p* < 0.001, followed by two‐tailed Mann‐Whitney *U*‐test, ^*^ *p* = 0.035). (f) Freezing (percentage of total time) before and during exposure to fox odor (PBS *n* = 6 mice, L‐AAA *n* = 6 mice; two‐way repeated ANOVA ^*^ *p* = 0.034 followed by two‐tailed Mann‐Whitney *U*‐test, ^*^ *p* = 0.025). (g) Sample trace from mice injected with PBS (*n* = 6) or L‐AAA (*n* = 6) as they explored an open arena with fox odor (upper). Percentage of exploration time during fox odor exposure (two‐tailed Mann‐Whitney *U*‐test, *p* = 0.262). (h) Flight speed against hand attack (PBS *n* = 5 mice, L‐AAA *n* = 5 mice; two‐tailed Mann‐Whitney *U*‐test, *p* = 0.055). Inset plot shows mouse escape probability upon hand attack. (i) Flight speed against RC car attack (PBS *n* = 7 mice, L‐AAA n = 8; two‐tailed Mann‐Whitney *U*‐test *p* = 0.105). Inset plot shows mouse escape probability from RC car attack. (j) Time spent with the female conspecific (Two‐tailed Mann‐Whitney *U*‐test *p* = 0.150) and (k) novel object (Two‐tailed Mann‐Whitney *U*‐test *p* = 0.423). Traces of mouse locomotor activity (upper panels, PBS *n* = 6 mice, L‐AAA *n* = 6). (l) The total distance (Two‐tailed Mann‐Whitney *U*‐test *p* = 0.728) and (m) time the mice spent in the center in the OFT (PBS *n* = 8, L‐AAA n = 7; two‐tailed Mann‐Whitney *U*‐test *p* = 0.487). (n) Representative image of GfaABC1D‐hPMCA2w/b (red) expression in PAG section (left) and the colocalization with S100b^+^ (green) astrocyte (right). Scale bar, 500 mm and 50 mm. (o) Freezing percentage before and during fox odor exposure (mCherry *n* = 5 mice, hPMCA2w/b *n* =5 mice, two‐way repeated ANOVA ^*^ *p* = 0.016, followed by two‐tailed Mann‐Whitney U‐test ^*^ *p* = 0.016). (p) Time that mice spent in the center (two‐tailed Mann‐Whitney U‐test ^*^ *p* = 0.047), and (q) the trace (left panel) and percentage of exploration time in an open arena during fox odor exposure (mCherry *n* = 5 mice, hPMCA2w/b *n* =5 mice; two‐tailed Mann‐Whitney U‐test ^*^ *p* = 0.016). (r) Flight speed of mice before (left panel, mCherry *n* = 5 mice, hPMCA2w/b *n* = 5 mice; two‐tailed Mann‐Whitney U‐test ^*^ *p* = 0.690) and during the hand attack (right panel, mCherry *n* = 5 mice, hPMCA2w/b *n* = 5 mice; two‐tailed Mann‐Whitney U‐test ^*^ *p* = 0.047). Inset plot shows mouse escape probability upon hand attack. (s) Flight speed of mice against the RC car attack (mCherry *n* = 5 mice, hPMCA2w/b *n* =5 mice; two‐tailed Mann‐Whitney U‐test *p* = 0.548). Inset plot shows mouse escape probability from RC car attack. (t) Percentage of sniffing time to a female mouse and (u) a novel object between groups (mCherry *n* = 5 mice, hPMCA2w/b *n* =5 mice; two‐tailed Mann‐Whitney U‐test *p* = 0.465). (v) The total distance (two‐tailed Mann‐Whitney U‐test *p* = 0.465) and (w) time the mice spent in the center of an open arena with no threat (mCherry *n* = 5 mice, hPMCA2w/b *n* = 5 mice; two‐tailed Mann‐Whitney U‐test *p* = 0.075). Data are presented as the mean ± s.e.m. See Table S1 for detailed values and statistics.

We next investigated whether PAG astrocytes are required for defensive responses to threatening situations. To this end, we pharmacologically ablated PAG astrocytes using L‐a‐aminoadipate (L‐AAA), a widely‐used astrotoxin [33, 34]. Injecting L‐AAA (20 mg/ml) into the mouse PAG area significantly reduced GFAP protein expression and the number of S100b^+^ astrocytes, compared with phosphate‐buffered saline (PBS)‐injected control mice (Figure 2a), without influencing the cell density of neurons (NeuN^+^), microglia (Iba1^+^), or oligodendrocytes (CC1^+^) (Figure 2b,c; Figure S4a,b). Two to three days after an L‐AAA injection, we exposed the mice to various threat stimuli and assessed their defensive behaviors. In the auditory fear conditioning test, the mice were placed in a chamber and received a foot shock associated with an auditory cue. After 24 h, the mice were placed in a novel chamber, and freezing behavior was recorded before and during the presentation of the conditioned auditory cue without the shock (Figure 2d). During presentation of the conditioned auditory cue, the L‐AAA‐injected mice showed a significantly reduced freezing response compared with the control mice (Figure 2e). Similarly, the L‐AAA‐injected mice showed decreased freezing and an increased tendency for exploration in the arena during exposure to fox odor, compared with the control mice (Figure 2f,g), and they showed significantly reduced flight speed against the hand attack and a tendency toward reduced flight speed against the RC car attack (Figure 2h,i). We did not observe any significant difference between the L‐AAA‐ and PBS‐injected mice in the amount of sniffing or time spent near a female conspecific (Figure 2j) or a novel object (Figure 2k), which were presented as non‐threating stimuli. The groups also did not vary in the total distance traveled in an empty open arena, suggesting that neither PBS nor L‐AAA injection had any effect on locomotor activity (Figure 2l, Figure S4d–f). The time spent in the center during the open field test (OFT) was also the same in the two groups (Figure 2m), as was the immobility time in the tail suspension test (TST) and forced‐swim test (FST) (Figure S4g,h). No differences were observed between the groups when they were subjected to contextual fear conditioning and the elevated plus maze (EPM) test (Figure S4i–l). To circumvent any putative effects of ablating astrocytes using L‐AAA, we took advantage of reducing astrocyte calcium activity in the PAG using GfaABC_1_D‐hPMCA2w/b, a plasma membrane Ca^2+^ pump which extrude cytosolic Ca^2+^ (Figure 2n; Figure S4m,n) [35]. Our data showed that inhibition of PAG astrocyte calcium activity by hPMCA2w/b exerts similar behavioral effects to L‐AAA‐induced astrocyte ablation under various threat conditions (Figure 2o–w, Figure S4o–z). Taken together, these results suggest that PAG astrocytes are specifically involved in and required for optimal defensive responses against threat stimuli.

### 2.3 Optogenetic PAG Astrocyte Activation Evokes Defensive‐Like Responses

**FIGURE 3:**
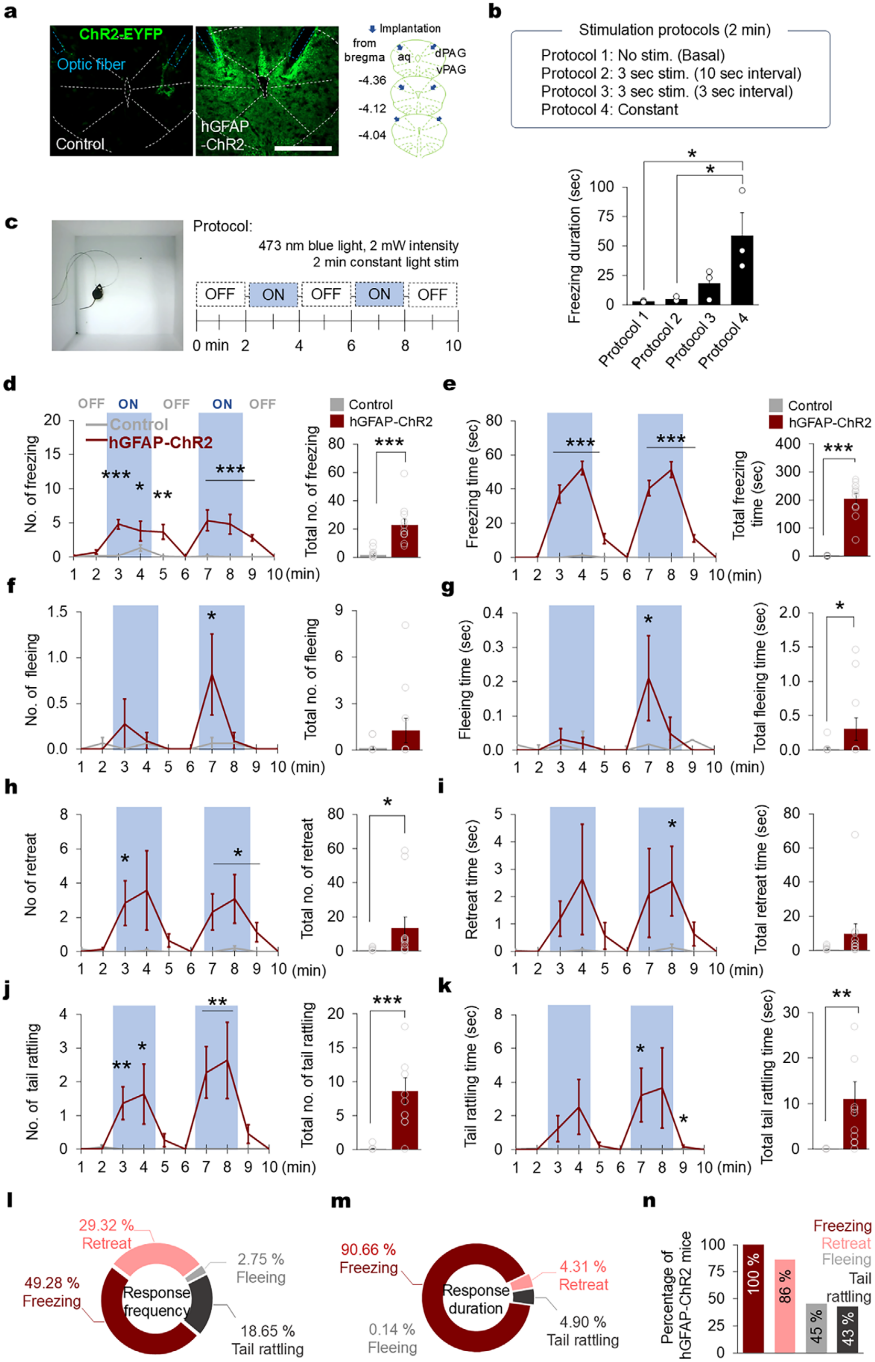
Optogenetic PAG astrocyte activation evokes defensive‐like responses. (a) Representative images of ChR2‐eYFP expression (green) and optic fiber implantation (blue traces) in the PAG tissue of control (vehicle‐injected, left) and hGFAP‐ChR2 (tamoxifen‐injected, middle panel) animals. Scale bar, 500 mm. Optic fiber placement in the dorsal PAG indicated by blue arrow. Implantations were found in slices from 4.04 mm to –4.36 mm posterior to the bregma (right panel). (b) Optogenetic stimulation protocol for PAG astrocytes and the effect on freezing behavior in mice. (c) Experimental protocol for optogenetic PAG astrocyte stimulation (right), which consisted of exposure to constant 473 nm light in 2 min light‐off and 2 min light‐on cycles for a 10 min session in an empty open arena (left). (d) Number of freezing events (left) in the control and hGFAP‐ChR2 mice in each minute across the 10 min session of light‐off and light‐on (shaded area) epochs and the total number of freezing events (right panel) among the mice in the whole 10 min session. (e) The duration of the freezing response (left panel) in each minute of the session and the total duration of freezing among the control and hGFAP‐ChR2 mice in the whole 10 min session. (f) The number of events and (g) duration of the fleeing response in control and hGFAP‐ChR2 mice during the light‐off and light‐on epochs. (h) The number of events and (i) duration of retreat or avoidance responses among the control and hGFAP‐ChR2 mice during light‐off and light‐on epochs. (j) The number of events and (k) duration of the tail rattling response among the control and hGFAP‐ChR2 mice during light‐off and light‐on epochs. (l) The frequency of all defensive responses (percentage of all actions) observed in hGFAP‐ChR2 mice during light‐on epochs. (m) The duration of all defensive‐like behaviors observed during light‐on epochs as a percentage of the total time. (n) The percentage of hGFAP‐ChR2 mice who displayed defensive‐like freezing, retreating, fleeing, and tail rattling behaviors (*n* = 11 hGFAP‐ChR2 mice). (d–k) Control *n* = 16 mice (gray), hGFAP‐ChR2 *n* = 11 mice (red), two‐tailed Mann Whitney *U*‐test between control and hGFAP‐ChR2, ^*^ *p* < 0.05, ^**^ *p* < 0.01, ^***^ *p* < 0.001. Data are presented as the mean ± s.e.m. See Table S1 for detailed values and statistics.

We next evaluated the role of PAG astrocytes by using optogenetics to explore whether PAG astrocytes directly influence mouse defensive responses. We generated astrocyte‐specific channelrhodopsin 2 (ChR2)‐expressing mice (hGFAP‐ChR2) by crossing hGFAP‐CreERT2 mice with floxed‐stop‐ChR2‐eYFP mice (Figure S5a) [36]. In these mice, ChR2 expression was colocalized with GFAP^+^ and S100b^+^ astrocytes, not with NeuN^+^ neurons or Iba‐1^+^ microglia (Figure S5b–d). We confirmed that blue light stimulation increased intracellular Ca^2+^ activity in ChR2‐expressing astrocytes both in primary cultured astrocytes (Figure S5e,f), and acute PAG slice (Figure S5g–i). By implanting an optic fiber in the dorsal PAG area (Figure 3a; Figure S5j), we explored whether stimulating dorsal PAG astrocytes with optogenetics could prompt behavioral changes in mice placed in an open arena (Figure 3b,c). We applied a 3‐second optogenetic stimulus based on previous findings that Ca^2+^ activity in astrocyte peaks around 3 s following exposure to a threat (Figure 1). Our results showed that shorter stimulation intervals significantly increased freezing behavior in mice (Figure 3b). Consequently, we opted to use continuous stimulation (2 mW, 473 nm) to maintain this effect. During 2‐minute light‐on epochs, hGFAP‐ChR2 mice manifested significant increases in the frequency and duration of the freezing response, characterized as drastic immobility in an inflexible posture, aside from breathing (Figure 3d,e; Movie S3). The freezing response of the hGFAP‐ChR2 mice gradually returned to the baseline level when the light was turned off. The increase in the freezing response was not seen in the control mice. In addition, tamoxifen injection per se did not affect mice’ locomotor activity (Figure S5k–n). The total duration and total number of freezing responses in each 10‐min session was significantly higher in the hGFAP‐ChR2 mice than the control mice (Figure 3d,e; bar graphs). We also observed other defensive‐like behaviors in between freezing bouts. The hGFAP‐ChR2 mice manifested increased duration and frequency of fleeing, which involved jumping and rapid changes of direction, during light‐on epochs, which were not observed in the light‐off epochs or the control mice (Figure 3f,g; Movie S3). Also, an increased frequency and duration of retreat or avoidance‐like responses, defined as backward movements, were observed during the light‐on epochs (Figure 3h,i; Movie S3). Lastly, we noticed a tail‐rattling response during light‐on epochs that is putatively a territorial and threat behavior [37] and is characterized by a stiff tail followed by a fast waving movement (Movie S3). Light stimulation evoked a significant increase in both the number and duration of tail‐rattling episodes in the hGFAP‐ChR2 mice but not in the control mice (Figure 3j,k). Among the different defensive‐like behaviors evoked by optogenetic PAG astrocyte stimulation, the mice manifested freezing behavior with highest frequency and duration (Figure 3l,m). Freezing was consistently observed in all the hGFAP‐ChR2 mice receiving optogenetic stimulation that were used in this experiment (Figure 3n). To explore the putative involvement of ventral PAG (vPAG) astrocytes, we conducted additional experiments with optogenetic stimulation of vPAG. We observed that activating astrocytes in the vPAG elicited pronounced freezing and other defensive behaviors in response to light stimulation in the open arena (Figure S6a–d). These findings suggest that all PAG columns may contribute to the observed effects through a gap junctional network. In addition, for more physiologically relevant astrocyte stimulation, we utilized melanopsin, a G‐protein‐coupled photopigment, to trigger Ca^2+^ signaling in PAG astrocytes [38]. We found that PAG astrocytes stimulation through melanopsin evoked similar defensive responses such as increased freezing and risk assessment through rearing behaviors in mice in the open arena (Figure S6e–h). These data show that the selection of PAG‐induced defensive actions by the mice was affected by our manipulations of PAG astrocyte activity. Meanwhile, optogenetic stimulation of PAG astrocytes did not affect mouse depressive‐ (Figure S7a–c) or anxiety‐like behaviors (Figure S7d–g). Neither did it affect mouse preference in the conditioned place preference (CPP) test (Figure S7h–l) or real‐time place preference (RTPP) test (Figure S7m–o), although defensive‐like behaviors were observed during conditioning. Taken together, our results suggest that optogenetic stimulation of PAG astrocytes is sufficient to evoke defensive‐like responses even in the absence of a threat stimulus.

### 2.4 Optogenetic Stimulation of PAG Astrocytes Disrupts Mouse Safety‐Seeking Behavior in a Threatening Environment

**FIGURE 4:**
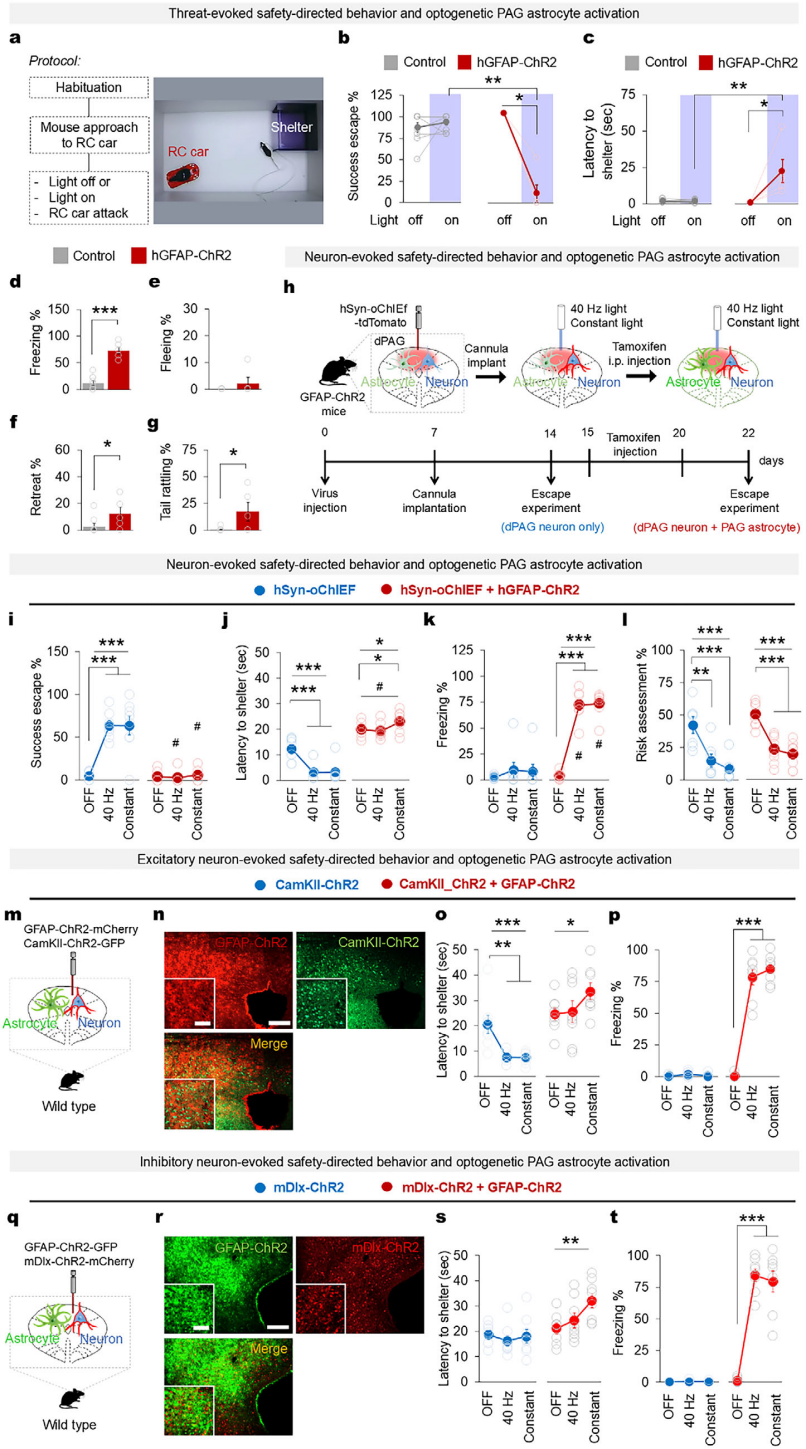
Optogenetic PAG astrocyte activation disrupts mouse safety‐directed behavior in a threatening environment. (a) The behavioral protocol (left) that started with mouse habituation to an arena containing only the shelter. On the test day, an RC car was placed in the arena with the shelter (right). When a mouse approached the RC car, it received light stimulation followed by an RC car attack to induce its escape to the shelter. (b) Percentage of successful escapes in light‐off and light‐on (shaded) epochs among the control (*n* = 9 mice, Wilcoxon signed rank test *p* = 0.465 (off vs on)) and hGFAP‐ChR2 mice (*n* = 7 mice; Wilcoxon signed rank test ^*^ *p* = 0.035 (off vs on), two‐tailed Mann‐Whitney *U* ‐test *p* = 0.002 (Control vs hGFAP‐ChR2)). A successful escape was counted when a mouse reached the shelter within 5 s of the onset of an RC car attack. (c) The time it took for the mice to reach the shelter after the onset of an RC car attack during light‐off and light‐on epochs (Control *n* = 9 mice, Wilcoxon signed rank test *p* = 0.859 (off vs on), hGFAP‐ChR2 *n* = 7 mice Wilcoxon signed rank test ^*^ *p* = 0.043 (off vs on), two‐tailed Mann‐Whitney *U* ‐test ^**^p = 0.003 (Control vs hGFAP‐ChR2)). (d) Percentage of time spent freezing (two‐tailed Mann‐Whitney *U* ‐test ^***^p < 0.001), (e) fleeing (two‐tailed Mann‐Whitney *U* ‐test *p* < 0.180), (f) retreating (two‐tailed Mann‐Whitney *U* ‐test ^*^ *p* = 0.039), and (g) tail rattling (two‐tailed Mann‐Whitney *U* ‐test ^*^ *p* = 0.037) during light stimulation (control *n* = 9 mice, hGFAP‐ChR2 *n* = 7 mice). (h) Experimental timeline for PAG neuron and astrocyte stimulation in an escape experiment. Non‐tamoxifen‐injected hGFAP‐ChR2 transgenic mice were injected with hSyn‐oChIEF‐mCherry, and the optic fiber was implanted 7 days post‐surgery. After a 7‐day recovery, the neuron‐induced escape experiment was performed (blue, ‘hSyn‐oChIEF’ = only neuron stimulation). Then, the same mice were injected with tamoxifen to optogenetically stimulate astrocytes (in addition to neurons) upon exposure to 2 mW 40 Hz light for 10 ms or constant (red, ‘hSyn‐oChIEF + hGFAP‐ChR2’ = neuron and astrocyte stimulation). (i) Percentage of successful escapes (scored when a mouse reached the shelter within 5 s of the onset of light‐on stimulation). (j) Latency to reach the shelter, (k) percentage of time spent freezing, and (l) risk assessment during light‐off and 40 Hz 10 ms and constant light‐on epochs. (m) Virus injection scheme for stimulation of excitatory neurons and astrocytes in the PAG. (n) Confirmation of virus expression in the PAG. Scale bar, 200 mm and 100 mm (inset). (o) Latency to reach the shelter and (p) percentage of time spent freezing during light‐off, 40 Hz (10 ms pulse), and constant light‐on epochs. (q) Virus injection scheme for stimulation of inhibitory neurons and astrocytes in PAG. (r) Confirmation of virus expression in the PAG. Scale bar, 200 mm and 100 mm (inset). (s) Latency to reach the shelter and (t) percentage of time spent freezing during light‐off, 40 Hz (10 ms pulse), and constant light‐on epochs. (i–t) hSyn‐oChIEF *n* = 8 mice; hSyn‐oChIEF + hGFAP‐ChR2 *n* = 6 mice; CamKII‐ChR2, CamKII‐ChR2 + hGFAP‐ChR2, mDlx‐ChR2, mDlx‐ChR2 + GFAP‐ChR2 *n* = 8 mice, one‐way ANOVA followed by LSD post hoc analysis, ^*^ *p* < 0.05, ^**^ *p* < 0.01, ^***^ *p* < 0.001. Wilcoxon signed rank test were used to compare hSyn‐oChIEF and hSyn‐oChIEF + hGFAP‐ChR2, ^#^ *p* < 0.05. Data are presented as the mean ± s.e.m. See Table S1 for detailed values and statistics.

Upon the detection of a threat or exposure to a potentially dangerous environment, it is an instinctive behavior for mice to enact a defensive response in order to secure their safety and maximize their potential for survival [6, 7, 18, 39]. Therefore, we exposed the mice to an open arena containing a shelter (safe zone) where they could hide and escape. The shelter was placed opposite the attacking RC car, and we tested the effects of PAG astrocyte stimulation on the natural behavior of mice to escape and seek safety in a potentially threatening environment (Figure 4a). The trials were classified as a successful escape if the mouse reached the shelter within 5 s after the onset of the RC car attack. The control mice made rapid and successful escapes to the shelter upon the RC car attack in both the light‐off and light‐on epochs (Figure 4b; Movie S4). Interestingly, the hGFAP‐ChR2 mice failed to escape and displayed significantly longer latency in reaching the shelter during light‐on epochs than in light‐off epochs (Figure 4b,c; MovieS4). The mouse shelter‐directed safety‐seeking behavior in the presence of an innate threat or a potentially harmful stimulus was disrupted by the freezing, retreat, fleeing, and tail rattling behavior evoked by PAG astrocyte stimulation (Figure 4d–g; Movie S4). We also found that the control mice naturally preferred and escaped to the shelter rather than exploring the open arena (Figure S8a,b; Movie S5). However, optogenetic PAG astrocyte stimulation disrupted the shelter‐directed escape of mice in the anxiogenic open arena (Figure S8b; Movie S5); those mice took a significantly longer time to reach shelter (Figure S8c) due to the defensive‐like behaviors evoked by optogenetic PAG astrocyte stimulation (Figure S8d–g). Therefore, we reasoned that optogenetic stimulation of PAG astrocytes evoked aberrant defensive‐like behaviors that defied the goal of defensive behavior, which is to reach safety and maximize the chance of survival.

### 2.5 Optogenetic PAG Astrocyte Activation Disrupts Dorsal PAG Neuron‐Evoked Shelter‐Directed Escape

We then investigated the effects of PAG astrocyte activation on the neural substrate of the dorsal PAG‐evoked defensive escape to safety. Recent studies have demonstrated that optogenetic stimulation of dorsal PAG glutamatergic neurons evokes shelter‐directed safety‐seeking escape behaviors [3, 7]. Thus, we investigated whether the innate murine shelter‐directed defensive escape instigated by dorsal PAG neuron activation is affected by PAG astrocyte stimulation by simultaneously manipulating the activity of both PAG astrocytes and neurons. First, to activate dorsal PAG neurons, we injected hGFAP‐ChR2 mice with AAVs encoding hSyn‐oChIEF tagged with tdTomato (Figure 4h, Figure S9a, upper panel). The expression of hSyn‐oChIEF‐tdTomato was mostly colocalized with NeuN^+^ neurons (> 88.83%) and not with S100b^+^ astrocytes (< 3.1%; Figure S9b,c). Seven days after virus injection, we implanted an optic fiber and let the mice recover for another 7 days. Then, we performed dorsal PAG neuronal stimulation in an escape paradigm (Figure S9d). Similar to a previous report, optogenetic activation of dorsal PAG neurons using 40 Hz (10 ms, 2 mW) light stimulation evoked an immediate escape to shelter [7] with significantly reduced risk assessment of the stimulation zone and latency in reaching the shelter compared with controls (Figure 4i,j left panels; Movie S6). This dorsal PAG neuronal stimulation dominantly produced shelter‐directed escape behavior. The constant light that we previously used for astrocyte stimulation (Figure 3) also elicited a similar behavioral response (Figure 4i,j left panels; Movie S6). We next injected the same hGFAP‐ChR2 mice with tamoxifen for 5 days to induce ChR2 expression in their astrocytes (Figure S9a, lower panel, e–f), and then we simultaneously stimulated PAG neurons and astrocytes in an escape paradigm. Surprisingly, the shelter‐directed escape induced by dorsal PAG neuron stimulation was completely abrogated by simultaneous PAG astrocyte stimulation (Figure 4i, right panel; Movie S6). The mice showed increased latency in reaching the shelter (Figure 4j, right panel). The prolonged latency and disrupted escape to shelter was due to a significant increase in freezing behavior upon light stimulation, which was not observed during dorsal PAG neuronal stimulation alone (Figure 4k,l). The behavioral changes were not observed in light‐off epochs or control mice injected with control virus and vehicle (Figure S9g–j). In our data, c‐Fos positive neurons were preferentially co‐localized with excitatory neurons (CamKII^+^) rather than inhibitory neurons (GABA^+^), suggesting a potential involvement of excitatory neurons in this behavior (Figure S1d,e). We therefore tested the same behavioral paradigm (Figure 4h) by expressing ChR2 in excitatory neurons (CamKII‐positive) or inhibitory neurons (mDlx‐positive) to determine whether these populations exert distinct or opposing functional effects (Figure 4m,n; Figure 4q,r). Selective activation of CamKII‐positive neurons robustly enhanced escape behaviors (Figure 4o), whereas simulation of mDlx‐positive neurons did not produce measurable changes in escape (Figure 4s). Notably, concurrent activation of astrocytes abolished the escape‐promoting effect of CamKII‐driven stimulation and, more broadly, suppressed escape even when mDlx‐ChR2 was present, indicating that astrocytic activation exerts a dominant suppressive influence on defensive actions (Figure 4o,p and Figure 4s,t). In addition, the ablation of PAG astrocytes similarly disrupted dorsal PAG neuron‐evoked shelter‐directed safety‐seeking behaviors (Figure S10, Movie S7). This further shows the possible modulatory role of PAG astrocytes in murine goal‐directed defensive and escape behaviors. Taken together, our data show that the PAG requires not only neurons but also astrocytes to produce an optimal defensive response to a threatening environment, and aberrant PAG astrocyte activation in mice can dysregulate defensive behaviors in ways that resemble panic‐like responses, such as the failure or disruption of safety‐seeking behavior in threatening situations [18].

### 2.6 PAG Astrocyte Ca^2+^ Activity Aberrantly Increases in a CO_2_‐Mediated Panicogenic Environment, and Their Inhibition Rescued Mice from CO_2_‐ or fox Odor‐Induced Panic‐Like Behavior

**FIGURE 5:**
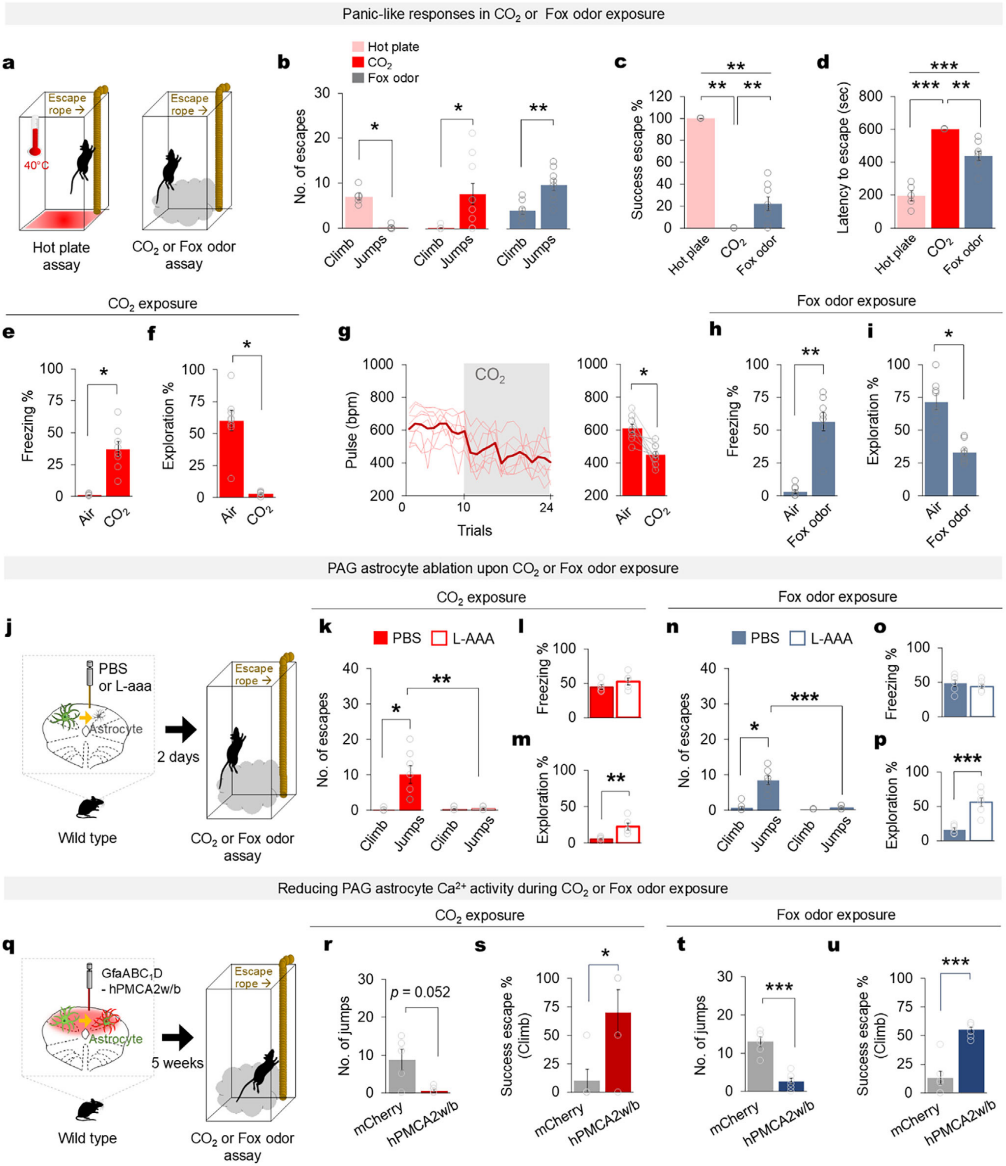
Inhibition of PAG astrocyte Ca^2+^ activity rescued mice from CO_2_‐induced panic‐like state. (a) Experimental scheme for the hot plate (left) and CO_2_ assays (right) in which the mice were given an escape route. (b) Number of escapes: climbs and jumps during hot plate (*n* = 5 mice, Wilcoxon signed‐rank test ^*^ *p* = 0.042), CO_2_ assays (*n* = 10 mice, Wilcoxon signed‐rank test ^*^ *p* = 0.011), and fox odor (*n* = 8 mice, Wilcoxon signed‐rank test ^**^ *p* = 0.0078). (c) Percentage of successful escapes during the hot plate (*n* = 5 mice), CO_2_ assays (*n* = 5 mice), and fox odor (n = 8 mice, one‐way ANOVA followed by LSD post hoc analysis, ^**^ *p* < 0.01). (d) Latency to escape during exposure to the hot plate (n = 5), CO_2_ assays (n = 5), and fox odor (n = 8, one‐way ANOVA followed by LSD post hoc analysis, ^**^ *p* < 0.001, ^***^ *p* < 0.0001). (e) Percentage of time spent freezing in the normal air and CO_2_ exposure conditions (*n* = 8 mice, Wilcoxon signed‐rank test, ^*^ *p* = 0.012). (f) Percentage of time the mice spent exploring during exposure to normal air versus CO_2_ (*n* = 8 mice, Wilcoxon signed‐rank test, ^*^ *p* = 0.012). (g) Recorded pulse rate (beats per minute) before and during CO_2_ exposure during 24 trials (left) and the average pulse rate before (air exposed) and during CO_2_ exposure (*n* = 8 mice, Wilcoxon signed‐rank test, ^*^ *p* = 0.012). (h) Percentage of time spent freezing under normal air and fox odor conditions (*n* = 8 mice, Wilcoxon signed‐rank test, ^**^ *p* < 0.01). (i) Percentage of time spent exploring during exposure to normal air versus fox odor (*n* = 8 mice, Wilcoxon signed‐rank test, ^*^ *p* < 0.05). (j) Schematic for PAG astrocyte ablation and the CO_2_ assay with an escape route, 2 days post‐surgery. (k) Number of escapes (climbs and jumps) during CO_2_ exposure by mice injected with PBS (*n* = 7 mice, Wilcoxon signed‐rank test *p* = 0.018) or L‐AAA (*n* = 7 mice, Wilcoxon signed‐rank test, *p* = 0.655, two‐tailed Mann‐Whitney *U* ‐test, ^**^ *p* = 0.001 PBS vs L‐AAA jumps). (l) Percentage of time that the PBS‐ (n = 5) or L‐AAA‐injected mice (n = 5) spent freezing (two‐tailed Mann‐Whitney *U* ‐test, *p* = 0.347) and (m) exploring during the CO_2_ assay (two‐tailed Mann‐Whitney *U* ‐test, ^**^ *p* = 0.009). (n) Number of escape behavior (climbs and jumps) during fox odor exposure in PBS‐injected mice (n = 6 mice, Wilcoxon signed‐rank test ^*^ *p* < 0.05) and L‐AAA‐injected mice (*n* = 6 mice, Wilcoxon signed‐rank test, *p* = 0.500, two‐tailed Mann‐Whitney *U* ‐test, ^***^ *p* < 0.001 PBS vs L‐AAA jumps). (o) Percentage of time that PBS‐ (*n* = 6) or L‐AAA‐injected mice (*n* = 6) spent freezing during fox odor exposure (two‐tailed Mann‐Whitney *U* ‐test). (p) Percentage of time spent exploring during the fox odor exposure (two‐tailed Mann‐Whitney *U* ‐test, ^***^ *p* < 0.001). (q) Schematic for the inhibition of PAG astrocyte Ca^2+^ activity through GFaABC1D‐hPMCA2w/b and the CO_2_ assay with an escape route 5 weeks post surgery. (r) The number of jumps between mCherry (*n* = 5 mice) and hMPCA2w/b (*n* = 5 mice) during CO_2_ assay (two‐tailed Mann‐Whitney *U* ‐test, ^*^ *p* = 0.052). (s) The percentage of successful escapes through climbing the escape route in mCherry (*n* = 5 mice) and hPMCA2w/b (*n* = 5 mice) during CO_2_ assay (two‐tailed Mann‐Whitney *U* ‐test, * *p* = 0.041). (t) Number of jumps during fox odor exposure in mCherry‐expressing mice (*n* = 6) and hMPCA2w/b‐expressing mice (n = 6) (two‐tailed Mann‐Whitney *U* ‐test, ^***^ *p* < 0.001). (u) Percentage of successful escapes achieved by climbing the escape route in mCherry (n = 6) and hPMCA2w/b (*n* = 6) mice during fox odor exposure (two‐tailed Mann‐Whitney *U* ‐test, ^***^ *p* < 0.001). Data are presented as the mean ± s.e.m. See Table S1 for detailed values and statistics.

The dysregulated behavioral features observed in the PAG astrocyte‐activated mice resemble maladaptive fear and panic‐related behaviors [18, 40]. Therefore, we next investigated whether PAG astrocytes are involved in panic‐related behaviors by exposing mice to CO_2_, and predator odor (fox odor), a well‐established panicogenic agent used in both human and animal studies [17, 18, 41, 42, 43]. In previous reports, CO_2_‐exposed mice showed a significant increase in jumping, intermittent freezing, and failure to escape, even when an escape route was available [18, 40]. Similarly, we subjected mice to a chamber with a hot plate, or a chamber with CO_2_ or fox odor and provided an escape route (Figure 5a). As previously reported, the mice exposed to the hot plate escaped through an increased number of climbs, and the mice exposed to CO_2_ or fox odor displayed aimless jumping (Figure 5b). A significantly higher percentage of mice exposed to the hot plate successfully escaped and with significantly reduced latency compared with the mice exposed to the panicogenic CO_2_ or fox odor (Figure 5c, d). The mice exposed to CO_2_ not only showed panic‐related jumping but also increased freezing (Figure 5e) and reduced explorative behavior (Figure 5f). Moreover, the panicogenic CO_2_ exposure significantly reduced mouse pulse rates (Figure 5g), which is a previously reported panic‐related physiological change in mice [41, 44]. Similarly, mice exposed to fox odor exhibited both panic‐related jumping and increased freezing (Figure 5h), along with reduced explorative behavior (Figure 5i). In the panicogenic environment using CO_2_, PAG astrocyte ablation significantly reduced panic‐related jumping (Figure 5j,k), but it made no difference in freezing behavior (Figure 5l). It also significantly increased exploratory behavior compared with the PBS‐injected mice (Figure 5m). In addition, in the panicogenic environment induced by fox odor, PAG astrocyte ablation significantly reduced panic‐related jumping (Figure 5n), without altering freezing behavior (Figure 5o). It also markedly increased exploratory behavior compared with PBS‐injected mice (Figure 5p). Notably, the in vivo Ca^2+^ recordings show a robust increase in PAG astrocyte Ca^2+^ activity in the mice upon exposure to CO_2_ (Figure S11a). Remarkably, the fox odor and CO_2_‐evoked Ca^2+^ activity increase in PAG astrocytes was higher than the Ca^2+^ activity elicited by the other threat stimuli that we used to trigger mouse defensive behaviors (Figure S11b–d). Furthermore, optogenetic activation of PAG astrocytes reduced the pulse rates to a level similar to that upon panicogenic CO_2_ exposure (Figure S12). Notably, inhibiting PAG astrocyte Ca^2+^ activity with hPMCA2w/b rescued mice from CO_2_‐ or fox odor‐induced panic‐like behaviors, allowing them to successfully escape from the CO_2_‐ or fox odor‐infused chamber using a rope (Figure 5q–u, Movie S8). These results suggest that PAG astrocytes are actively engaged in CO_2_‐ or fox odor‐induced panic‐related behavior.

### 2.7 PAG Astrocytes Modulate Mouse Defensive and CO_2_‐induced Panic‐Like Behaviors Through ATP Release

**FIGURE 6:**
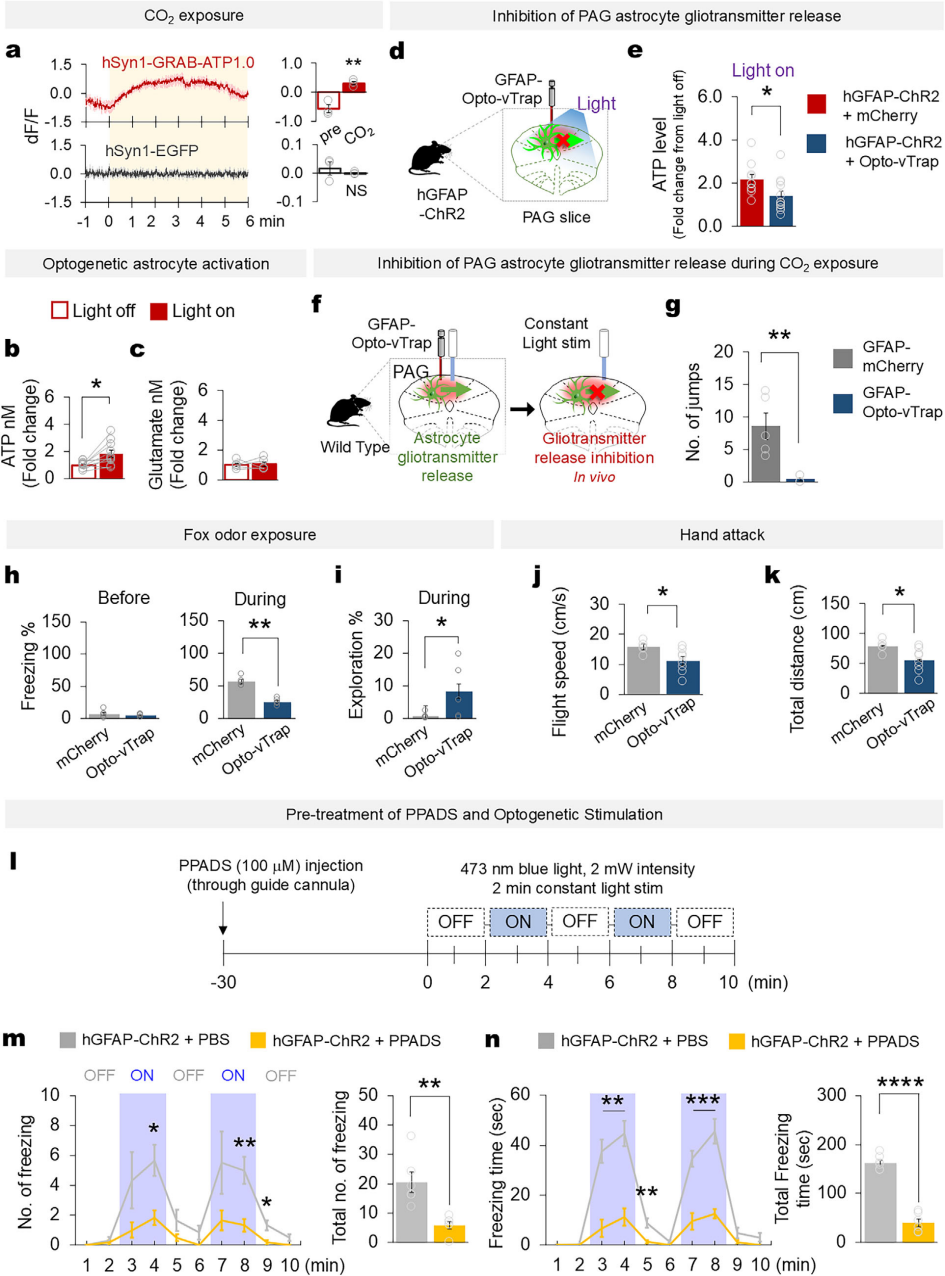
PAG astrocytes modulate defensive and panic‐like behaviors through ATP release. (a) In vivo fiber photometry recording of GRAB ATP1.0 sensors (upper panel, *n* = 3 mice) and control virus (lower panel, *n* = 3) in mouse PAG during CO_2_ exposure (left panel), and the average dF/F before and during Co2 exposure in GRAB‐ATP1.0 sensor‐injected (Two‐tailed paired t‐test *p = 0.043) and control virus‐injected mice (Two‐tailed paired t‐test *p* = 0.578) (b) Extracellular ATP levels in hGFAP‐ChR2 PAG astrocytes in light‐off and light‐on conditions (*n* = 10 mice, Wilcoxon signed‐rank test, *p = 0.013) (c) Extracellular glutamate levels during light‐off and light‐on epochs (*n* = 5 mice, Wilcoxon signed‐rank test, *p* = 0.799). Fold‐change indicates the ratio of the mean of ‘light on’ to the mean of ‘light off’. ‘Light off’ error bar is computed using the standard error of the mean to account for and visualize variability of the data. (d) Schematic of PAG astrocyte gliotransmitter inhibition via an injection of control virus (GFAP‐mCherry) or GFAP‐Opto‐vTrap‐mCherry into hGFAP‐ChR2 mice. Light stimulation was delivered to PAG slices in which the astrocytes expressed both ChR2 and Opto‐vTrap, and extracellular ATP levels were measured. (e) Extracellular ATP levels from GFAP‐mCherry (*n* = 10 mice) and Opto‐vTrap‐injected hGFAP‐ChR2 (*n* = 12 mice) PAG slices during light‐on epochs (Two‐tailed Mann‐Whitney *U* ‐test, ^*^ *p* = 0.025). (f) Schematic of protocol for in vivo PAG astrocyte gliotransmitter release inhibition. (g) The number of panic‐related jumping events during CO_2_ exposure among GFAP‐mCherry‐ (*n* = 5 mice) and GFAP‐Opto‐vTrap‐injected (*n* =5) mice (Two‐tailed Mann‐Whitney *U* ‐test, ^*^ ^*^p = 0.008). (h) Percentage of freezing before (left panel, two‐tailed Mann‐Whitney *U* ‐test, *p* = 0.465) and during (right panel, two‐tailed Mann‐Whitney *U* ‐test, ^**^ *p* = 0.009) the fox odor exposure (mCherry *n* = 5 mice, Opto‐vTrap *n* = 5 mice). (i) Percentage of exploration time in an open arena during the fox odor exposure (two‐tailed Mann‐Whitney *U* ‐test, *p = 0.028). (j) Flight speed (two‐tailed Mann‐Whitney *U* ‐test, ^*^ *p* = 0.028) and (k) total distance traveled (two‐tailed Mann‐Whitney *U* ‐test, ^**^ *p* = 0.047) to avoid the threat stimulus among wild‐type mice injected with GFAP‐mCherry (*n* = 5 mice) or GFAP‐Opto‐vTrap (*n* = 8 mice) and subjected to hand attack. (l) Behavioral paradigm for optogenetic stimulation of PAG astrocytes following PPADS pre‐treatment. (m) Number of freezing events (left) in control (PBS‐injected) and hGFAP‐ChR2 (PPADS‐injected) mice during each minute of a 10 min session consisting of light‐off and light‐on (shaded area) epochs, and total freezing events (right) across the entire session (*n* = 6 in each group). (n) Duration of freezing (left) in each minute of the session and total freezing duration (right) across the 10‐min session in control and hGFAP‐ChR2 mice (Two‐tailed Mann Whitney *U*‐test between PBS and PPADS, ^*^ *p* < 0.05, ^**^ *p* < 0.01, ^***^ *p* < 0.0001). Data are presented as the mean ± s.e.m. See Table S1 for detailed values and statistics.

To investigate the underlying mechanism involved in the PAG upon exposure of mice to CO_2_, we tested putative gliotransmitter release through in vivo fiber photometry in mice injected with GRAB sensors. Our data show that upon infusion of CO_2_ in chamber, hSyn1‐GRAB‐ATP signal was increased in the PAG (Figure 6a), indicating that ATP level rises during the CO_2_‐induced panic‐like behavior. Additionally, we used in vivo microdialysis to collect gliotransmitter released in the PAG while we activated astrocytes (Figure S13a). Optogenetic activation of PAG astrocytes induced a significant increase in extracellular ATP levels but not glutamate (Figure 6b,c), GABA, D/L‐serine, or 5‐HT (Figure S13b–d). Exposure to fox odor also induced an increasing trend of extracellular ATP, although that change was not statistically significant (Figure S13e,f). Based on these observations, we postulated that the increase in extracellular ATP, which was caused by a distinct Ca^2+^ increase evoked by aberrant astrocytic activation, is responsible for the panic‐related behaviors. To confirm that hypothesis, we inhibited gliotransmitter release from the activated astrocytes by using an Opto‐vTrap system that allows vesicular gliotransmitter release to be optically manipulated [45, 46]. An AAV expressing Opto‐vTrap under the GFAP promoter (AAV‐GFAP‐Opto‐vTrap‐mCherry) was introduced to the PAG areas of hGFAP‐ChR2 mice. After three weeks, PAG slices were prepared, and ATP levels were measured upon light stimulation (Figure 6d; Figure S13g). The inhibition of astrocyte vesicular gliotransmitter release significantly reduced extracellular ATP levels in the PAG area (Figure 6e; Figure S13h). Notably, in a behavioral analysis, Opto‐vTrap activation in PAG astrocytes almost completely abrogated the CO_2_‐induced panic‐related jumping behavior of mice (Figure 6f,g). The inhibition of vesicular gliotransmitter release also reduced defensive behavior in the presence of threatening stimuli (Figure 6h–k), but not during exposure to non‐threatening stimuli (Figure S13i,j). Meanwhile, it did not affect mouse behavior or normal locomotor activity during the OFT (Figure S13k–n). To directly test whether astrocyte‐derived ATP contributes to the regulation of defensive behavior, we pretreated the PAG with PPADS (100 mM), a purinergic receptor antagonist, via a guide cannula and then optogenetically stimulated PAG astrocytes (Figure 6l). PPADS‐treated mice showed a significant reduction in both the duration and amount of freezing behavior, despite optogenetic activation of PAG astrocytes (Figure 6m,n). These findings indicate that ATP released from PAG astrocytes is essential for modulating defensive responses in mice.

**FIGURE 7:**
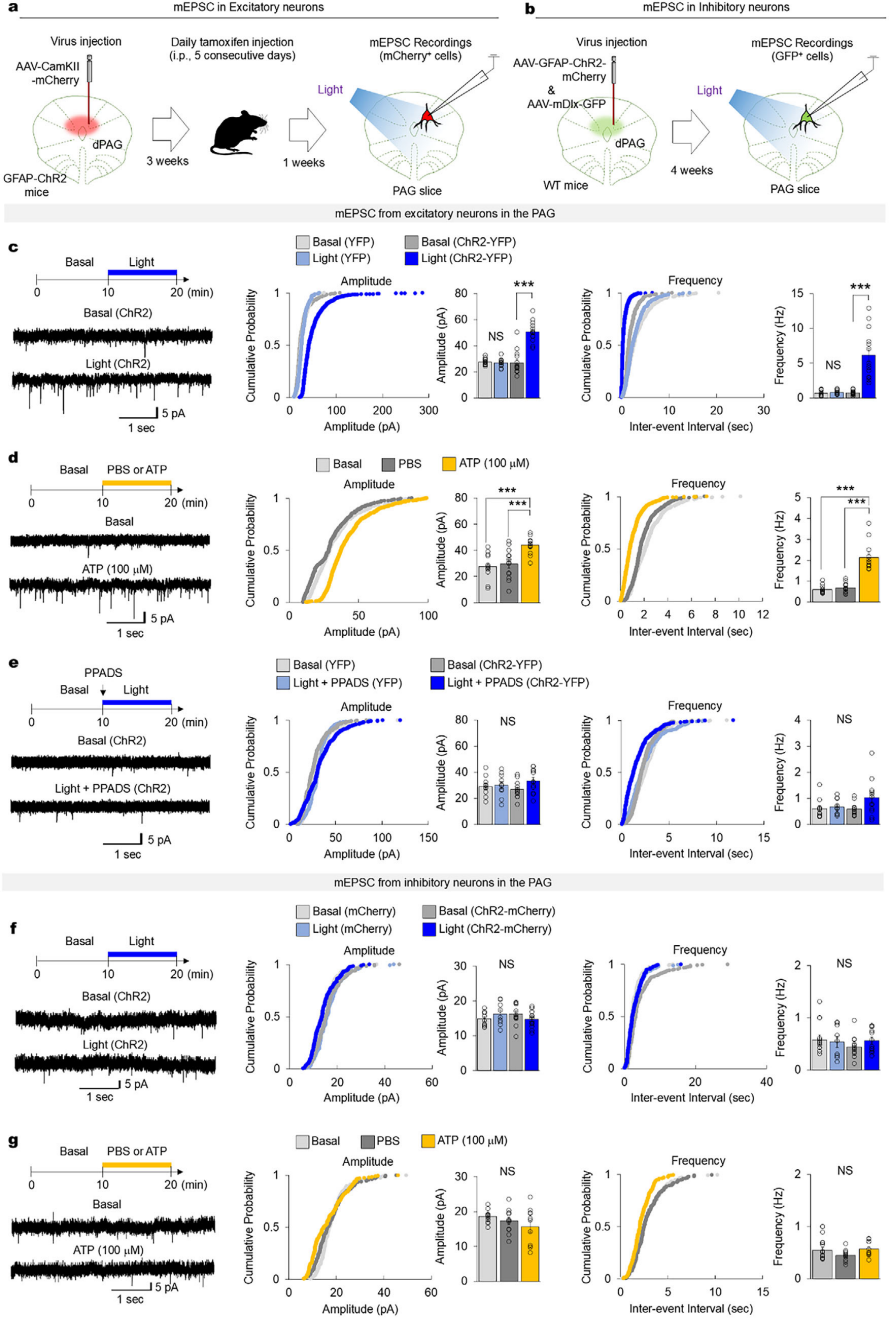
Activation of PAG astrocytes enhances excitatory synaptic activity. (a) Experimental setup showing CamKII‐mCherry injection into PAG and electrophysiological recordings of mCherry‐labeled PAG neurons. (b) Experimental setup showing co‐injection of GFAP‐ChR2‐mCherry and mDlx‐ChR2‐GFP into the PAG and electrophysiological recordings from GFP‐labeled PAG neurons. (c) mEPSC results upon optogenetic stimulation of PAG astrocytes, showing increased amplitude and frequency of mEPSCs with light stimulation of astrocytes (YFP: 12 cells from 5 mice; ChR2: 14 cells from 6 mice). (d) mEPSC results following bath application of 100 mM ATP, which similarly increased mEPSC amplitude and frequency as observed with optogenetic astrocyte stimulation (13 cells from 7 mice). (e) mEPSC results after pre‐treatment with PPADS, a purinergic receptor antagonist; PPADS pre‐treatment prevented the increase in mEPSC amplitude and frequency during optogenetic activation of PAG astrocytes (YFP: 10 cells from 4 mice; ChR2: 12 cells from 4 mice). (f) mEPSC recordings from PAG neurons during optogenetic stimulation of PAG astrocytes, showing increased mEPSC amplitude and frequency upon light activation (YFP: 9 cells from 3 mice; ChR2: 12 cells from 5 mice). (g) mEPSC recordings following bath application of 100 mM ATP, and comparison of mEPSC amplitude and frequency across basal, PBS‐, and ATP‐treated groups (11 cells from 5 mice). mEPSC data were compared using one‐way ANOVA followed by LSD post hoc analysis. ^***^ *p* < 0.001. Data are presented as the mean ± s.e.m. See Table S1 for detailed values and statistics.

To assess the influence of astrocytes on PAG neurons, we took electrophysiological recordings from acute brain slices. Initially, we injected AAV‐CamKII‐mCherry into GFAP‐ChR2 mice to record miniature excitatory post‐synaptic currents (mEPSC) from excitatory neurons (mCherry‐positive neurons) (Figure 7a). Three weeks post‐injection, we administered tamoxifen injections for five consecutive days before preparing PAG slices. To record mEPSCs, we specifically targeted mCherry‐expressing neurons in each PAG slice (Figure 7a). The results demonstrated that optogenetic light stimulation significantly increased both amplitude and frequency of mEPSCs in mCherry‐expressing PAG neurons (Figure 7c). To determine whether ATP application could mimic the synaptic activity changes observed with optogenetic astrocyte stimulation, we applied ATP in an extracellular ACSF buffer and recorded mEPSCs in PAG neurons. This ATP treatment similarly increased both the amplitude and frequency of mEPSCs (Figure 7d). Additionally, the application of pyridoxalphosphate‐6‐azophenyl‐2’,4’‐disulfonic acid (PPADS), a purinergic receptor antagonist, prevented light‐induced increases in mEPSC amplitude and frequency (Figure 7e), indicating that ATP receptor signaling mediates increases in mEPSCs induced by optogenetic astrocyte activation. In addition, we co‐injected GFAP‐ChR2‐mCherry and mDlx‐GFP into the PAG of wild‐type mice to record mEPSC from inhibitory neurons (GFP‐positive neurons) and performed identical electrophysiological analysis (Figure 7b). In contrast to the robust mEPSC changes observed in glutamatergic excitatory neurons (CamKII‐positive) (Figure 7c–e), optogenetic stimulation of astrocytes or bath application of ATP (100 mM) did not significantly alter glutamatergic synaptic inputs onto GABAergic neurons in the PAG (Figure 7f,g). This functional architecture of astrocyte‐dependent synaptic regulation was further supported by structural analyses of PAG astrocytes and surrounding synaptic puncta. Morphometric examination revealed that astrocytes contacted excitatory postsynaptic puncta (PSD95^+^) more frequently than inhibitory postsynaptic puncta (gephyrin^+^), whereas the overall densities of excitatory and inhibitory presynaptic puncta (vGlut2^+^ and VGAT^+^) did not differ significantly (Figure S14). These findings identify the postsynaptic targets of astrocyte–synapse interactions in the PAG and demonstrate that astrocyte activity preferentially modulates glutamatergic connectivity, thereby shaping the local excitatory‐inhibitory balance. Collectively, these results demonstrate that ATP released from PAG astrocytes modulates local excitatory circuitry, thereby driving defensive and CO_2_/fox odor‐induced panic‐like responses (Figure S15).

## 3 Discussion

The canonical subregions of the PAG area dictate appropriate defensive responses against threats; however, it was recently reported that PAG functions depend not only on its subregions, but also on a diverse array of cell types that span the PAG subregions [27, 46]. In addition to neurons, increasing evidence suggests that astrocytes play a role in regulating fear and anxiety behaviors, but no direct in vivo tests of astrocyte activity in response to threatening stimuli had been conducted. In this study, we are the first to report that PAG astrocytes respond in real time to threatening stimuli in vivo. These observations complement, but are distinct from, recent reports implicating PAG astrocytes in pain processing [47, 48]. Like those studies, our data support the broader principle that astrocyte activity can shape PAG output; however, our work focuses on ethologically relevant threat detection and panic‐like states rather than nociception, uses real‐time monitoring of astrocytic Ca^2^^+^ dynamics in awake behaving animals during multi‐sensory threats (fox odor) and CO_2_ exposure, and reveals that astrocyte activity biases the selection of defensive actions (freezing versus escape). Similar to previous reports, the salient threatening stimuli used in this study were intended to stimulate mouse sensory modalities: visual, olfactory, and mechanosensory. Notably, all the threat stimuli we used evoked PAG astrocyte activity and intracellular Ca^2+^ elevation, which occurred as the mice enacted appropriate defensive behaviors to avoid danger. Although we did not further explore the neural circuits involved in threat detection, previous works reported that the PAG receives information from brain areas that carry threatening sensory information, which enables mice to compute appropriate and efficient defensive responses [4, 7]. Therefore, it is conceivable that PAG astrocytes increase calcium activity in response to nearby neurons that are activated by incoming sensory threat information. Previous studies reported that glutamatergic neurons in the dorsal PAG elicit a calcium activity increase upon exposure to threat stimuli such as a looming object, predator, or exposure to CO_2_ [5, 7, 18], which might affect astrocyte Ca^2+^ activity in the midbrain PAG. Astrocytes express diverse receptors to sense neurotransmitters, neuropeptides, and even changes in extracellular pH, all of which can trigger intracellular Ca^2+^ elevation. In previous studies, midbrain VTA astrocytes regulated extracellular glutamate levels in the VTA through GLT‐1, which was reported to orchestrate approach and avoidance behaviors in mice [23]. It was also reported that ventral midbrain astrocytes exhibited calcium responses to glutamate and dopamine through D2 receptor signaling [49]. Therefore, it will be interesting to test whether those mechanisms also regulate PAG astrocyte activity.

In this study, we used pharmacological and optogenetic astrocyte manipulations and discovered that PAG astrocytes modulate defensive behavior in vivo. The use of L‐AAA as an astrotoxin might have introduced complications through the depletion of astrocytes; therefore, we complemented it by using astrocyte‐specific hPMCA2w/b or Opto‐vTrap. Despite this limitation in astrocyte inhibition, our results show that PAG astrocyte activity is necessary for mice to generate optimal defensive behaviors in threatening situations. Optogenetic activation of PAG astrocytes per se triggered mouse defensive behaviors. Notably, PAG astrocyte activity affected the selection of a defensive action against a threat. Specifically, optogenetic activation of PAG astrocytes biased mouse defensive responses toward the more passive freezing behavior rather than active defensive responses, regardless of the threat present or the availability of a safe zone. Between episodes of freezing behavior, we also observed episodes of aimless flight and avoidance upon PAG astrocyte stimulation. We speculate that PAG astrocyte affects the local circuitry and the functional columns of PAG through the release and/or uptake of neurotransmitters and neuromodulators [36, 50, 51]. It is also possible that astrocytes indirectly influence brain areas proximal to the PAG, such as the parabigeminal nucleus and superior colliculus, which mediate active defensive behaviors [7, 52]. In relation to the prior literature on PAG astrocytes in pain [47, 48], our findings converge on the idea that astrocyte‐to‐neuron signaling is a powerful gain controller of PAG computations. The key difference is that we demonstrate causal control over action selection in a defensive context and identify aberrant astrocytic overactivity as a driver of maladaptive, panic‐like responses, thereby extending the functional repertoire of PAG astrocytes beyond nociceptive modulation.

Notably, panicogenic stimulation with CO_2_ triggered aberrant PAG astrocyte Ca^2+^ overactivity. Although no single rodent model fully captures the complexity of human panic disorder, CO_2_ inhalation is a well‐validated interoceptive challenge that evokes high arousal, disorganized escape, and reduced safety seeking—core features that parallel human panic attacks. We used CO_2_ because it provides rapid and reproducible control of threat intensity while allowing precise monitoring of PAG astrocyte Ca^2^^+^ dynamics. To determine whether these effects generalize beyond interoceptive stimuli, we also tested predator odor (fox odor) and observed comparable astrocyte‐dependent panic‐like jumping and impaired escape. These convergent results across CO_2_ and fox odor indicate that PAG astrocyte dysregulation represents a shared mechanism underlying maladaptive defensive responses to diverse threats. Furthermore, pharmacological ablation or activity inhibition of PAG astrocytes in a panicogenic environment ameliorated the panic‐related jumping behaviors otherwise seen upon CO_2_ or fox odor exposure. These novel findings suggest that optimal PAG astrocyte activity is required to fine‐tune or select a defensive behavior, and therefore aberrant overactivation of PAG astrocytes could underlie the mechanisms for panic‐like behaviors that defy the goals of defensive behavior, i.e., rapid escape from the threatening environment and safety‐seeking. It is intriguing that optogenetically activating PAG astrocytes decreased mouse safety‐seeking behaviors in a threatening environment when optogenetic PAG astrocyte activation alone was sufficient to evoke defensive‐like behaviors. It is conceivable that PAG astrocyte activity could play a homeostatic role in modulating mouse defensive behaviors. Thus, physiological astrocyte activity is required for optimal defensive behavior, and aberrant astrocyte activity can lead to the dysregulation of safety‐seeking defensive behaviors in ways that resemble panic‐like behaviors.

As underlying mechanisms, we propose that changes in PAG astrocyte activity influence local neural circuits within the PAG and possibly neural projections connected to it through gliotransmitter release. In our study, PAG astrocyte activation evoked a significant increase in extracellular ATP levels that might cause changes in the neural activity of the PAG thereby leading to aberrant defensive responses in mice. A recent study showed that exposing mice to aversive stimuli induced astrocyte Ca^2+^ elevation and the subsequent release of ATP in the prefrontal cortex [50]. We also reported that ATP is released upon optogenetic stimulation of hippocampal astrocytes during mouse anxiety‐like behavior [36]. In this study, we found that the extracellular ATP level decreased upon Opto‐vTrap stimulation, which inhibits vesicular astrocyte gliotransmitter release. Although Opto‐vTrap significantly reduced the usual ATP increase in the PAG, it did not completely abrogate the increase in ATP levels upon astrocyte activation. We suspect that ATP is also released from astrocytes via other routes, such as channels, in addition to vesicular release [53]. Also, we cannot exclude the possibility that the ATP level increase is partially contributed by PAG neuronal activity, in addition to astrocytes.

Previous studies suggested that defensive responses are regulated by tonic inhibition of the PAG [16]; inhibitory signals to the PAG control the glutamatergic neurons responsible for active escape behaviors in mice. In this regard, it is possible that PAG astrocytes somehow disinhibit excitatory neurons in the dorsal PAG region, thereby assisting the activation of glutamatergic neurons that facilitate escape behavior upon exposure to a threat. Therefore, astrocyte ablation could impair the disinhibition of dorsal PAG glutamatergic neurons, which then results in the inefficient and reduced escape behaviors observed in this study. The release of ATP from PAG astrocyte activation might disrupt normal tonic inhibition in the PAG through A1 receptors associated with G‐protein‐gated inwardly rectifying potassium (GIRK) channels that hyperpolarize neurons [54]. It was also reported that astrocyte‐derived ATP inhibits neurons by modulating phasic and tonic inhibition in the neocortex and hippocampal local circuitry through purinergic A1 receptors on neurons that activate the GIRK channels [54, 55]. Those findings are in line with previous studies showing that optogenetic inhibition of glutamatergic neurons in the dorsal PAG region halted escape behaviors and produced freezing behaviors in response to a looming stimulus [7]. Similarly, defensive freezing and escape were controlled by a local circuit of GABAergic interneurons in the lateral and ventral subregions of the PAG area [4]. Furthermore, earlier studies showed that disrupting tonic inhibition in the PAG area using microinjections of GABA blockers evoked jumping and freezing behaviors in mice that mimicked panic‐like behaviors [16, 56]. Considering those prior reports, we speculate that overactivation of PAG astrocytes might inhibit the neural output of the PAG area by decreasing neuronal excitability via A1 receptors or GIRK channels, thereby producing aberrant passive freezing behavior and failure to efficiently escape a threatening situation, which is reminiscent of panic‐like behaviors.

We acknowledge that our study has a few limitations. First, although we identified ATP as a gliotransmitter involved in the PAG astrocyte modulation of defensive‐ and panic‐related behaviors, we have not elucidated the precise mechanisms (local or long‐range circuitry that regulates ATP increases in the PAG area) that are responsible for the defensive‐ and panic‐related behavioral changes we observed. Those mechanisms warrant future investigation. In addition, our study covered only the disruption of safety‐seeking and aimless jumping behaviors as behavioral read‐outs of panic‐like responses. Therefore, further study is needed to investigate the effects of aberrant PAG astrocyte overactivation on other panic‐related neurophysiological and autonomic responses. Furthermore, previous studies suggest the physiological diversity and morphological complexity of astrocytes, displaying diverse signaling, numerous branches and microdomains, and the formation of networks through gap junctions [22, 57, 58, 59]. Additionally, sex is also an important biological variable shaping defensive behaviors and PAG threat processing, with prior work demonstrating sex‐specific differences in fear, anxiety, panic‐like responses, and astrocyte signaling; thus, it is not known if PAG astrocytes play a role in the difference in defensive responses between male and females [60, 61, 62, 63]. Because our study used only male mice, our conclusions should be interpreted within the context of male physiology. Future studies including females‐along with estrous cycle staging‐will be required to determine whether PAG astrocyte Ca^2^^+^ activity and ATP‐dependent modulation of defensive behavior differ across sexes. Lastly, our study does not provide insight how CO_2_ inhalation increase aberrant calcium activity in PAG astrocytes. CO_2_ may directly influence neuronal membrane conductance or neuronal firing [64, 65]. Previous studies also reported that CO_2_ reduces brain pH [66, 67] and elicit astrocyte calcium signal in the brainstem [68, 69].

In conclusion, PAG astrocytes respond to threatening stimuli by elevating calcium activity, which modulates mouse defensive behavior. Therefore, aberrant PAG astrocyte activation in mice induces abnormal defensive responses and uncontrollable panic‐like behaviors that defy the goal‐directed defensive response to seek safety, avoid threats, and maximize the chance of survival. Our findings suggest that aberrant PAG astrocyte activity with distinct Ca^2+^ elevation and increased ATP release might underlie the mechanisms of panic‐related behaviors.

## 4 Materials and Methods

### 4.1 Animals

Six‐ to 16‐week‐old male CB7BL/6J and GFAP‐CreERT2 mice were used in the experiments. The GFAP‐CreERT2 mice were obtained from the laboratory of Dr. Frank Kirchhoff (Max Plank Institute, Munich, Germany). For the optogenetic modulation of astrocytic activity, floxed‐ChR2(H134R)‐EYFP (#12569, Jackson Laboratory, Bar Harbor, ME, USA) mice were crossed with GFAP‐CreERT2 mice (hGFAP‐ChR2). The resulting hGFAP‐ChR2 mice were intraperitoneally treated with 100 mg/g of tamoxifen (#13258, Cayman Chemical, Ann Arbor, MI, USA) for five consecutive days starting when they were 6 weeks old. For the control group, vehicle (90% corn oil and 10% ethanol) was administered at the same volume as the tamoxifen. All experiments were conducted within 2 months of the first tamoxifen injection. All control mice were from the same line and genotype as experimental animals, but they have all been vehicle injected. The animals were housed and maintained in a controlled environment kept at 22°C–24°C and 55% humidity with 12‐h light/dark cycles, and they were fed with regular rodent chow and tap water ad libitum. All animal experiments were performed according to the guidelines of Seoul National University Institutional Animal Care and Use Committee (SNU‐241206‐1‐1).

### 4.2 Stereotaxic Surgeries

Animals that were subjected to the implantation of probes and optic fibers or microinjections of a virus or chemical were anesthetized with 1.5%–2.0% isoflurane and secured in a stereotaxic apparatus (Stoelting Co., Wood Dale, IL, USA). For the in vivo calcium activity recording experiments, holes the size of the injection needle were drilled unilaterally into the skull ±0.40 mm from the midline and at the level of lambda suture. Using a syringe pump (Stoelting Co.), a 10‐mL Hamilton syringe (Hamilton Co., Reno, NV, USA) delivered 0.8 mL of pZac2.1‐GfaABC1D‐lck‐GCaMP6f (#52924 plasmid, 52924‐AAV5, Addgene, Watertown, MA, USA) to the dPAG (anteroposterior (AP): 0.00 mm, mediolateral (ML): ±0.40 mm, dorsoventral (DV) ‐2.50 mm from the lambda). Virus was injected at a constant rate of 0.2 mL/min, and the needle was left in place for 10 min after the injection to minimize the upward flow of the viral solution while the needle was slowly removed. This was followed by TCSPC probe implantation with the same coordinates but with DV −2.30 mm superior to the viral injection site. The implantation was fixed with zinc polycarboxylate dental cement. A similar protocol was used when injecting AAV‐hSyn‐oChIEF‐tdTomato, AAV‐GFAP‐ChR2‐mCherry, AAV‐GFAP‐ChR2‐GFP, AAV‐CamKII‐ChR2‐GFP, AAV‐GFAP‐jRGECO1a‐mCherry (∼10^12^ GC/mL, IBS Virus Faculty, Institute for Basic Science, Daejeon, Rep. of Korea), pAAV‐mDlx‐ChR2‐mCherry‐Fishell‐3 (#83898‐AAV9, Addgene), or pAAV‐mDlx‐GFP‐Fishell‐1 (#83900‐AAV9, Addgene) into the dPAG areas of hGFAP‐ChR2 mice for neuronal manipulation. After 7 days, 200‐mm optic fiber (FP200URT; Thorlabs Inc., Newton, NJ, USA) with a 1.25 mm ceramic ferule (CFLC230‐10; Thorlabs Inc.) was unilaterally or bilaterally implanted in the mice at the same coordinates, except the depth, which was DV −2.30 mm superior to the viral injection site. The mice were given another 7 days for virus expression and recovery. A similar protocol was applied when injecting AAVDJ‐GFAP‐Opto‐vTrap (IBS, Daejon, South Korea) into wild‐type and hGFAP‐ChR2 mice. Similarly, for melanopsin experiment, wild type mice were injected with 0.5 mL pAAV‐GFAP104‐melanopsin‐mCherry (#122630, Addgene).

To inject L‐AAA or PBS into the dPAG area (AP 0.00 mm, ML ±0.40 mm, DV −2.50 mm from the lambda), the mice received 0.8 mL of either 0.1 M PBS or 20 mg/mL L‐AAA (A7275, Sigma‐Aldrich, Burlington, MA, USA) from a syringe pump with a constant rate of 0.2 mL/ min. The needle was left in place for 10 min after the injection. After we removed the needle, we applied 3 to 4 sutures to close the skin of the head. The mice were allowed to recover for 2 to 3 days before they were subjected to behavioral or western blot experiments. For hPMCA2w/b experiment, mice received 0.5 mLof either GFAP‐mCherry or pZac2.1‐GfaABC1D‐mCherry‐hPMCA2w/b (#111568‐AAV5, Addgene). The mice were allowed to recover for 5 weeks prior to behavioral and histology experiments. For in vivo fiber photometry recording of ATP, glutamate, and GABA, mice were injected unilaterally in dPAG with 0.5 mL AAV‐hSyn‐GRAB_ATP1.0 (#IBS‐VF‐2023, IBS), pAAV‐hSyn‐iGluSnFr‐WPRE.SV40 (#98929‐AAV5, Addgene), and pAAV‐hSyn‐iGABASnFR (#112159‐AAV1, Addgene). The mice were allowed to recover for 5 weeks prior to behavioral experiments.

For optic fiber implantation, a 200‐µm mono fiber‐optic cannula with a 1.25 mm ceramic ferrule (Thorlabs Inc.) was implanted into hGFAP‐ChR2 mice and fixed with zinc polycarboxylate dental cement bilaterally (angle 26°, AP 0.00 mm, ML ±1.40 mm, DV −2.40 mm from the lambda) or unilaterally (AP 0.00 mm, ML ±0.40 mm, DV −2.30 mm from the lambda) to the dPAG area. The mice were given 7 days to recover before the behavioral experiments.

For microdialysis guide cannula implantation, a CMA7 guide cannula (C315MN; CMA Microdialysis, CMAP000137, Kista, Sweden) covered by a dummy (angle 26°, AP 0.00 mm, ML ±1.40 mm, DV −2.40 mm from the lambda) and a mono fiber‐optic cannula (AP 0.00 mm, ML ±0.40 mm, DV −2.30 mm from lambda) were implanted in hGFAP‐ChR2 mice to simultaneously stimulate dPAG astrocytes and perform in vivo microdialysis. The implantations were secured with zinc polycarboxylate dental cement. The mice were given at least 3 days to recover before the microdialysis experiment was performed. Animals with mistargeted implantations or virus expression were excluded from the analyses.

### 4.3 Time‐Correlated Single‐Photon Counting

Two weeks after virus expression of GCaMP6f and TCSPC probe implantation, a single wavelength TCSPC system (Becker & Hickl GmbH, Berlin, Germany) was used for in vivo astrocytic calcium signal acquisition. A 488‐nm laser (BDL‐488‐SMN; Becker & Hickl GmbH) was used to excite a GCaMP6f signal at 20 MHz through a single‐mode fiber (F200‐Hybrid‐FC‐COLL‐OPTC; Becker & Hickl GmbH) connected to a multi‐mode probe (F05‐MM‐FP‐OPTH; Becker & Hickl GmbH). The laser power was set to approximately 0.1 mW, as measured at the tip of the optic probe using an optical power sensor (PM100D‐S121C; Thorlabs Inc.). Photons emitted from the brain tissue traversed the multi‐mode fiber and were collected using a 16‐channel multi‐wavelength photodetector (PML‐SPEC‐1‐C; Becker & Hickl GmbH) controlled by a detector controller (DCC‐100; Becker & Hickl GmbH). The number of photons was counted at 0.1 Hz. The original data were smoothed with a Gaussian filter and converted into Excel file using customized MATLAB codes (MathWorks). The data acquired are presented as z‐scores. The detected photon counts are denoted as x, and the mean and standard deviation of the photon count over the entire experimental session are denoted as µ and σ, respectively. The z‐scored photon counts were computed as (x−µ)/σ.

### 4.4 Two‐Photon Microscope Imaging

#### 4.4.1 Surgeries

We used 8‐ to 10‐week‐old C57BL/6J mice and injected AAV5‐gfaABC1D‐GCaMP6f into the PAG. For optical access to the PAG, we performed gradient refractive index (GRIN) lens (Inscopix, Palo Alto, CA, USA) implantation. The anesthetized mice were first mounted in a stereotaxic frame. After removing the scalp, the skull was leveled with Bregma and Lambda. Using a dental drill and carbide burrs (#19007‐09, Fine Science Tools, Foster City, CA, USA), 0.2 mm right to Lambda were slightly thinned. The 1‐mm‐diameter and 5‐mm‐long GRIN lens were implanted above the dorsal PAG. The lens was fixed to the skull with tissue adhesive (3 M, Vetbond, St. Paul, MN, USA) and covered with dental cement. The exposed end of the GRIN lens was protected with transparent Kwik‐seal glue and animals were returned to a cage. Two weeks later, a customized stainless‐steel head ring with an outer diameter of 11 mm and inner diameter of 9 mm was then placed around the GRIN lens and fixed by adding more cement.

#### 4.4.2 Two‐Photon Imaging

The PAG astrocytes were imaged through a two‐photon excitation laser scanning microscope (Olympus, FVMPE‐RS, Tokyo, Japan). The two‐photon microscope was equipped with two GaAsP photomultiplier tubes, a galvanometer/resonant scanner, and a Ti:Sapphire laser (Spectra‐Physics, Mai‐Tai DeepSee, Clarksburg, NJ, USA). A 25×1.00 numerical aperture water immersion objective (Olympus, XLPLN25XSVMP2) was used for imaging, with wavelengths of 900 nm and 1030 nm for GCaMP6f imaging, respectively. The emission fluorescence signal was collected after passing through a filter cube (Olympus, FV30‐FGR), consisting of a 570‐nm low‐pass dichroic mirror and two‐emission filters (i.e., 495–540‐nm and 575–645‐nm bandpass filters). The mice were habituated to the imaging setup for 5 days by daily handling and insertion into a microscope platform with their head fixed under the microscope. On imaging day, mice were first mounted on the two‐photon microscope platform. Calcium imaging was performed for 90 s and images of 384 µm×384 µm areas were acquired at 30 Hz. For the first 30 s, baseline calcium activity was recorded, followed by exposure to fox odor for 2 s and recording calcium activity during interaction between the two mice for the remaining 60 s.

#### 4.4.3 Image Analysis

Time‐lapse images were first corrected for motion artifacts from breathing and cardiac function using NoRMCorre software. The signal was enhanced by averaging every three frames. To extract the calcium signal from the resulting 10‐Hz time‐lapse images, we used CaImAn software with a constrained non‐negative matrix factorization algorithm. From the calcium footprints detected by CaImAn, footprints detected as separate ROIs were manually integrated. For each ROI, raw fluorescence *F*(*t*)was detrended for slow bleaching and converted to Δ*F*/*F*_0_ = (*F*(*t*) − *F*_0_)/*F*_0_, where *F*_0_was the median fluorescence during the pre‐odor baseline (−2 to 0 s). Traces were lightly smoothed (3‐point moving average). We then z‐scored Δ*F*/*F*_0_to the baseline distribution (mean and SD computed over −2 to 0 s). An ROI was labeled activated if the z‐scored signal exceeded 3 SD above baseline for ≥ 1 s within 0–7 s after odor onset. For each animal, the activation rate was computed as # activated ROIs / #total ROIs; group values are reported as the mean across animals (± s.e.m.).

### 4.5 Behavioral Experiments

All mice were placed in the test room and left acclimatized there for at least 30 min before the behavioral experiments were performed. All the experiments were conducted using one mouse at a time, and the equipment was cleaned with 70% EtOH between animals to remove olfactory cues from previous mice.

#### 4.5.1 Threat and Non‐Threat Assays

Fox odor exposure. TMT (2199185‐5G, Sigma‐Aldrich), a component of fox feces, was used as one of the innate threat stimuli. On the day of the experiment, a cotton swab for each animal subject was dipped in 100 µL of TMT (1:10) and put in a 15 mL conical tube that was tightly closed until the exposure experiment began. The day before the exposure experiment, the mice were placed in an open all‐white arena (40 cm×40 cm×40 cm) and allowed to habituate there for 7 min. Pre‐exposure data were recorded during this habituation session. On the exposure day, the conical tube containing the cotton swab with fox odor was opened and put inside the open arena, and then the mice were brought to the test area individually. For the c‐Fos detection experiment, the mice were sacrificed 1 h after exposure to the fox odor. For TCSPC Ca^2+^ recording, pre‐exposure data were obtained from the similar method above. During fox odor exposure day, mouse was individually brought to the test area and was placed to the open field. Five seconds after Ca^2+^ recording started (baseline recording), conical tube containing the cotton swab with fox odor was opened and put inside the open arena. Calcium activity was recorded from baseline through response to fox odor. Mouse behaviors were recorded and quantified with an automated tracking system, SMART 3‐0 (Panlab Harvard Apparatus, Holliston, MA, USA).

#### 4.5.2 RC Car Attack

For the in vivo calcium activity recording, the mice were individually placed in an open rectangular arena (length 85 cm×height 40 cm×width 40 cm) and allowed to habituate for 5 min. The Ca^2+^ activity recorded during the habituation session was used as the baseline. After 5 min of habituation, a red RC car (length 20 cm×5 cm height×width 8 cm) was placed in the corner opposite the mouse. The RC car was controlled to make a forward movement, mimicking an attack, while the mouse approached or faced it. Each session consisted of at least three trials. Mouse behaviors were recorded and quantified with an automated tracking system. For the c‐Fos detection experiment, the mice were sacrificed 1 h after a 10‐min session of the RC car attack assay.

#### 4.5.3 Hand Attack

Each mouse was placed in the open arena (40 cm×40 cm×40 cm) for 5 min prior to the experiment. For the TCSPC recordings, the Ca^2+^ activity recorded in the habituation period was used as the baseline. After habituation, the experimenter applied the stimulus by quickly touching the back of the mouse with their hand. The hand attack was applied every 30 s for 5 min. Hand attacks are an innate threat that are not present in the open arena and cannot be investigated or approached by the mouse, unlike the RC car. Instead, the hand randomly attacked the mouse from above, mimicking a previously reported looming stimulus used as a predator replica. The speed of the mice escaping from the hand attack was detected using an automated tracking system. For the c‐Fos detection experiment, the mice were sacrificed 1 h after a 5‐min session of the hand attack assay.

#### 4.5.4 Carbon Dioxide Exposure

The mice were placed in a transparent box (height 30 cm×width 15 cm×depth 15 cm) with an open top and habituated there for 5 min. After habituation, the mice were exposed to either air or CO_2_ delivered via 3‐s infusion of pure CO_2_. The number of jumps and duration of freezing and exploration were recorded and quantified using SMART 3‐0 software.

#### 4.5.5 Acute Pain

A formalin injection in the paw is a widely used model of acute pain [48] and was used in this study as another aversive stimulus to observe whether it induces c‐Fos expression in the dPAG. Paraformaldehyde in 0.1 M PBS (4%, 10 mL) was injected into the plantar surface of the left hind paw of each mouse. A few minutes after injection, the injected paw was red and swollen, and the mouse held and licked it. One hour after the paw injection, the mice were sacrificed for c‐Fos immunostaining.

#### 4.5.6 Fear Conditioning

This experiment was conducted in fear‐conditioning boxes that consisted of one clear plexiglass wall, three aluminum walls, and a stainless‐steel grid as a floor (28×28×28 cm, Coulbourn Instruments, Holliston, MA, USA). For the conditioning (training day), the mice were placed in the chamber for 2 min and exposed to a series (2 pairing, 110‐s intertrial interval) of cue presentations (2.9 kHz pure tone, 20 s duration) that co‐terminated with a mild foot shock (0.4 mA, 2 s duration). Thirty seconds after the last set of cues, the mice were placed back in their home cages. For the contextual fear conditioning test, the mice were placed in the original conditioning chamber 24 h after conditioning, and freezing behavior was measured for 5 min. For the auditory‐cued fear conditioning, the mice were placed in a test chamber with modified contextual elements (floor and walls). Freezing behavior was measured for 150 s to establish a baseline, and that was followed by a 150‐s cue (2.9 kHz pure tone) exposure period. The grid and waste tray were cleaned with 70% ethanol between runs. Mouse behaviors were recorded using a digital video camera mounted above the chamber and scored automatically for the freezing response using FREEZEFRAME software (AM1‐FF04, Lafayette Instrument, Lafayette, IN, USA). The data are presented as the percentage of time in the freezing response.

#### 4.5.7 Home Cage and Sucrose Exposure

For the dPAG c‐Fos expression experiment, mice that were not exposed to any other stimuli but their normal home cages were used as the control group, and they were sacrificed together with the mice exposed to various stimuli. For the sucrose stimulus, the mice were prevented from drinking water for 12 h, and then a 10% sucrose solution was provided in their normal drinking bottles. The mice were allowed to access the 10% sucrose solution for 30 min and were sacrificed 1 h after the onset of sucrose exposure.

#### 4.5.8 Female Mouse and Novel Object Exposure

For the TCSPC recording, the mice were habituated to an open arena (40 cm×40 cm×40 cm) for 5 min prior to the introduction of a female mouse or novel object. For female mouse exposure, the female mice were habituated to a wired cup (height 16 cm, diameter 8 cm) with a white lid for at least 5 min. After the 5 min habituation period, the female mouse in the wired cup was placed in the open arena. The subject mice were thus exposed to the female mouse and allowed to explore and interact with her for 7 min. For the novel object exposure experiment, a cartoon figure about the same size as the subject mice (height of 4 cm) was affixed in the open arena using adhesive tape. The subject mice were exposed to the novel object and allowed to explore and interact with it for 7 min. Mouse behaviors were recorded, and the duration and number of times the mice sniffed the stimuli were quantified using SMART 3‐0. For the c‐Fos expression experiment, the subject mice were sacrificed 1 h after exposure to the stimulus.

#### 4.5.9 Optogenetic Stimulation in Open Arena

Seven days after recovery from stereotaxic implantation of an optic fiber into the dPAG area, hGFAP‐ChR2 or control mice, and GFAP104‐melanopsin‐injected mice were placed in the test room 30 min before the experiment was started. Mice with optic fibers connected to a laser were placed in an open field arena (40 cm×40 cm×40 cm) and habituated for 5 min. The last 2 min of the habituation was considered to be the first light‐off epoch and was followed by a 2‐min light‐on epoch consisting of 3 mW of continuous stimulation. This cycle was repeated to complete a 10‐min session (2 min off—2 min on—2 min off—2 min on—2 min off). A freezing response was defined as drastic immobility in an inflexible posture aside from breathing. A fleeing response was characterized as jumping and rapid changes of direction. A retreat response was defined as backward movement. The tail rattling response was characterized by stiffness followed by a fast waving movement of the tail. The behavioral procedures were identical to those used in the PPADS pretreatment experiments. Prior to behavioral testing, a guide cannula was implanted into the PAG of hGFAP‐ChR2 mice. On the experimental day (2 weeks after implantation), 1 mL of PPADS (100 mM) was administered through the guide cannula 30 min before behavioral testing (injection rate, 0.1 mL/min), after which the behavioral assessments combined with optogenetic stimulation were performed using the same protocol as described above. Animal behaviors were recorded and manually quantified using SMART 3‐0 by an experimenter blinded of the experimental conditions of the mice.

#### 4.5.10 Available Shelter and Optogenetic Stimulation

hGFAP‐ChR2 and control mice were habituated to an open field arena (40 cm×40 cm×40 cm) with a triangular shelter that was white on the outside and black on the inside (length 18 cm×height 12 cm×width 15 cm) for 5 min/day for 3 days. Those habituated mice approached the shelter when the experimenter placed it in the open arena. A successful escape was scored when the mice reached the shelter within 5 s of its availability. On the stimulation day, constant light stimulation was delivered when mice approached and before they reached the shelter. Light stimulation was delivered for 10 s and was turned off when mice reached the shelter. At least two trials were performed for each mouse, and each trial lasted for 30 s. Successful escape bouts, latency to reach the shelter, freezing, fleeing, retreating, and tail rattling were quantified manually in SMART 3‐0 Panlab software by an experimenter blinded to the conditions of the mice.

#### 4.5.11 Available Shelter and Threat with Optogenetic Stimulation

On the experiment day, mice were habituated to an open field arena (length 85 cm×height 40 cm×width 40 cm) containing a shelter that was gray on the outside and black on the inside, with a dark gray lid (20 cm×20 cm×20 cm) for 10 min, and then an RC car (length 20 cm×5 cm height x width 8 cm) was introduced at the side of the arena opposite the shelter. The mice were allowed to explore the area with the RC car for 8 min. After 8 min, any mouse who approached the RC car was randomly attacked to evoke an immediate escape to the shelter. A successful escape was scored when a mouse reached the shelter within 5 s of the onset of the RC car attack. During light‐on epochs, constant light stimulation (3 mW) was delivered when the mice approached the RC car. Successful escapes, latency to reach the shelter, and defensive‐like behaviors were quantified by an experimenter blinded to the condition of the experimental groups.

#### 4.5.12 Simultaneous Stimulation of Neurons and Astrocytes

hGFAP‐ChR2 mice were injected with hSyn‐oChIEF‐tdTomato or control virus. After 7 days, optic fibers were implanted into the dPAG. The mice were allowed to recover for another 7 days. After the recovery period, the mice were subjected to an escape experiment in an all‐white rectangular open arena (length 85 cm×height 40 cm×width 40 cm) containing a shelter that was gray on the outside and black on the inside with a dark gray lid (20 cm×20 cm×20 cm) and a stimulation zone containing a novel mouse‐sized object affixed at the center to enrich the open arena and induce the mice to explore. The hSyn‐oChIEF‐tdTomato‐injected hGFAP‐ChR2 mice were placed in the arena and given 10 min to freely explore the environment. After the 10 min habituation session, blue light stimulation (∼3 mW, 40 Hz) for 10 ms, constant light, or no light, was delivered when the mice entered the stimulation zone. The light was turned off when the mice left the stimulation zone. A successful escape or flight response was scored when a mouse reached the shelter within 5 s of the onset of light stimulation. The experiment sessions lasted 30–40 min per mouse with at least 5 trials for each light condition. Twenty‐four hours after the first escape experiment, the same hGFAP‐ChR2 mice were injected with 100 mg/g of tamoxifen or vehicle (control group) for 5 days to express ChR2‐eYFP on the astrocytes. Two days after the last injection of tamoxifen, the same mice, now expressing both hSyn‐oChIEF and hGFAP‐ChR2, were subjected to the same escape experiment using the same procedures. Light stimulation (∼3 mW, 40 Hz for 10 ms or continuous) lasted for 1 min and resumed after 30 s if the mice did not leave the stimulation zone. To distinguish whether the observed behavioral effects were mediated by excitatory or inhibitory neuronal populations, AAV‐CamKII‐ChR2‐GFP (targeting excitatory neurons) or AAV‐mDlx‐ChR2‐GFP (targeting inhibitory neurons) was injected into GFAP‐ChR2‐mCherry‐expressing wild‐type mice. The behavioral testing procedures and optogenetic stimulation parameters were identical to those used in the preceding experiments. The sessions for this second escape experiment also lasted 30–40 min per mouse and contained at least 5 trials for each light condition. The percentage of successful escapes, latency to reach the shelter, and observed defensive‐like behaviors were recorded and quantified in SMART 3‐0 software by an experimenter blinded to the experimental conditions.

#### 4.5.13 Neuronal Stimulation and Astrocyte Ablation

hSyn‐oChIEF‐tdTomato was injected into the dPAG areas of C57BL/6 mice, and after 14 days of virus expression, 0.1 M PBS or 20 µg/µL of L‐AAA was injected into the same site, followed by the unilateral implantation of an optic fiber. The mice were given 2–3 days to recover before they were subjected to the escape experiment. On the experiment day, the mice were placed in the rectangular arena (length 85 cm×height 40 cm×width 40 cm) containing a shelter that was gray on the outside and black on the inside with a dark gray lid (20 cm×20 cm×20 cm) and a stimulation zone containing a novel mouse‐sized object affixed at the center to enrich the open arena and induce the mice to explore. The mice were given 10 min to habituate and explore the environment. The L‐AAA‐ or PBS‐injected mice with hSyn‐oChIEF‐tdTomato were given light stimulation (∼3 mW, 40 Hz, for 10 ms or constant) or not randomly when they entered the stimulation zone. The light was turned off when the mice moved outside the stimulation zone. A successful escape or flight response was scored when the mice reached the shelter within 5 s of the onset of light stimulation. The experimental sessions lasted 30–40 min per mouse with at least five trials for each light condition. The percentage of successful escapes, latency to reach the shelter, and observed defensive‐like behaviors were recorded and quantified in SMART 3‐0 software by an experimenter blinded to the experimental conditions.

#### 4.5.14 Opto‐vTrap Stimulation

After a recovery period, wild‐type mice or hGFAP‐ChR2 mice injected with GFAP‐Opto‐vTrap were subjected to behavioral experiments. The Opto‐vTrap‐injected mice were placed in the test room 30 min before the experiment started. Mice with optic fibers connected to a laser were placed in an open field arena (40 cm×40 cm×40 cm) for a 5‐min session. The first 2 min of the session was considered the first light‐off epoch, and it was followed by a 2‐min light‐on epoch consisting of 3 mW of constant blue light. For the last min, the light was turned off. The behaviors of the animals were recorded and automatically quantified using SMART 3‐0, though freezing behavior was quantified manually.

#### 4.5.15 Hot Plate and CO_2_ Assay with Escape Route

The hot plate assay was done using a metallic plate (MSH‐20A, Daihan Scientific, Daegu, South Korea) heated to about 40°C or left at room temperature. A transparent box (18 cm×18 cm×30 cm) with an open top and bottom was placed on top of the heated plate. A rope, attached from the sides of the transparent box, was present as an escape route that the mouse could climb to avoid the heat. The mice were habituated in the set‐up one day prior to the experiment day. On the experiment day, each mouse was placed in the set‐up with the plate either heated or at room temperature for a 15 min session. To avoid the heated plate, the mice climbed the rope and then went back to the heated floor. The number of climbs and average latency of climbing since touching the hot plate were quantified.

For all the following behavioral experiments, the animals were placed in the test room and left to acclimatize for at least 30 min before the experiment began. The behavioral experiments were monitored and recorded using a computerized tracking system (SMART 3‐0). All the behavioral experiments were analyzed automatically or manually by experimenters blinded to the experimental conditions.

#### 4.5.16 Open Field Test

The OFT apparatus is an all‐white 40×40 cm square arena surrounded by 40 cm high walls. The mice were individually placed in the center of the arena, and an automatic system monitored their locomotion activity for 6 min. The distance traveled and time spent in each area were analyzed using an automated video tracking system. The arena was cleaned with 70% ethanol after each use to eliminate any olfactory cues from the previously tested mouse.

#### 4.5.17 Tail Suspension Test and Forced Swim Test

To observe whether the experimental treatments caused changes in depressive‐like behaviors in the mice, we conducted the TST and FST. The TST was conducted in an all‐white rectangular chamber (height 30 cm, width 15 cm) by wrapping adhesive tape around each mouse's tail 1 cm from the tip and tying it to a hook in the chamber. In that way, each mouse was suspended for 6 min, and the immobility time was automatically measured. The chamber was cleaned with 70% ethanol after each use to eliminate any olfactory cues from the previously tested mouse. The FST was conducted by placing each mouse individually in a transparent cylinder filled with water (23°C–25°C; depth 18 cm, height 30 cm) for 6 min. Immobility in the FST was defined as a state in which the mouse made only the movements necessary to keep its head above the water. The data acquired were automatically analyzed using a video tracking system.

#### 4.5.18 Elevated Plus Maze

To observe whether the experimental treatments caused changes in anxiety‐like behaviors in mice, the EPM was performed. It was conducted in an all‐white apparatus that consisted of two open arms and two closed arms (width 5 cm x length 30 cm), all elevated 50 cm above the floor. The mice were placed individually in the center of the maze facing an open arm and allowed to explore freely for 6 min. The distance traveled and time spent in each arm were analyzed using a video tracking system. The maze was cleaned with 70% ethanol after each test to prevent olfactory influence from the previously tested mouse.

#### 4.5.19 Real‐Time Place Preference and Conditioned Place Preference

The RTPP and CPP were performed in a rectangular apparatus consisting of two side chambers (25 × 25 × 30 cm each) with black stripes and gray circles on the walls, connected by a central corridor (10 × 10 cm). In the RTPP experiment, the mice were habituated (pretest, days 1–3) and allowed to freely explore the entire apparatus for 10 min/day for 3 days. On the test day (day 4a), each mouse was placed in the central corridor and given light stimulation (∼3 mW, constant) only when they were within the designated light‐paired chamber. The light was turned off when they remained in the chamber for more than 30 sec, and it was turned on again after 10 s if they failed to leave the light‐paired chamber. This RTTP test lasted for 10 min. For the post‐test session (day 4b), the mice were given another 10 min to freely explore both chambers without any light stimulation pairing. The CPP used another set of mice who were habituated (pretest, days 1–3) for 10 min/day for 3 days and allowed to freely explore the entire apparatus. On the conditioning day (day 4), the mice were placed in the light‐off‐paired chamber for 12 min while the corridor to the other chamber was blocked. After 12 min, the corridor was opened, and each mouse was gently led to the other chamber. Once the mice reached the second chamber, the corridor was blocked. The mice were given 2 min to explore the chamber, and then light stimulation (∼3 mW, constant) was delivered for 5 min. The light was then turned off, and the mice were kept in that chamber until 12 min had passed. On the test day (day 5), the mice were placed in the center corridor and allowed to freely explore the apparatus for 10 min. A preference for one chamber over the other was calculated as the percentage of time spent in the stimulation chamber divided by the total time spent in both chambers. All behaviors were recorded using an automated video tracking system.

### 4.6 Slice Electrophysiology

Transverse acute slices of the PAG (300 mm) were prepared. Briefly, mice were anesthetized with isoflurane and decapitated. The brain was rapidly removed and placed in ice‐cold oxygenated (95% O_2_ and 5% CO_2_), low‐Ca^2+^/high‐Mg^2+^ dissection buffer containing 5 mM KCl, 1.23 mM NaH_2_PO_4_, 26 mM NaHCO_3_, 10 mM dextrose, 0.5 mM CaCl_2_, 10 mM MgCl_2_, and 212.7 mM sucrose. Slices were transferred to a holding chamber in an incubator containing oxygenated (95% O_2_ and 5% CO_2_) artificial cerebrospinal fluid (ACSF) composed of 124 mM NaCl, 5 mM KCl, 1.23 mM NaH_2_PO_4_, 26 mM NaHCO_3_, 10 mM dextrose, 2.5 mM CaCl_2_, and 1.5 mM MgCl_2_ at 28°C–30°C for at least 1 h before recording. After recovery, slices were transferred to the recording chamber where they were perfused continuously with ACSF gassed with 95% O_2_/5% CO_2_ at a flow rate of 2 mL/min. Slices were equilibrated for 5 min prior to recordings and all the experiments were performed at 30°C–32°C. Recordings were obtained using a Multiclamp 700B amplifier (Molecular Devices, Sunnyvale, CA, USA) under visual control with differential interference contrast illumination on an upright microscope (BX51WI; Olympus). Patch pipettes (4–6 MΩ) were filled with 130 mM Cs‐MeSO4, 0.5 mM EGTA, 5 mM TEA‐Cl, 8 mM NaCl, 10 mM HEPES, 1 mM QX‐314, 4 mM ATP‐Mg, 0.4 mM GTP‐Na, and 10 mM phosphocreatine‐Na2 to record mEPSC (pH 7.4 and 280–290 mOsm). Only cells with access resistance <20 MΩ and input resistance >100 MΩ were studied. The extracellular recording solution consisted of ACSF supplemented with picrotoxin (100 mM) and tetrodotoxin (1 mM) for mEPSC.

The mEPSC experiment was conducted using a 20‐min scheme (10 min without light for basal, 10 min with light stimulation) for each cell (CamKII‐mCherry‐positive cells for excitatory neurons; mDlx‐GFP‐positive cells for inhibitory neurons). Basal mEPSC was recorded for the first 10 min without light stimulation (0–10 min). Then, 473‐nm light (continuous) was delivered to the PAG slice through an optic fiber in a patch pipette for 10 min (11–20 min). An identical experimental procedure (light‐off for 10 min and light‐on for 10 min) was used for mEPSC recording with optogenetic light stimulation in the presence of PPADS (Tocris Bioscience, Bristol, UK). The extracellular recording solution consisted of ACSF supplemented with picrotoxin (100 µM), tetrodotoxin (1 mM) and PPADS (100 µM).

For mEPSC recording while changing extracellular ATP concentration, ACSF solution with 100 µM of ATP was prepared. Basal mEPSC was recorded for 10 min without ATP in ACSF buffer, and then the buffer was continuously changed (flow rate of 2 mL/min) to ACSF with 100 µM of ATP. After the 10 min recording in ATP‐containing ACSF. The entire recording time was 20 min for each cell. The extracellular recording solution consisted of ACSF supplemented with picrotoxin (100 µM) and tetrodotoxin (1 mM).

All electrophysiology data were acquired and analyzed using pClamp 10.5 (Molecular Devices). Signals were filtered at 2 kHz and digitized at 10 kHz using Digidata 1550A (Axon Instruments, Union City, CA, USA).

### 4.7 GABA, Glutamate, D‐serine, and Extracellular ATP Assays

GABA concentrations were obtained using LC/MS performed by Neurovis Co. (Cheonan, Korea). Glutamate, D‐serine, and extracellular ATP concentrations were measured using kits according to the respective manufacturer instructions. All assays were performed using a 96‐well microplate. Glutamate was measured using a colorimetric assay kit (#K629‐100; Biovision, Milpitas, CA, USA), and D‐serine was measured with a fluorometric assay kit (#K743‐100; Biovision).

For the extracellular ATP assays, briefly, dPAG slices were collected from ChR2‐expressing hGFAP‐ChR2 mice. The dPAG slices were incubated in ice‐cold oxygenized ACSF for 10 min, and then samples were collected and labeled as ‘light‐off.’ The ice‐cold oxygenated ACSF was replaced, and the dPAG slices were subjected to light stimulation for 10 min. The ACSF was then collected and temporarily stored at −80°C. The ATP level was determined using a bioluminescent ATP assay kit (FF2000; Promega, Madison, WI, USA) and measured using a luminometer (SPARK 10 M, Tecan, Grödig, Austria). The GABA, glutamate, D‐serine, and ATP amounts were normalized to the total protein amounts in each sample.

### 4.8 Pulse Rate Measurement

Pulse rates were measured via a rodent arterial pressure analysis system (BP‐2000 Blood Pressure Analysis System Series 1, Visitech Systems, Seoul, Korea), a computerized, non‐invasive tail‐cuff system that was described previously [49]. The mice were placed on the apparatus platform, which had a controlled temperature of 35°C, and habituated to it for 3 days before the actual data acquisition. Mice were individually placed inside a magnetic restrainer, and their tails were passed through the cuff and pulse sensor, which were connected to the control unit of the apparatus. On the day of the actual data acquisition, the mice were habituated in the apparatus for 10 min prior to the actual pulse rate recording for the experimental sessions: pre‐stimulation/pre‐exposure, optogenetic astrocyte stimulation, and CO_2_ exposure. Data were collected on a computer using BP‐2000 analysis software.

### 4.9 Primary Astrocyte Culture and In Vitro Ca^2+^ Assay

Primary astrocytes were isolated from the midbrains of postnatal day 1 hGFAP‐ChR2 mice and prepared using a modified established protocol [50]. Briefly, after removing the meninges from the cerebral hemisphere, the tissue was dissociated into a single‐cell suspension by gentle repetitive pipetting and was filtered through a 70‐mm filter (#352350, Falcon, NY, USA). The cells were cultured in DMEM supplemented with 10 mM HEPES, 10% FBS, 2 mM L‐glutamine, and 1x antibiotic/antimycotic in 75 cm^2^ flasks at 37°C in a 5% CO_2_ incubator. The medium was changed every 5 days. After 3 weeks, the flask was shaken at 250 rpm for 2 h at 37°C, treated with 100 mM L‐leucine methyl ester for 60 min to remove microglial cells, and harvested using trypsinization (0.25% trypsin, 0.02% EDTA). The primary cells were plated on PDL‐coated cover glasses and incubated. After seeding, the cells were treated with 4‐OH‐tamoxifen (1 mM, #H7904, Sigma‐Aldrich) for 2 days with new medium. Calcium activity in astrocytes expressing ChR2 was measured with single‐cell calcium imaging using Rhod‐2‐AM (Invitrogen, Carlsbad, CA, USA). The cells were incubated for 30 min at 37°C with 2 mM Rhod‐2‐AM in HBSS containing 25 mM HEPES (pH 7.5) and then washed with HBSS‐HEPES before the Ca^2+^ assays were conducted. A baseline reading was taken for 2 min before the blue light (473 nm, ∼3 mW) stimulation. Intracellular calcium levels were measured via digital video microfluorometry with an intensified charge‐coupled device camera (CasCade, Roper Scientific, Trenton, NJ, USA) coupled to a microscope, and they were analyzed with MetaFluor software (Universal Imaging Corp., Downtown, PA, USA). The fluorescence intensity data were z‐scored for each trace.

### 4.10 Imaging Intracellular Astrocyte Ca^2+^ Activity in Acute PAG Slices

Eight‐week‐old Wild‐type mice received a bilateral injection of 0.5 µL of AAV‐GFAP‐ChR2‐GFP and AVVDJ‐GFAP‐jRGECO1a‐mCherry in the PAG. After 3 weeks, the brains were rapidly removed and placed in an ice‐cold, oxygenated (95% O_2_ and 5% CO_2_), low‐Ca^2+^/high‐Mg^2+^ dissection buffer containing 5 mM KCl, 1.23 mM NaH_2_PO_4_, 26 mM NaHCO_3_, 10 mM dextrose, 0.5 mM CaCl_2_, 10 mM MgCl_2_, and 212.7 mM sucrose. Transverse PAG slices (300 µm) were transferred to a holding chamber in an incubator containing oxygenated (95% O_2_ and 5% CO_2_) artificial cerebrospinal fluid (ACSF) composed of 124 mM NaCl, 5 mM KCl, 1.23 mM NaH_2_PO_4_, 26 mM NaHCO_3_, 10 mM dextrose, 2.5 mM CaCl_2_, and 1.5 mM MgCl_2_ at 28°C–30°C for at least 30 min before imaging. After confirming both ChR2 (GFP) and jRGECO1a (mCherry) expressions, jRGECO1a‐positive fluorescence activities were imaged using a confocal microscope (LSM800). After imaging basal activities (1 min), a continuous light (473 nm) was delivered for 5 min. Then, the light was turned off and the fluorescent activities were imaged for 2 min. After imaging the fluorescent activities, jRGECO1a‐positive cells were selected (ROIs selection) and analyzed using an *EZcalcium*, an open‐source toolbox for the analysis of calcium imaging data. Data processing, including motion correction and ROI detection/refinement, was performed according to the developer's instruction. All background signals were corrected, and the obtained data were normalized as Z‐score.

### 4.11 Immunohistochemistry

Mice were transcardially perfused with ice‐cold 0.1 M PBS (pH 7.4) until all blood was removed, and that was followed by perfusion with ice‐cold 4% paraformaldehyde in 0.1 M PBS. Whole brains were post‐fixed in 4% paraformaldehyde in 0.1 M PBS overnight at 4°C and cryoprotected with 30% sucrose for 3 days. The brains were then cryosectioned into 40‐µm‐thick coronal sections and incubated in cryoprotectant at –20°C until immunohistochemical staining was performed. The sections were incubated in a blocking solution containing 5% normal donkey serum (Jackson ImmunoResearch, Bar Harbor, ME, USA), 2% BSA (Sigma‐Aldrich), and 0.1% Triton X‐100 (Sigma‐Aldrich) for 1.5 h at room temperature. Subsequently, the sections were incubated with mouse anti‐NeuN (MAB377B, 1:1,000; Millipore, Billerica, MA, USA), mouse anti‐GFAP (MAB360, 1:1,000; Millipore), rabbit anti‐S100ß (ab52642, 1:500; Abcam, Cambridge, MA, USA), rabbit anti‐Iba1 (019‐19741, 1:1000; Wako, Richmond, VA, USA), mouse anti‐CamKII (sc‐5035, 1:1,000; Santa Cruz Biotechnology, Dallas, TX, USA), mouse anti‐GABA (A0310, 1:1000; Sigma‐Aldrich), rabbit anti‐vGlut2 (ab216463, 1:1,000; Abcam), rabbit anti‐PSD95 (#51‐6900, 1:1000; Invitrogen), mouse anti‐Gephyrin (#147011, 1:1000; Synaptic systems, Göttingen, Germany), VGAT (#131006, 1:1,000; Synaptic systems), mouse anti‐PDGFRa (AF1062, 1:1000; R&D systems, Minneapolis, MN, USA), and mouse anti‐CC1 (OP‐80, 1:1000; Millipore) and rabbit anti‐c‐Fos (PC05‐100UG, 1:1000; Millipore) antibodies overnight at room temperature in a blocking solution. After washing them with 0.1 M PBS containing 0.1% Triton X‐100, we incubated the sections for 1 h with FITC‐, cy3‐, or cy5‐conjugated secondary antibodies (1:200, Jackson ImmunoResearch) in blocking solution at room temperature, washed them three times, and then mounted them on gelatin‐coated glass slides using Vectashield (Vector Laboratories, Inc., Burlingame, CA, USA). Fluorescent images of the mounted sections were obtained using a confocal microscope (LSM800; Carl Zeiss, Jena, Germany).

### 4.12 Image Acquisition and Quantification

Image acquisition was performed using a Carl Zeiss confocal laser scanning microscope. Image quantification was performed on images collected using the 20x objective. Laser intensity and gain were kept constant between experiments. Quantification of the number of cells was performed manually using the Event function in ZEN 2.0 software within a defined region of interest in each tissue section. Quantification data were obtained using at least three mice and at least five tissue sections per mouse for each experimental group. Cell density quantification is presented as the fold change compared with the designated control group. All images were acquired in a single plane with the pinhole set to 1.

### 4.13 Western Blot Assay

The brains were removed from mice 2 days after PBS or L‐AAA injection, and the rostral to caudal region of the dPAG area was dissected on an ice‐cold metal stage and frozen at −80°C until further processing. To extract proteins for the GFAP analyses, individual tissue samples were weighed and homogenized in five volumes of ice‐cold buffer containing 20 mM Tris (pH 7.5), 5% glycerol, 1.5 mM EDTA, 40 mM KCl, 0.5 mM dithiothreitol, and protease inhibitors (No. 539131, Calbiochem, San Diego, CA, USA). The homogenates were centrifuged at 14 000 rpm for 1 h at 4°C. The supernatant was removed from each sample, and an aliquot was collected to determine the total protein concentration using the Pierce BCA assay (Thermo Fisher Scientific, Hampton, NH, USA). The proteins were separated using sodium dodecyl sulfate–polyacrylamide gel electrophoresis and transferred to a polyvinylidene difluoride membrane. The membranes were incubated with primary antibodies against GFAP (1:1000, DAKO) and β‐actin (1:5000, Sigma‐Aldrich). Proteins were detected with horseradish peroxidase–conjugated secondary antibodies using a West Save Gold western blot detection kit (Ab Frontier, Seoul, Korea). Signals were visualized using MicroChemi (DNR Bio‐imaging Systems, Jerusalem, Israel). The relative expression level of GFAP was determined using densitometry and normalized to the β‐actin expression level.

### 4.14 Statistics

Briefly, depending on the normality of data distribution, statistical significance for comparisons between two groups was determined using independent, paired t‐testing, Mann‐Whitney U‐test and Wilcoxon signed‐rank test. For multiple comparisons, one‐way and two‐way analysis of variance (ANOVA) followed by the Fisher's Least Significant Difference (LSD) post‐hoc analysis was used unless otherwise mentioned in the figure legends. All data are represented as the mean ± standard error of the mean (s.e.m.), and differences are considered statistically significant if the *p* value is less than 0.05. Statistical analyses were performed using SPSS (v25, IBM SPSS Statistics). Detailed data and statistical information were indicated in the Source data and Table S1.

## Author Contributions

E.B., W.H.C., and S.J.L. conceived and designed the research. E.B. and K.N. performed stereotactic surgery and optogenetic experiments. E.B., M.H., Y.J.K., and U.L. performed immunohistochemistry and acquisition of histology data. E.B., K.N., M.K., U.L., and Y.J.R. performed fiber photometry, behavioral tests and analysis. S.B.J. and K.N. supervised the fiber photometry data acquisition and analysis. E.B. and M.K. performed the in vivo microdialysis and analysis of data. E.B., K.N., U.L., and W.H.C. performed pulse measurement, cell culture experiments and in vitro Ca^2+^ assay. K.N. performed two‐photon microscopy and J.K.R. supervised two‐photon microscopy. K.N. performed electrophysiology and S.Y.C. supervised electrophysiology. E.B., K.N., W.H.C., and S.J.L. wrote the manuscript, and all authors commented on the manuscript. S.J.L. and W.H.C. supervised the project.

## Conflicts of Interest

The authors declare no conflict of interest.

## Supporting information




**Supporting File 1**: advs74113‐supp‐0001‐SuppMat.docx.

**Supporting File 2**: advs74113‐sup‐0002‐MovieS1.mp4.

**Supporting File 3**: advs74113‐sup‐0003‐MovieS2.mp4.

**Supporting File 4**: advs74113‐sup‐0004‐MovieS3.mp4.

**Supporting File 5**: advs74113‐sup‐0005‐MovieS4.mp4.

**Supporting File 6**: advs74113‐sup‐0006‐MovieS5.mp4.

**Supporting File 7**: advs74113‐sup‐0007‐MovieS6.mp4.

**Supporting File 8**: advs74113‐sup‐0008‐MovieS7.mp4.

**Supporting File 9**: advs74113‐sup‐0009‐MovieS8.mp4.

## Data Availability

All data are available in the main text, Source data, or the supplementary materials.
