# Supplementary material for: Midbrain PAG Astrocytes Modulate Mouse Defensive and Panic‐Like Behaviors
Source: Adv Sci (Weinh). 2026 Feb 4;13(19):e06062. doi: 10.1002/advs.202506062 (PMC13045357; doi:10.1002/advs.202506062)
Supplement: Supplementary file 1 — Supporting File 1: advs74113‐supp‐0001‐SuppMat.docx. [file ADVS-13-e06062-s004.docx]

Supplementary Information

Midbrain PAG astrocytes modulate mouse defensive

and panic-like behaviors

Ellane Barcelon^1,8,11^, Kyungchul Noh^1,9,11^, Minkyu Hwang^2^, Yoon-Jung Kim^9^, Unjin Lee^3^, Yeon Joo Ryu^3^, Je-Kyung Ryu^4^, Sang Beom Jun^5,6,7^, Se-Young Choi^1^, Woo-Hyun Cho^1,10^ *, Sung Joong Lee^1^ *

Affiliations

^1^ Department of Physiology and Neuroscience, Dental Research Institute, Seoul National University, School of Dentistry, Seoul 08826, Republic of Korea

^2^ Department of Brain and Cognitive Neurosciences, College of Natural Sciences, Seoul National University, Seoul 08826, Republic of Korea

^3^ Interdisciplinary Program in Neuroscience, College of Natural Sciences, Seoul National University, Seoul 08826, Republic of Korea

^4^ Department of Physics and Astronomy, Seoul National University, Seoul 08826, Republic of Korea

^5^ Department of Electronic and Electrical Engineering, Ewha Womans University, Seoul 03760, Republic of Korea

^6^ Graduate Program in Smart Factory, Ewha Womans University, Seoul 03760, Republic of Korea

^7^ Department of Brain and Cognitive Sciences, Ewha Womans University, Seoul 03760, Republic of Korea

^8^ Spatial Navigation and Memory Unit, National Institute of Neurological Disorders and Stroke, National Institutes of Health, Bethesda, MD 20892, USA

^9^ Department of Pharmacology, Ajou University School of Medicine, Suwon 16499, Republic of Korea

^10^ Institute for Neurological Therapeutics, Rutgers-Robert Wood Johnson Medical School, Piscataway NJ 08854, USA

^11^ These authors contributed equally to this work

* Corresponding authors:

Sung Joong Lee. Email [sjlee87@snu.ac.kr](mailto:sjlee87@snu.ac.kr)

Woo-Hyun Cho. Email [wj200@rwjms.rutgers.edu](mailto:wj200@rwjms.rutgers.edu)

The Supplementary Information consists of

1. Supplementary Movies (Supplementary Movies S1-S8)
2. Supplementary Figures (Supplementary Figure 1-15)
3. Supplementary Table (Table 1)

Supplementary Movies

Movie S1. Example trial of PAG astrocyte Ca^2+^ recording in various threat assays.

Movie S2. Example trial of PAG astrocyte Ca^2+^ recording in non-threat assays.

Movie S3. Optogenetic stimulation of PAG astrocytes evokes defensive-like responses in an empty open arena.

Movie S4. Optogenetic stimulation of PAG astrocytes disrupts the shelter-directed escape against innate threat attack.

Movie S5. Optogenetic stimulation of PAG astrocytes alters mice instinctive shelter-directed behavior.

Movie S6. Optogenetic stimulation of PAG astrocytes disrupts dPAG neuron-evoked shelter-directed escape.

Movie S7. PAG astrocyte ablation alters dPAG neuron-induced defensive response.

Movie S8. Inhibition of PAG astrocyte Ca^2+^ activity using hPMCA2w/b enabled mice to escape from the CO_2_–induced panic-like state.

Supplementary Figures


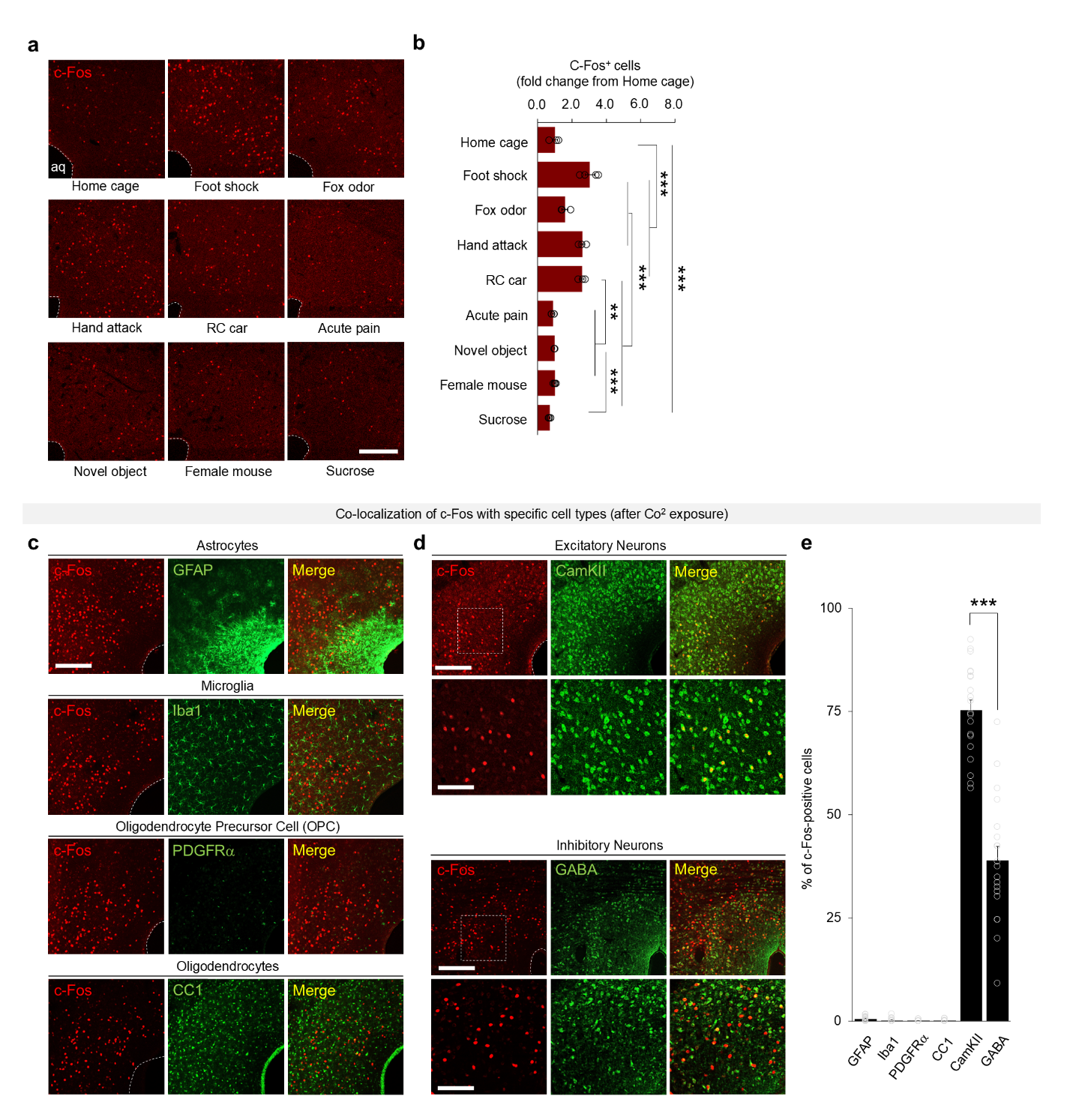


Supplementary Figure 1. Validation of threat stimuli via c-Fos expression in the dPAG area.

(a) Representative images of dPAG c-Fos expression (red) upon exposure to different stimuli. Scale bar, 200 μm. (b) Quantification of c-Fos-positive cells in the dPAG area after exposure to different stimuli (n = 3 - 4 mice, 3–4 slices per mouse, one-way ANOVA followed by LSD post-hoc analysis, ***p* < 0.01, ***p* < 0.001). Co-localization of c-Fos with (c) astrocytes (GFAP), microglia (Iba1), oligodendrocyte precursor cell (OPCs; PDGFRα), and oligodendrocyte (CC1). Scale bar, 200 μm. Co-localization with (d) excitatory neurons (CamKII) and inhibitory neurons (GABA). Scale bar, 200 μm and 100 μm (inset). (e) Quantification of c-Fos-positive cells across different cell types (Two-tailed Mann-Whitney *U* -test, ****p* < 0.001). Data are presented as the mean ± s.e.m. See Supplementary table 1 for detailed values and statistics.


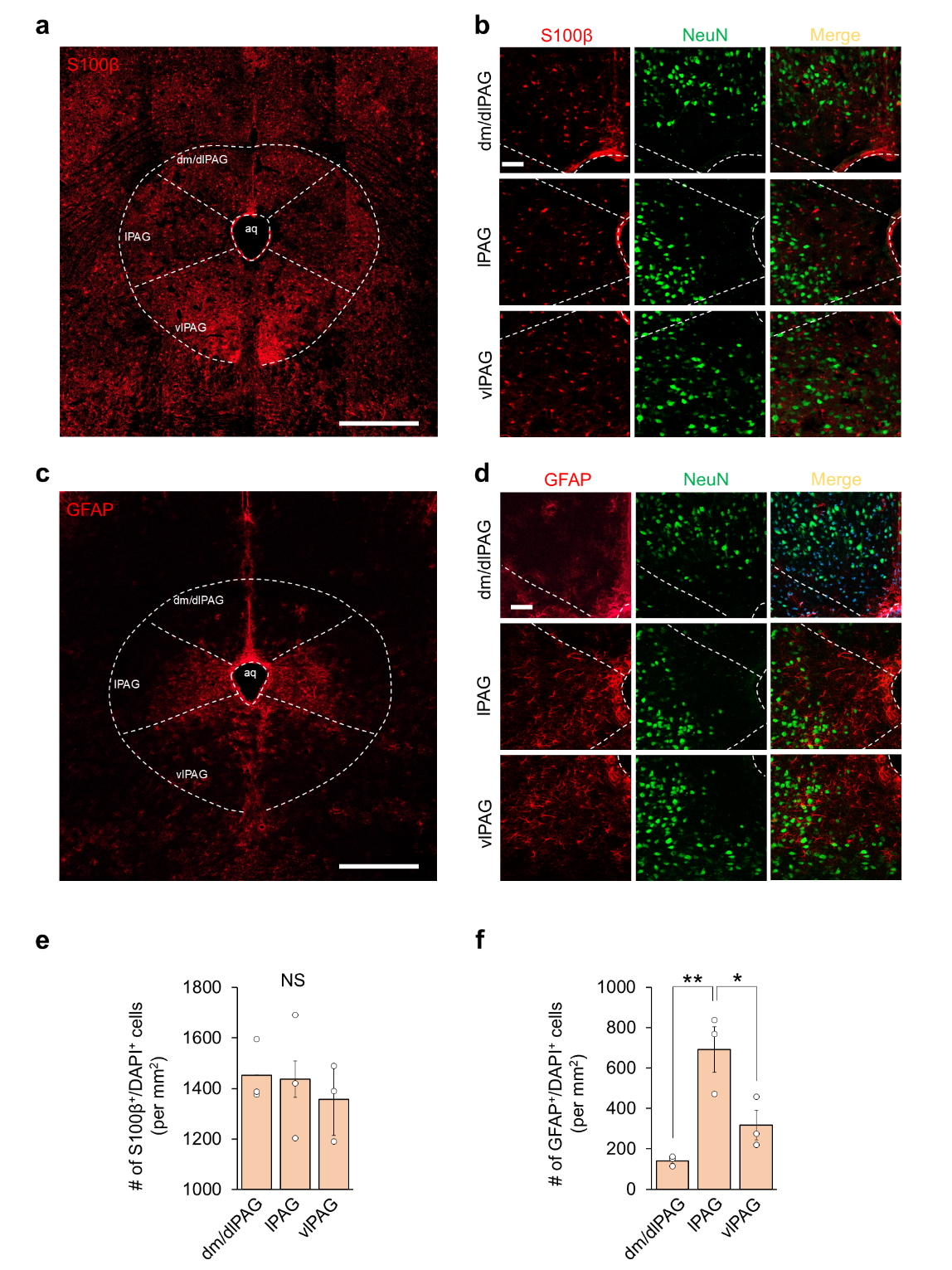


Supplementary Figure 2. Astrocyte density in mouse PAG columns.

(a) Representative images of S100β^+^ astrocytes (red) and (b) its NeuN overlap along PAG columns: dorsal (dPAG), lateral (lPAG), and ventral (vPAG) columns from Bregma -3.80 mm to -4.84 mm. (c) Representative images of GFAP^+^ astrocytes (red) and (d) its NeuN overlap along PAG columns: dorsal (dPAG), lateral (lPAG), and ventral (vPAG) columns from Bregma -3.80 mm to -4.84 mm. (e and f) Quantification of S100b or GFAP^+^ cells along with DAPI in PAG columns (n = 3 mice, 4 tissue sections per mouse; one-way ANOVA followed by Bonferroni post hoc analysis).


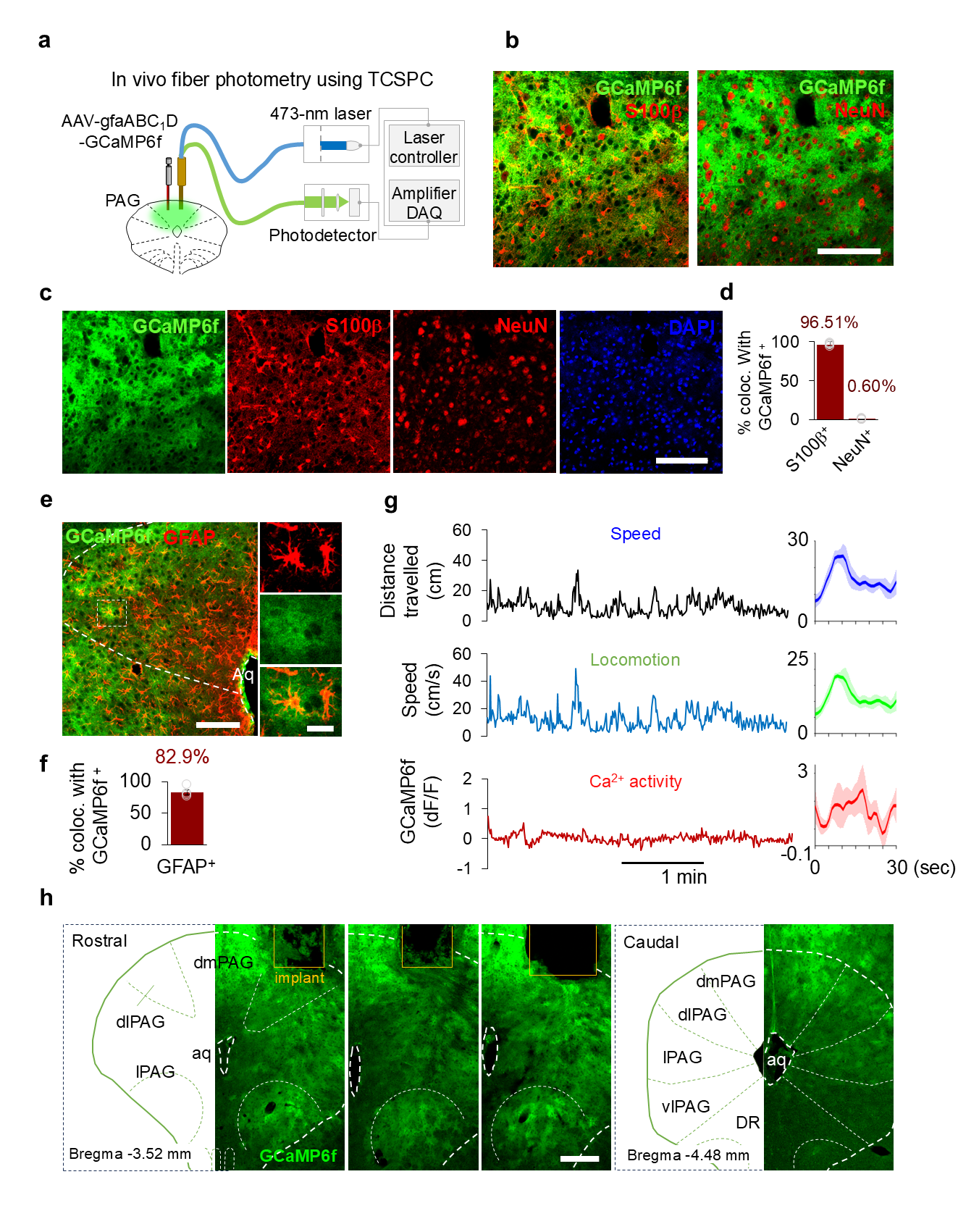


Supplementary Figure 3. PAG astrocyte Ca^2+^ activity recording using fiber photometry.

(a) Schematic diagram of *in vivo* Ca^2+^ recording using TCSPC. A TCSPC probe was implanted into the dorsal PAG of a mouse expressing GfaABC1D-GCaMP6f. The TCSPC probe was connected to a laser controller emitting 473 nm blue light and a photodetector for observing Ca^2+^ activity. (b) Representative images of PAG GfaABC1D-GCaMP6f (green) colocalization with S100β^+^ (red, left image) astrocytes but not Neun-positive cells (red, right image). Scale bar, 100 μm. (**c**) Representative images of the individual channels and expression of GfaABC1D-GCaMP6f (green), S100β (red), NeuN (red), and DAPI (blue) in PAG. Scale bar, 100 μm. (d) Quantification of the colocalization percentage of S100β^+^ and NeuN^+^ cells with GCaMP6f (S100β^+^, n = 6 mice; NeuN^+^, n = 6 mice; 2 sections per mouse). (e) Representative image of GfaABC1D-GCaMP6f (green) with GFAP^+^ astrocytes (red, left). Scale bar, 100 μm. Individual channels on the right. Scale bar, 50 μm. (f) Quantification of the colocalization percentage of GFAP^+^ cells with GCaMP6f (n = 5 mice, 3-4 sections per mouse). (g) General locomotor activity measured by distance travelled (black line, top panel) and speed (blue line, middle panel) during PAG astrocyte Ca^2+^ activity (red line, bottom panel) recording. (h) Representative images of GfaABC1D-GCaMP6f virus expression in PAG and optic fiber implantation in dorsal PAG from Bregma -3.52 mm to -4.48mm. dPAG: dorsal PAG, dmPAG: dorsomedial PAG, dlPAG: dorsolateral PAG, lPAG: lateral PAG, aq: aqueduct, vlPAG: ventrolateral PAG, DR: dorsal raphe nucleus. Scale bar, 200 μm. Data are presented as the mean ± s.e.m.


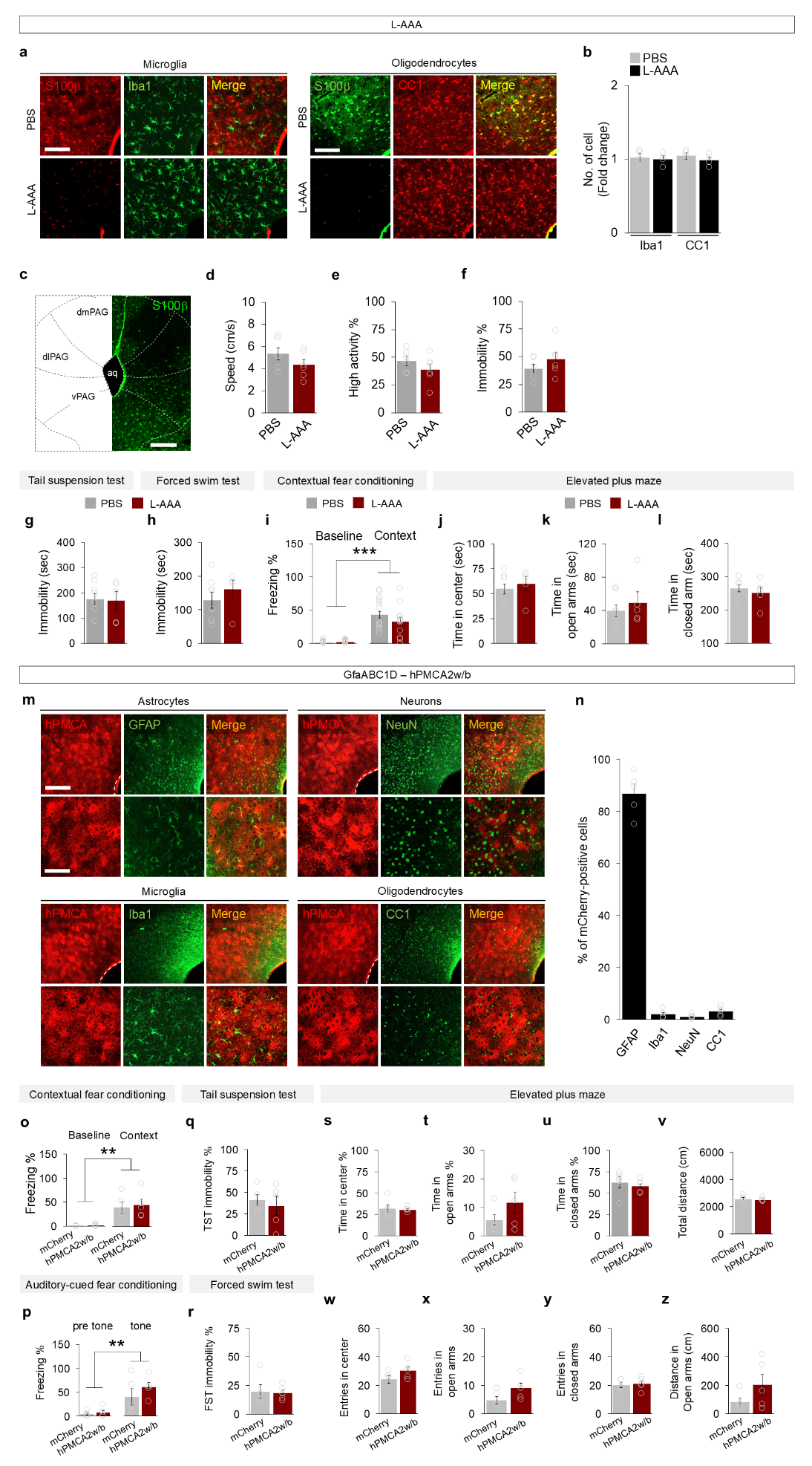


Supplementary Figure 4. PAG astrocyte ablation and baseline behavioral measurements.

(a) Representative images showing the co-localization of astrocytes (S100b) with microglia (Iba1), and astrocytes with oligodendrocyte (CC1) following L-AAA injection into the PAG. Scale bar, 200 μm. (b) Quantification of cell density (Iba- or CC1-positive cells). (Two-tailed Mann-Whitney *U* -test, *p* = 0.886 for Iba1, *p* = 0.343 for CC1). (c) Representative image of astrocyte ablation (S100β, green) by L-AAA injection located in dorsal PAG but not in other columns of PAG. Scale bar, 200μm. (d) General locomotor activity of PBS- (n = 6 mice) and L-AAA –injected mice (n = 6) before exposure to threats, measured by speed (Two-tailed Mann-Whitney *U* -test, *p* = 0.262), (e) percentage of high activity (Two-tailed Mann-Whitney *U* -test, *p* = 0.262), and (f) percentage of immobility (PBS n = 6 mice, L-AAA n = 6 mice; two-tailed Mann-Whitney *U*-test *p* = 0.200). (g) Immobility duration during ta and il suspension test and (h) forced swim test (PBS n = 8, L-AAA n = 8; two-tailed Mann-Whitney *U*-test *p* = 0.262). (i) Freezing percentage during the context fear conditioning (PBS n = 18 mice, L-AAA n = 13 mice, two-way repeated ANOVA ****p* < 0.001 followed by two-tailed Mann-Whitney *U*-test *p* = 0.464). (j) Time that mice spent in the center (Two-tailed Mann-Whitney *U* -test, *p* = 0.570), (k) open arms (Two-tailed Mann-Whitney *U* -test, *p* = 0.570), and (l) closed arms of the elevated plus maze (PBS n = 7, L-AAA n = 5; two-tailed Mann-Whitney *U* -test, *p* = 0.465). (m) Representative image showing hPMCA2w/b-expressing cell (mCherry) together with astrocytes (GFAP), neurons (NeuN), microglia (Iba1), and oligodendrocytes (CC1) in the PAG. Scale bar, 200 μm and 100 μm. (n) Quantification of mCherry-positive cells colocalized with each cell type. (o) Freezing percentage in baseline vs contextual fear conditioning (mCherry n = 5, hPMCA2w/b n = 5, two-way repeated ANOVA ***p* = 0.001 followed by two-tailed Mann-Whitney *U*-test *p* = 0.917). (p) Auditory-cued fear conditioning during pre tone and tone exposure (mCherry n = 5, hPMCA2w/b n = 5, two-way repeated ANOVA ***p* = 0.004 followed by two-tailed Mann-Whitney *U*-test *p* = 0.465). (q) Immobility percentage during tail suspension test (mCherry n = 5, hPMCA2w/b n = 5; two-tailed Mann-Whitney *U*-test *p* = 0.754) and (r) forced swim test (Two-tailed Mann-Whitney *U*-test *p* = 0.754). (s) Percentage of time spent in the center (Two-tailed Mann-Whitney *U*-test *p* = 0.602), (t) open arms (Two-tailed Mann-Whitney *U*-test *p* = 0.347), (u) closed arms (Two-tailed Mann-Whitney *U*-test *p* = 0.251), and (v) the total distance in the elevated plus maze ((Two-tailed Mann-Whitney *U*-test *p* = 0.465). (w) The number of entries in the center (Two-tailed Mann-Whitney *U*-test *p* = 0.172), (x) open arms (Two-tailed Mann-Whitney *U*-test *p* = 0.093), (y) closed arms (Two-tailed Mann-Whitney *U*-test *p* = 0.597), and (z) the distance in the open arms of the elevated plus maze (mCherry n = 5, hPMCA2w/b n = 5, two-tailed Mann-Whitney *U*-test *p* = 0.251). Data are presented as the mean ± s.e.m. See Supplementary table 1 for detailed values and statistics.


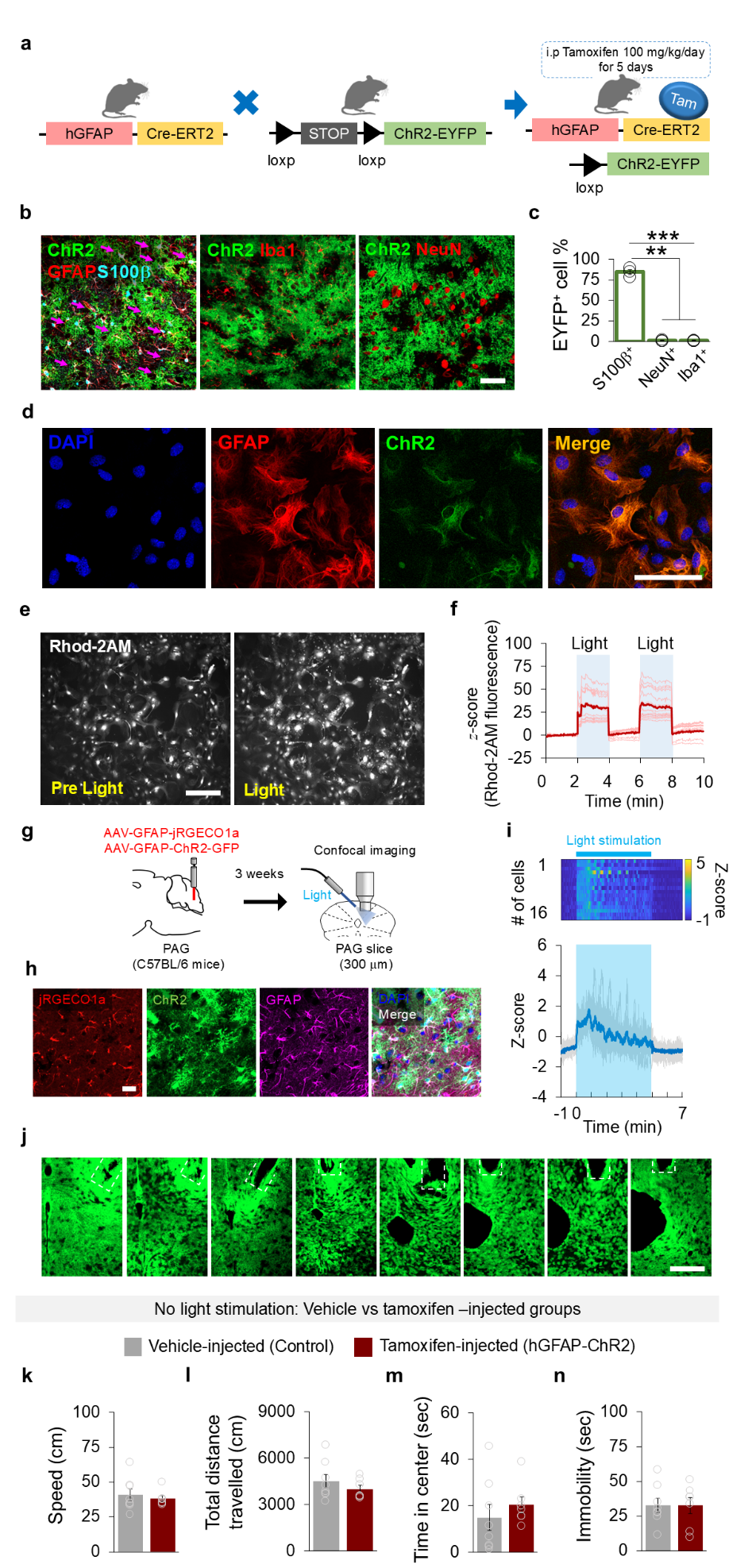


Supplementary Figure 5. Optogenetic stimulation increases astrocyte Ca^2+^ activity *in vitro.*

(a) Schematic illustration of the protocol for generating hGFAP-ChR2 mice by crossing hGFAP-CreERT2 and ChR2-EYFP mice; expression is induced by an intraperitoneal injection of 100 μg/g of tamoxifen daily for 5 days. (b) Representative images of ChR2-EYFP (green) expression showing that it colocalized with S100β^+^ (cyan) and GFAP^+^ (red) astrocytes but not Iba-1^+^ (red) or NeuN^+^ (red) cells. Purple arrows indicate merge with ChR2-eYFP; scale bar, 50 μm. (c) Percentage of S100β^+^ and GFAP^+^, NeuN^+^, and Iba-1^+^ cells expressing ChR2-EYFP (n = 3 mice, 4 slices per mouse; one-way ANOVA followed by Bonferroni post-hoc test **p < 0.01, ***p < 0.001). (d) Representative images of hGFAP-ChR2 pup midbrain primary astrocytes expressing ChR2 (green) stained with DAPI (blue), GFAP (red), and their merger. Scale bar, 100 μm. (e) Representative images of the Rhod-2-AM signal from *in vitro* Ca^2+^ imaging of hGFAP-ChR2 primary astrocytes before and after blue light exposure. Scale bar, 50 μm. (f) Ca^2+^ activity *z*-scored traces from midbrain primary astrocytes in response to light stimulations *in vitro* (n = 14 cells). (g) Experimental scheme for recording PAG astrocyte Ca^2+^ activity in acute brain slices. (h) Representative images of jRGECO1a expression in PAG astrocytes. Scale bar, 20 μm. (i) Heatmap and representative traces of PAG astrocyte Ca^2+^ activities with or without optogenetic stimulation. Individual traces (gray) and mean trace (blue) are shown. (j) Implantation of optic fiber in the PAG of hGFAP-ChR2 mice. Scale bar, 500 μm. (k) General locomotor activity in speed of vehicle (n = 8 mice) vs tamoxifen-injected mice (n = 7 mice) without light stimulation (Two-tailed Mann-Whitney *U*-test *p* = 0.817), (l) total distance travelled (Two-tailed Mann-Whitney *U*-test *p* = 0.355), (m) time spent in the center (Two-tailed Mann-Whitney *U*-test *p* = 0.271), and (n) immobility duration in the open arena (Vehicle-injected (Control) n = 8, Tamoxifen-injected (hGFAP-ChR2) n = 8, two-tailed Mann-Whitney *U*-test *p* = 0.728). Data are presented as the mean ± s.e.m. See Supplementary table 1 for detailed values and statistics.


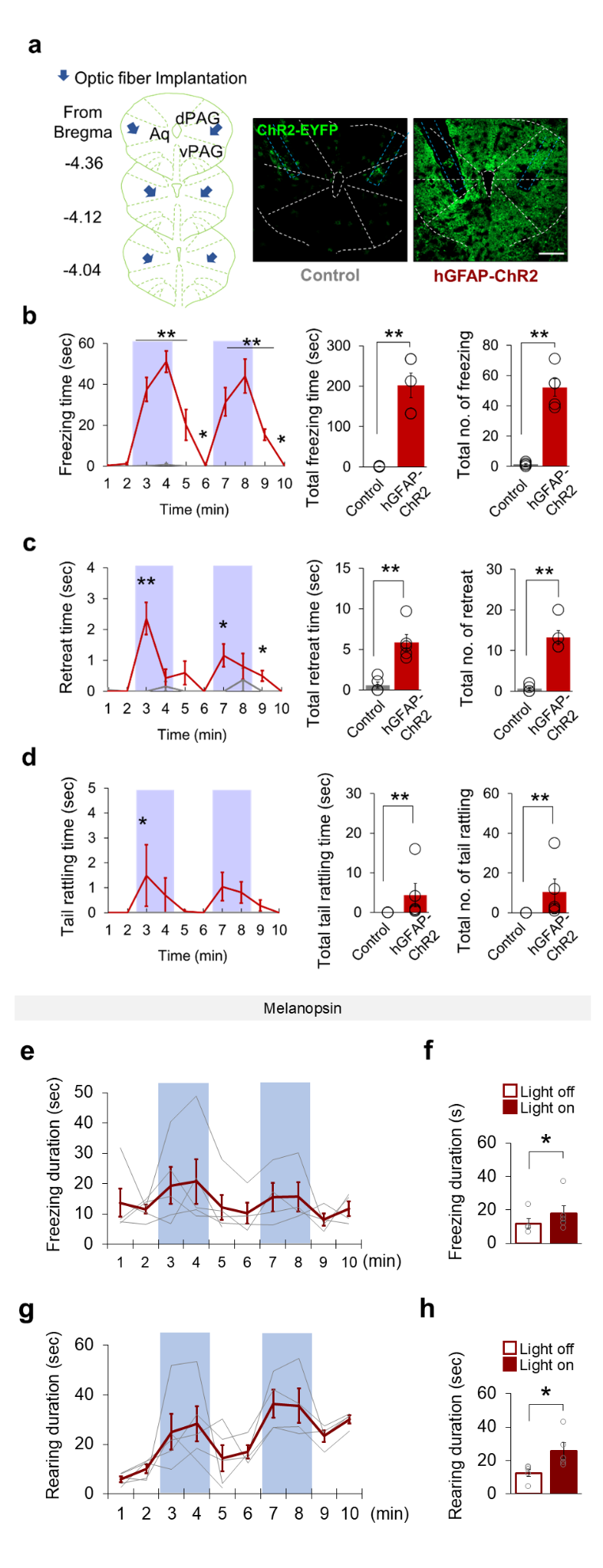


Supplementary Figure 6. Optogenetic activation of ventral PAG astrocytes and melanopsin-induced PAG astrocyte activation evokes defensive behaviors.

(a) Optic fiber placements targeting ventral PAG and representative images of vPAG optic fiber implantation in control and hGFAP-ChR2 mice. (b) Freeze duration, (c) retreating time, and (d) tail-tattling time during light-off and light-on (blue shade, continuous stimulation) epochs in a 10-min session in an open arena (left panels), the total duration (middle panels), and the total number of events (right panels) between control and hGFAP-ChR2 mice (n = 5 per group; two-tailed Mann-Whitney U-test for each minute between control and hGFAP, ***p* < 0.01, **p* < 0.05). (e and f) Freezing duration during light-off and light-on (blue shade) epochs during a 10-min session in an open arena (n = 5, Two-tailed Mann-Whitney *U*-test, **p* = 0.043). (g and h) The average freezing duration during light off and light on in GFAP-melanopsin-injected mice (n = 5, two-tailed Mann-Whitney U-test, **p* = 0.043). Data are presented as the mean ± s.e.m. See Supplementary table 1 for detailed values and statistics.


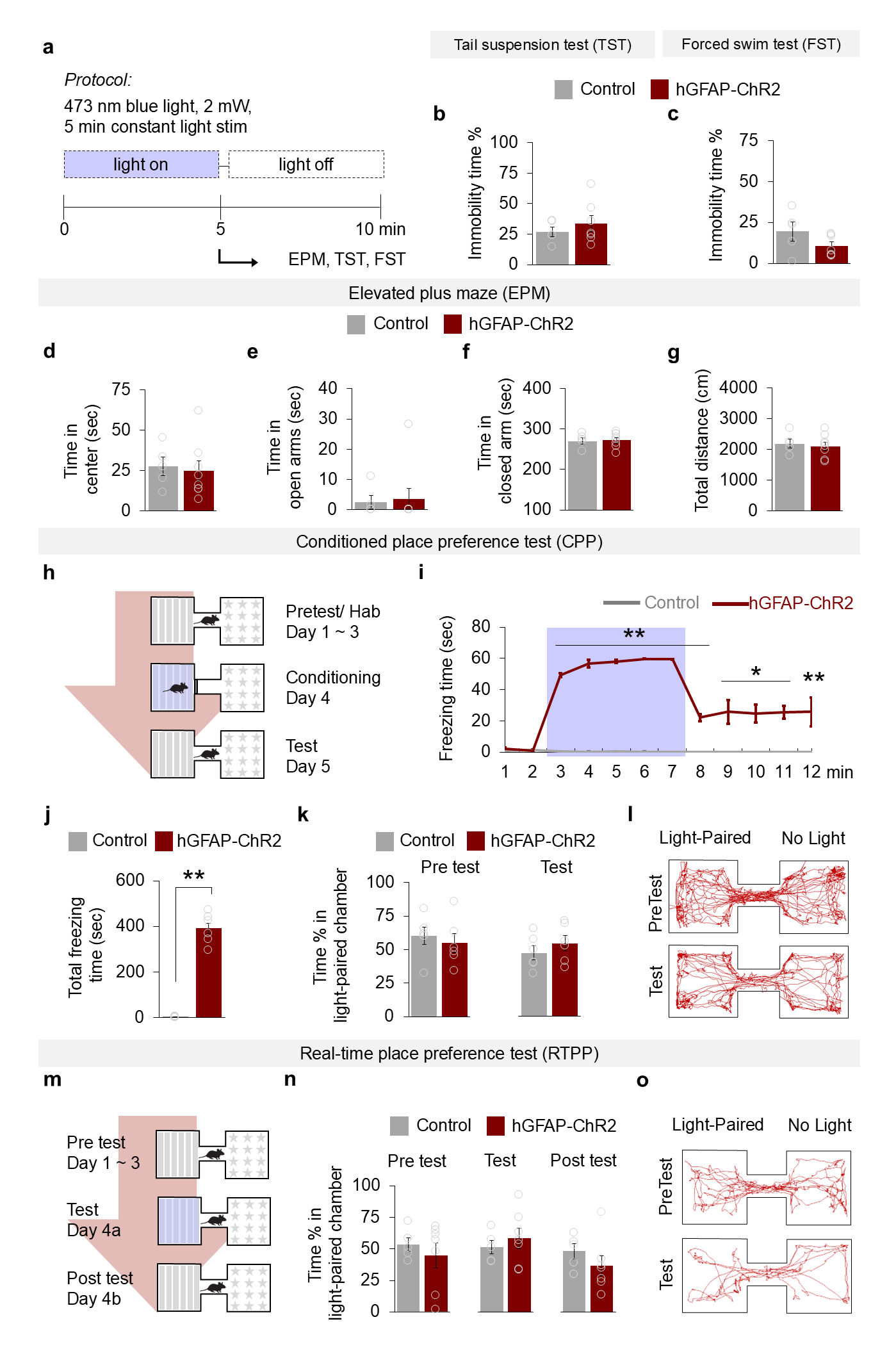
Supplementary Figure 7. Acute post-optogenetic PAG astrocyte activation does not affect other mouse behaviors.

(a) Experimental scheme for behavioral tests performed after mice received 2 mW of constant blue light stimulation for 5 min. (b) The percentage of time mice were immobile during the tail suspension test (TST, two-tailed Mann-Whitney U-test *p* = 0.570) and (c) forced swim test (FST, control n = 5 mice, hGFAP-ChR2 n = 7 mice, two-tailed Mann-Whitney U-test *p* = 0.558). (d) The time mice spent in the center (control n = 5 mice, hGFAP-ChR2 n = 8 mice, two-tailed Mann-Whitney U-test *p* = 0.558), (e) open arms (Two-tailed Mann-Whitney U-test *p* = 0.373), and (f) closed arms of the EPM (Two-tailed Mann-Whitney U-test *p* = 0.884). (g) The locomotor activity of mice (total distance traveled) after they received 5 min of light stimulation (control n = 5 mice, hGFAP-ChR2 n = 8 mice, two-tailed Mann-Whitney U-test *p* = 0.770). (h) Schematic for the conditioned place preference (CPP) experiment. (i) The duration of freezing behaviors of control (n = 5 mice) and hGFAP-ChR2 mice (n = 6) during the 12 min session of conditioning, which involved light-off and light-on dPAG astrocyte stimulation (Two-tailed Mann-Whitney U-test **p* < 0.05, ***p* < 0.01, ****p* < 0.001). (j) The total time spent freezing during the conditioning phase (control n = 5 mice, hGFAP-ChR2 n = 8 mice, two-tailed Mann-Whitney U-test ***p* = 0.006). (k) The percentage of time that the control and hGFAP-ChR2 mice spent in the light-paired chamber during the pre-test and test sessions (n = 6 per group, two-way repeated ANOVA *p* = 0.308 followed by two-tailed Mann-Whitney U-test *p* = 0.521 (pre test) *p* = 0.337 (test)). (l) Sample traces of the locomotor activity of an hGFAP-ChR2 mouse in the no-light and light-paired chambers during the pretest and test sessions. (m) Schematic for the real-time place preference (RTPP) experiment. (n) The percentage of time control and hGFAP-ChR2 mice spent in the light-paired chamber during the pre-test, test, and post-test sessions (control n = 6 mice, hGFAP-ChR2 n = 6 mice, two-way repeated ANOVA *p* = 0.308 followed by two-tailed Mann-Whitney U-test *p* = 0.949 (pre test) *p* = 0.482 (test) *p* = 0.084 (post test)). (o) Representative sample traces of hGFAP-ChR2 locomotor activity in the no-light and light-paired chambers during the pre-test and test sessions of the RTPP. Data are presented as the mean ± s.e.m. See Supplementary table 1 for detailed values and statistics.


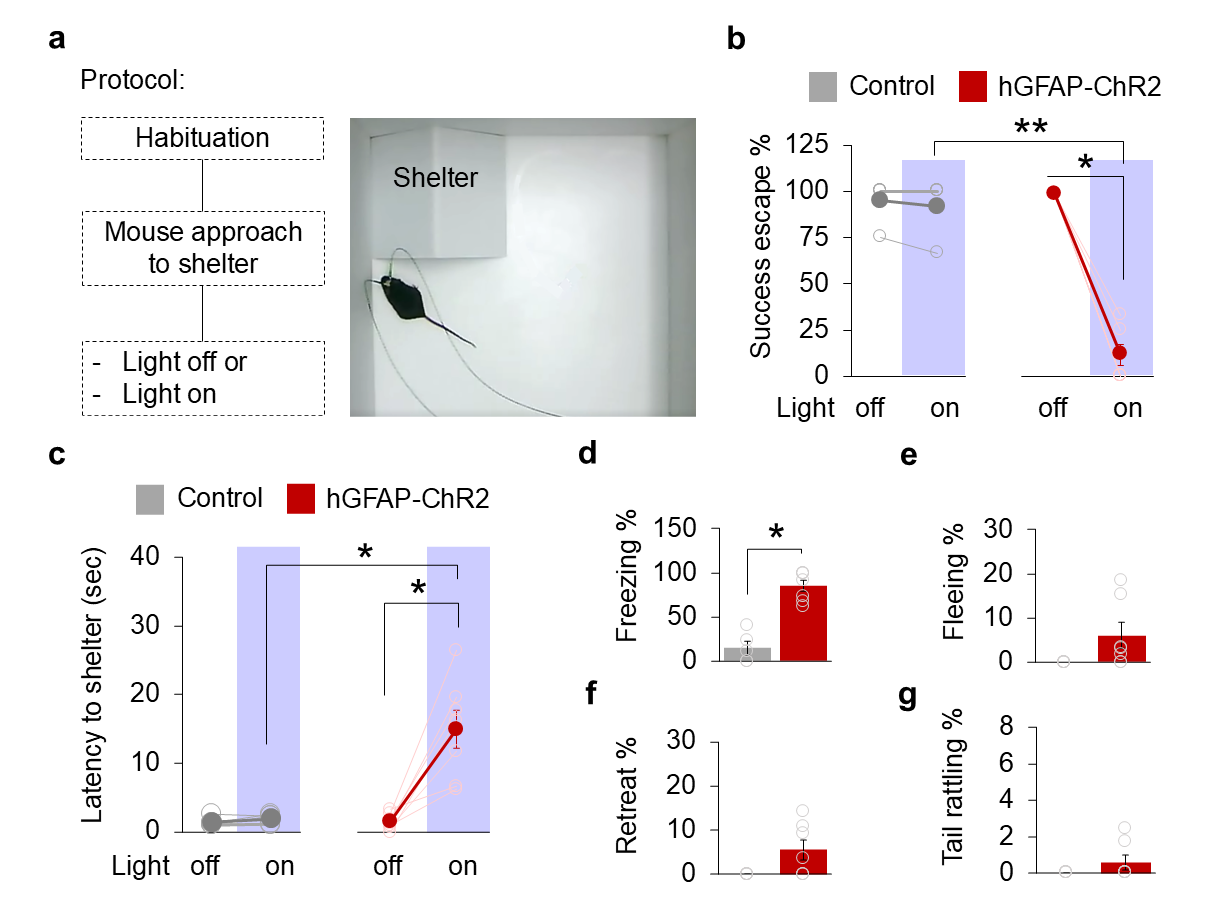


Supplementary Figure 8. Optogenetic PAG astrocyte activation disrupts mouse shelter-directed response.

(a) Behavioral protocol (left) that started with mouse habituation to the arena containing the shelter (right). On the test day, mice that approached the shelter received light stimulation. (b) Percentage of successful escapes by the control (n = 5 mice) and hGFAP-ChR2 mice (n = 7) during light-off (Wilcoxon signed-rank test *p* = 0.317) and light-on (shaded, Wilcoxon signed-rank test **p* = 0.014) epochs (Two-tailed Mann-Whitney *U* -test ***p* = 0.003 (Light on, Control vs hGFAP-ChR2)). A successful escape was scored when a mouse reached the shelter within 5 sec after the onset of light stimulation. (c) The time it took for mice to reach the shelter during light-off (Wilcoxon signed-rank test *p* = 0.131) and light-on epochs (Wilcoxon signed-rank test **p* = 0.043, two-tailed Mann-Whitney *U* -test **p* = 0.014 (Light on, Control vs hGFAP-ChR2)). (d) Percentage of time that the control (n = 4) and hGFAP-ChR2 mice (n = 5) spent freezing (Two-tailed Mann-Whitney *U* -test **p* = 0.014), (e) fleeing (Two-tailed Mann-Whitney *U* -test *p* = 0.180), (f) retreating (Two-tailed Mann-Whitney *U* -test *p* = 0.180), and (g) tail rattling during light-on epochs (Two-tailed Mann-Whitney *U* -test ***p* = 0.081). Data are presented as the mean ± s.e.m. See Supplementary table 1 for detailed values and statistics.


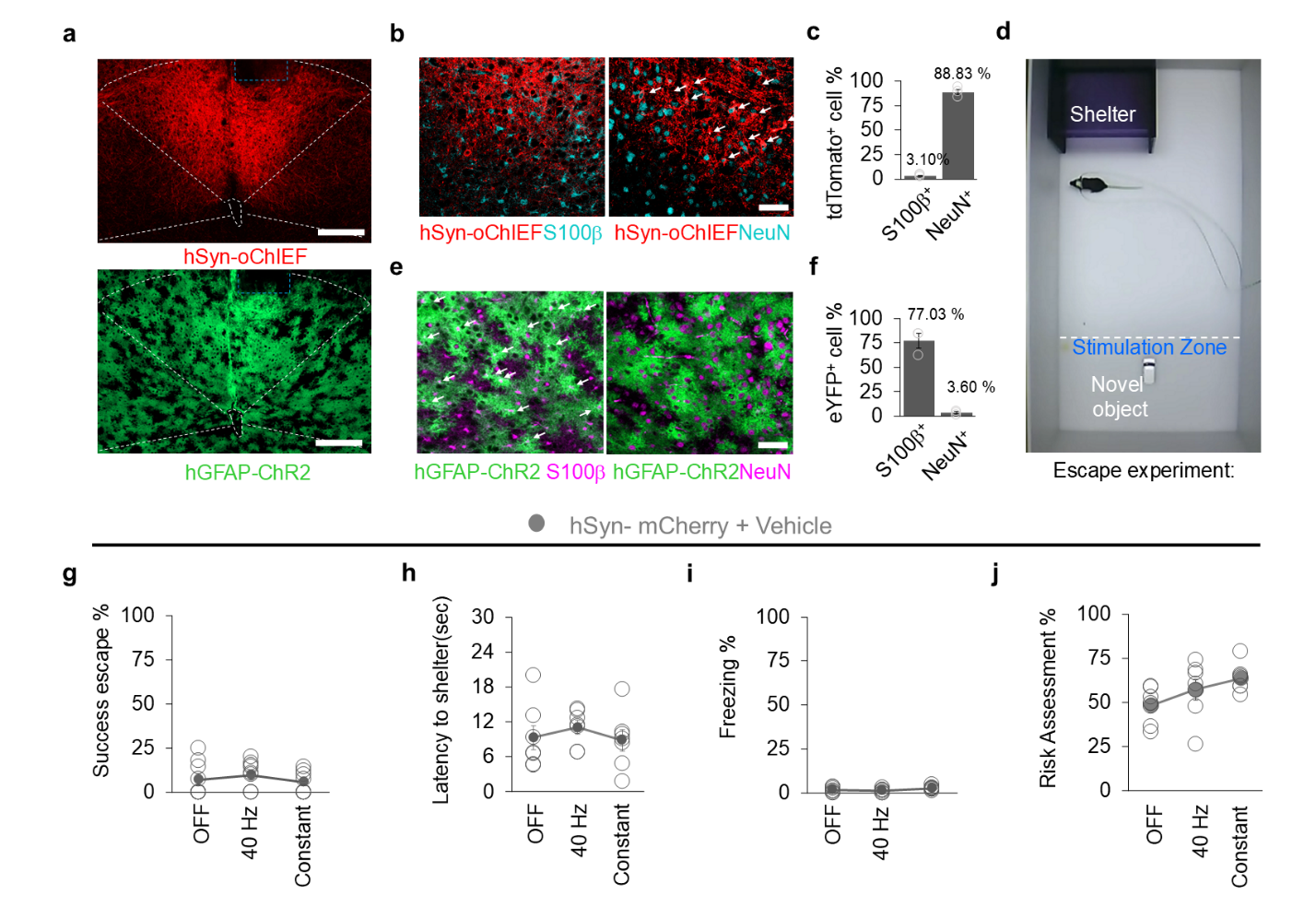
Supplementary Figure 9. Optogenetic manipulations of dPAG neurons and astrocytes.

(a) Representative image of hSyn-oChIEF-tdTomato (red, upper panel) virus expression in the dPAG area. Representative image of ChR2-eYFP (green) expression in the same dPAG tissue after tamoxifen injection. Scale bar, 200 μm. (b) Representative images of hSyn-oChIEF expression (red) colocalized with S100β^+^ astrocytes (cyan, left) and NeuN^+^ cells (cyan, right). White arrows indicate a merged signal. Scale bar, 50 μm. (c) Quantification of the percentage of hSyn-oChIEF-tdTomato that colocalized with S100β^+^ astrocytes and NeuN^+^ cells (n = 4 mice, 3–4 sections per mouse). (d) Set-up for the escape experiment, which consisted of a rectangular arena with a shelter/safe zone and a stimulation zone that contained a small novel object to induce the mice to explore the area opposite the shelter. (e) Representative images of ChR2-eYFP (green) and its colocalization with S100β^+^ astrocytes (purple, left) and NeuN^+^ cells (purple, right). White arrows indicate a merged signal. Scale bar, 50 μm. (f) Quantification of the percentage of hGFAP-ChR2-eYFP that colocalized with S100β^+^ astrocytes and NeuN^+^ cells (n = 4 mice, 3–4 sections per mouse). (g) Percentage of successful escapes by mice injected with control virus (hSyn-mCherry) or vehicle during light off and 40 Hz stimulation for 10 ms and constant epochs. (h) Latency to reach the shelter, (i) percentage of time spent in freezing behavior, and (j) risk assessment behavior by the control mice during light off and 40 Hz stimulation for 10 ms and constant epochs. (g–j) hSyn-mCherry + vehicle n = 7 mice, one-way ANOVA followed by LSD post hoc analysis. Data are presented as the mean ± s.e.m. See Supplementary table 1 for detailed values and statistics.


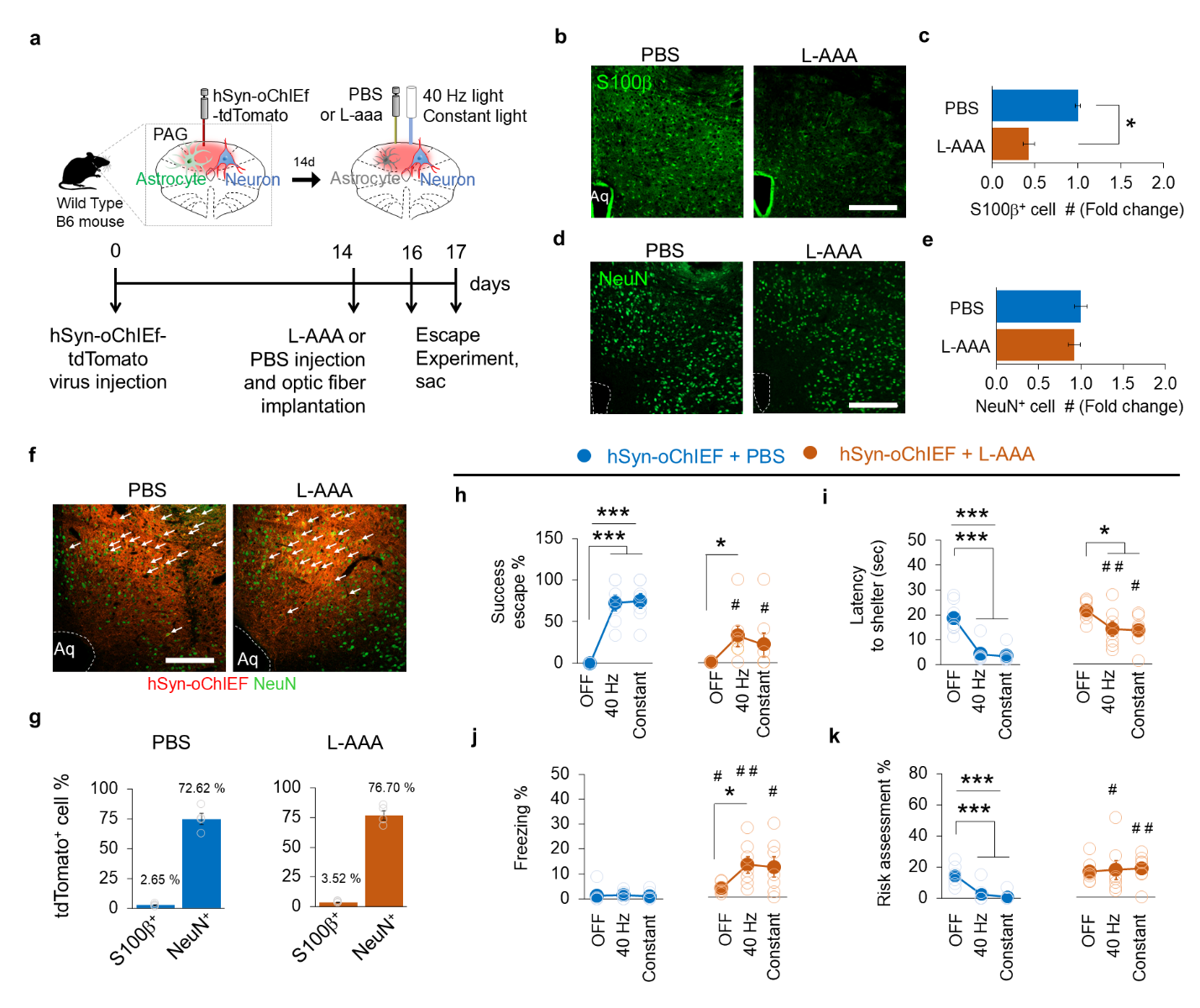
Supplementary Figure 10. Optogenetic stimulation of dPAG neurons and ablation of PAG astrocytes.

(a) Schematic for the optogenetic stimulation of dPAG neurons by injecting wild-type mice with hSyn-oChIEF-tdTomato and pharmacologic ablation of dPAG astrocytes with L-AAA. (b) Representative images and (c) normalized quantification of dPAG S100β^+^ astrocytes (green) in mice injected with PBS or L-AAA (PBS n = 4 mice, L-AAA n = 4 mice, 3 tissue sections per mouse, two-tailed Mann-Whitney *U* -test **p* = 0.021). Scale bar, 200 μm. (d) Representative images and (e) fold change quantification of dPAG NeuN^+^ cells (green, n = 4 mice per group, two-tailed Mann-Whitney *U* -test *p* = 0.386). *Aq,* aqueduct. Scale bar, 200 μm. (f) Representative images of hSyn-oChIEF-tdTomato expression (red) and its colocalization with NeuN^+^ cells (green). White arrows indicate a merged signal. Scale bar, 200 μm. (g) Quantification of the percentage of NeuN^+^ cells that were colocalized with hSyn-oChIEF-tdTomato (PBS n = 4, L-AAA n = 4 mice). (h) Percentage of successful escapes by hSyn-oChIEF + PBS (left) and hSyn-oChIEF + L-AAA (right) mice during light-off, 40 Hz stimulation for 10 ms and constant light epochs. A successful escape was scored when a mouse reached the shelter within 5 sec after the onset of light stimulation. (i) Latency to reach the shelter, (j) percentage of time spent in freezing behavior, and (k) percentage of time spent in risk assessment behavior among the hSyn-oChIEF + PBS (left) and hSyn-oChIEF + L-AAA (right) mouse groups during light-off, 40 Hz stimulation for 10 ms, and constant light epochs (hSyn-oChIEF + PBS n = 7 mice, hSyn-oChIEF + L-AAA n = 7 mice, one-way ANOVA followed by LSD post hoc analysis, **p* < 0.05, ****p* < 0.001, Student’s t-test to compare hSyn-oChIEF + PBS vs hSyn-oChIEF + L-AAA in each light condition, #*p* < 0.05, ##*p* < 0.01). Data are presented as the mean ± s.e.m. See Supplementary table 1 for detailed values and statistics.


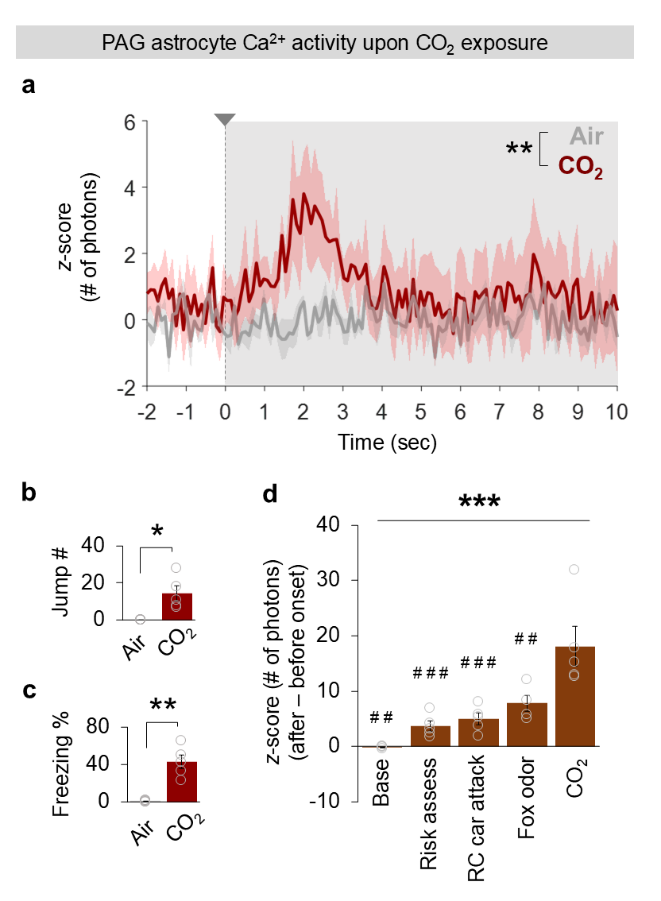


Supplementary Figure 11. CO_2_ exposure evoked distinct Ca^2+^ activity increase in PAG astrocytes

(a) PAG astrocyte Ca^2+^ activity recording trace before and during CO_2_ exposure (n = 5 mice, two-tailed paired t-test, ***p* = 0.009). Gray marker and shade indicates CO_2_ initiation and exposure. (b) The number of jumps (Wilcoxon signed-rank test, **p* = 0.043) and (c) the percentage of freezing before (air) and during CO_2_ exposure (n = 5, Wilcoxon signed-rank test, **p* = 0.043). (d) Change in the z-scored Ca^2+^ activity recorded during all the threat assays ([1–3 sec after] minus [0–2 sec before the onset of a threat stimulus], n = 4–5 mice, one-way ANOVA; ****p* < 0.001, followed by Dunnett’s t-test comparing the groups with CO_2_, #*p* < 0.05, ## *p* < 0.01, ###*p* < 0.001). Data are presented as the mean ± s.e.m. See Supplementary table 1 for detailed values and statistics.


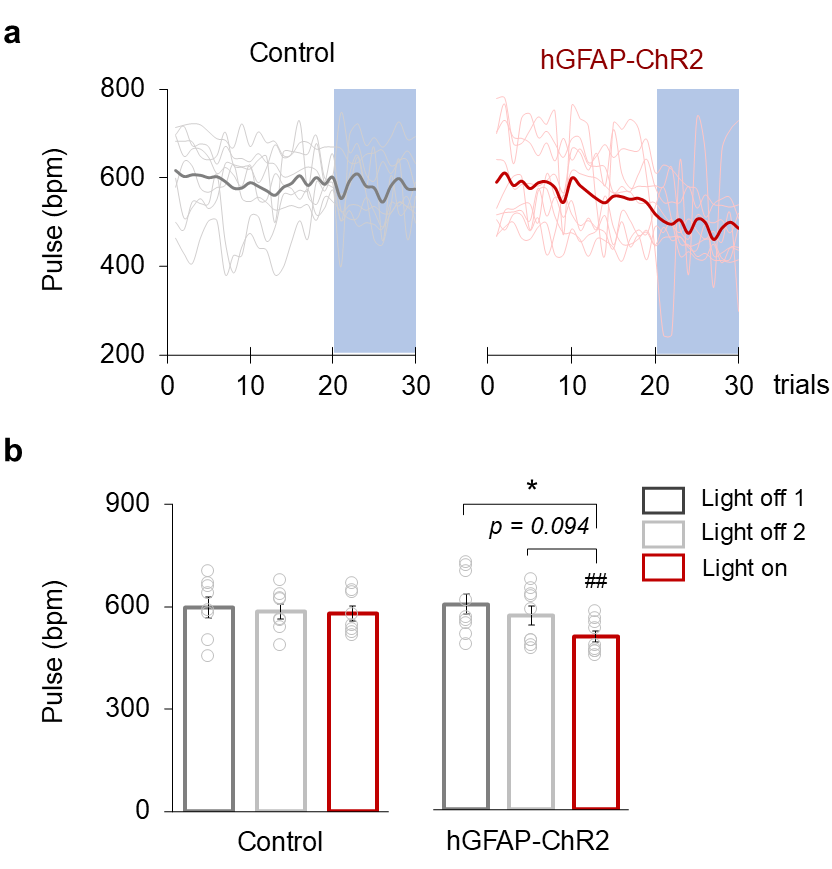


Supplementary Figure 12. Optogenetic dPAG astrocyte activation reduces mouse pulse rate.

(a) Pulse rate in bpm measured from control and hGFAP mice; the first 20 trials were a light-off epoch, and the last 10 trials were a light-on epoch with constant 2 mW light intensity (control n = 9, hGFAP-ChR2 n = 9 mice). (b) Quantification of the average pulse rate (bpm) of control and hGFAP-ChR2 mice during light-off and light-on epochs, where ‘Light off 1’ was the average of the first 10 trials (dark gray), ‘Light off 2’ was the average of the second 10 trials (light gray), and ‘Light on’ was the average of the last 10 trials in which light was delivered (red, one-way repeated ANOVA followed by LSD post hoc analysis paired t-test, **p* < 0.05, ***p* < 0.01, two-tailed Mann-Whitney *U*-test during Light on ***p* = 0.009). Data are presented as the mean ± s.e.m. See Supplementary table 1 for detailed values and statistics.


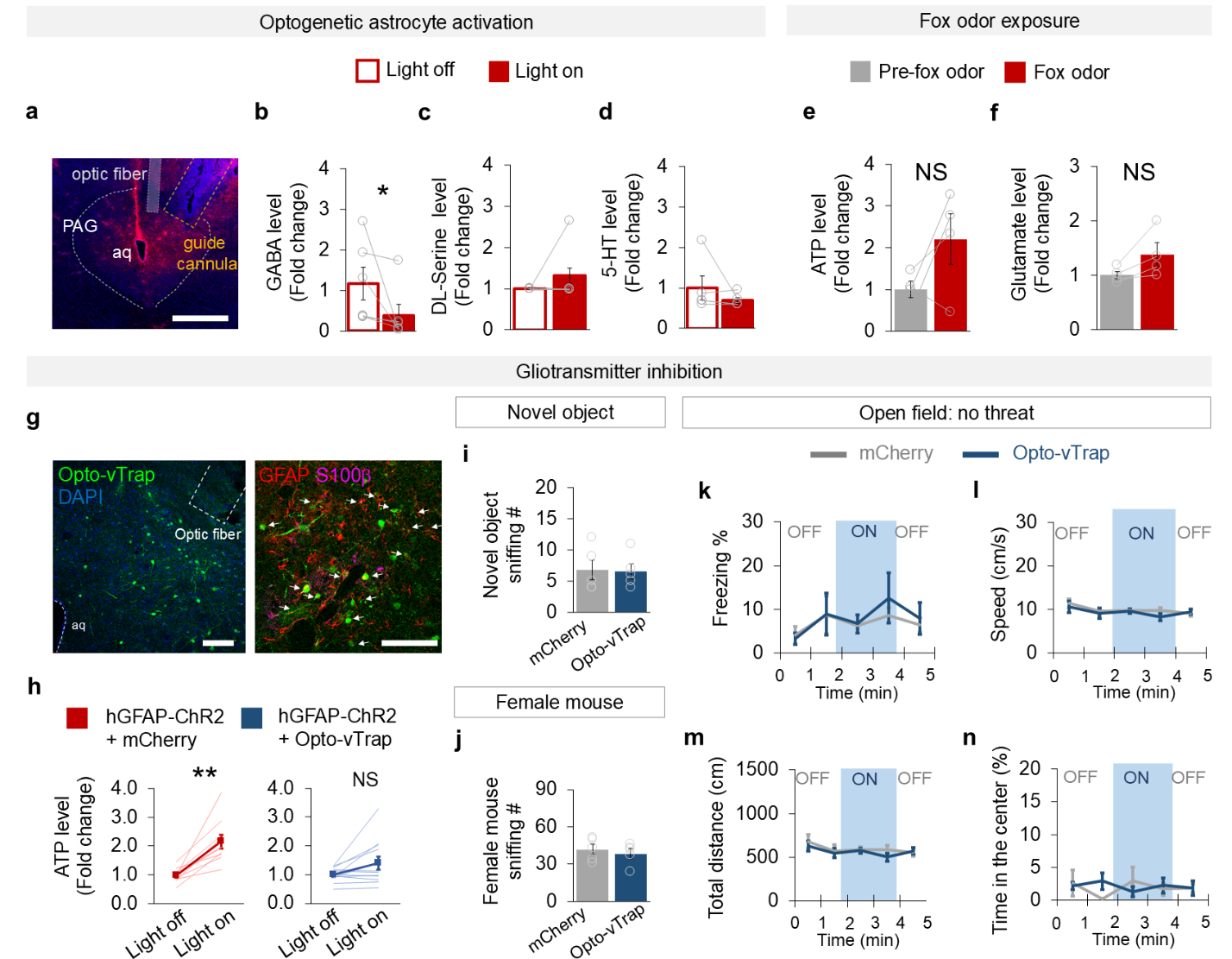


Supplementary Figure 13. Gliotransmitter release upon optogenetic dPAG astrocyte activation.

(a) Representative image of dPAG tissue implanted with a CMA7 microdialysis cannula (yellow broken line) and optic fiber for light stimulation (white line). Scale bar, 500 μm. (b) Fold change quantification of extracellular dPAG levels of GABA (n = 6 mice, Wilcoxon signed-rank test **p* = 0.028), (c) D/L-serine (Wilcoxon signed-rank test **p* = 0.686), and (d) 5-HT during light-off and light-on epochs (hGFAP-hChR2 n = 5 mice, Wilcoxon signed-rank test *p* = 0.257). (e) Fold change quantification of extracellular ATP (Wilcoxon signed-rank test *p* = 0.144) and (f) glutamate levels in the dPAG during mouse exposure to normal air (control) or fox odor (n = 4 mice, Wilcoxon signed-rank test *p* = 0.068). (g) Representative image of Opto-vTrap expression (green) in the dPAG with an optic fiber implant (left) and its colocalization with GFAP^+^ (red) and S100β^+^ (purple) astrocytes. White arrows indicate a merged signal. Scale bar, 100 μm (left), 200 μm (right). (h) Fold change quantification of the extracellular levels of ATP in dPAG slices from hGFAP-ChR2 mice expressing control virus (GFAP-mCherry n = 10 mice, red bar, Wilcoxon signed-rank test ***p* = 0.005) or Opto-vTrap (n = 12 mice, blue, Wilcoxon signed-rank test *p* = 0.051) during light-off and light-on epochs. (i) Quantification of the number of sniffing behavior/interactions upon exposure to non-threatening stimuli: a small novel object (n = 5 mice per group, two-tailed Mann-Whitney *U* -test *p* = 0.841) and (j) a female mouse conspecific (n = 5 mice per group, two-tailed Mann-Whitney *U* -test *p* = 0.548). (k) Percentage of time spent freezing (l) speed, (m) total distance traveled, and (n) percentage of time spent in the center by GFAP-mCherry- (n = 5 mice) and GFAP-Opto-vTrap-injected mice (n = 8 mice) during light-off and light-on epochs in the OFT (GFAP-mCherry n = 5 mice, GFAP-Opto-vTrap n = 8 mice, (Two-tailed Mann-Whitney *U* -test **p* < 0.05, NS, not significant). Data are presented as the mean ± s.e.m. See Supplementary table 1 for detailed values and statistics.


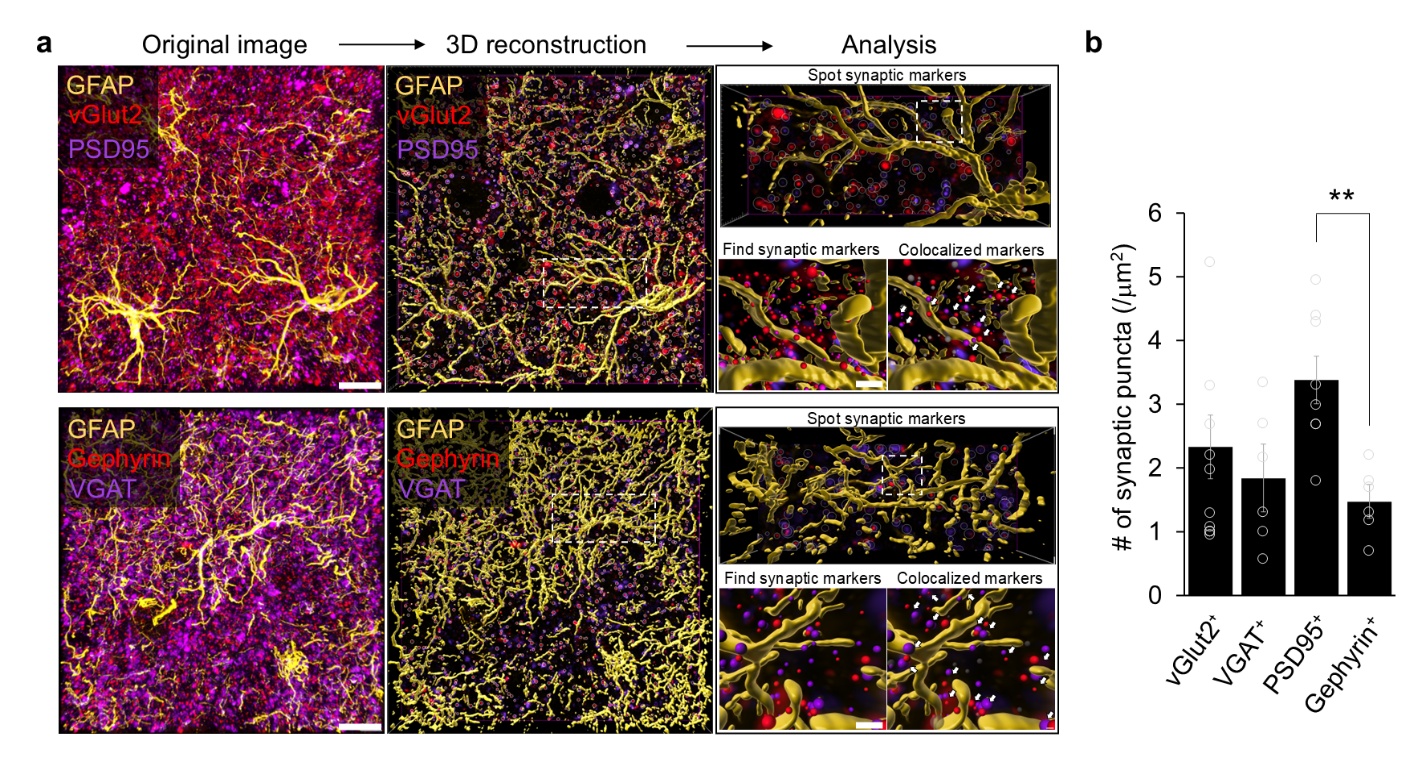


Supplementary Figure 14. Synaptic puncta surrounding astrocytes in PAG.

(a) Representative confocal and 3D-reconstructed images of PAG astrocytes (GFAP) and excitatory and inhibitory pre- and post-synaptic puncta (vGlut2: excitatory presynaptic; PSD95: excitatory postsynaptic; VGAT: inhibitory presynaptic; Gephyrin: inhibitory postsynaptic). Scale bar, 5 μm and 10 μm. (b) Quantification of synaptic puncta co-localized with astrocyte processes. (Two-tailed Mann-Whitney *U*-test, ***p* < 0.01). Data are presented as the mean ± s.e.m. See Supplementary table 1 for detailed values and statistics.


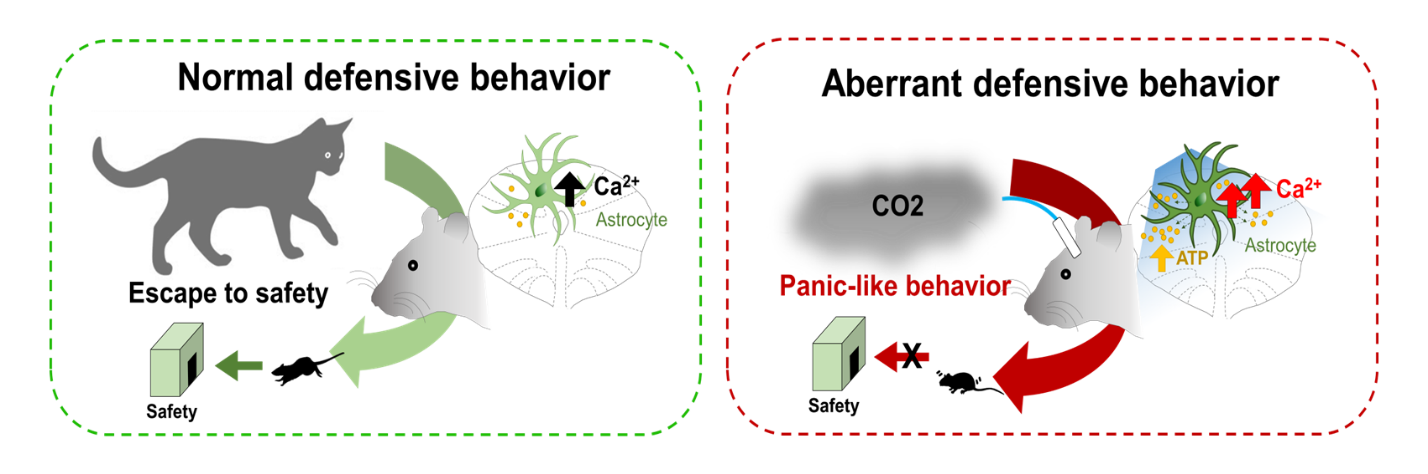


Supplementary Figure 15. Graphical summary.

PAG astrocyte Ca^2+^ activity increases upon threat exposure that triggers animals to execute defensive response. Meanwhile, distinct increases of PAG astrocyte Ca^2+^ activity and ATP release

in a CO_2_-induced panic-like state where mice failed to execute a safety-directed escape. This study demonstrates an active role of PAG astrocyte in mouse defensive and panic-like behaviors through distinct Ca^2+^ activity and ATP release.

Supplementary Table

**Supplementary table 1.** Statistical analysis.

| **Figure** | **Statistical test** | **Sample size** | **Values** |
| --- | --- | --- | --- |
| Fig. 1a | Two-tailed paired t-test | 4 mice | *p* = 0.002 (Baseline vs Fox odor) |
| Fig. 1b | Two-tailed paired t-test | 4 mice | *p* = 0.009 (Baseline vs Fox odor at 0s) |
| Fig. 1c | Two-tailed paired t-test | 5 mice | *p* < 0.001 (Baseline vs Attack) |
| Fig. 1d | Two-tailed paired t-test | 5 mice | *p* = 0.012 (Baseline vs Attack at 0 s) |
| Fig. 1e | Two-tailed paired t-test | 5 mice | Risk assessment  *p* = 0.043 (Pre vs During)  Flight speed  *p* = 0.078 (Pre vs Attack) |
| Fig. 1f | Two-tailed paired t-test | 5 mice | *p* < 0.001 (Baseline vs Approach at 0 s)  *p* < 0.001 (Baseline vs Attack at 0 s) |
| Fig. 1g | Two-tailed paired t-test | 4 mice | *p* < 0.001 (Pre vs During) |
| Fig. 1h | Two-tailed paired t-test | 4 mice | *p* = 0.437 (Baseline vs Female mouse at 0 s) |
| Fig. 1i | Two-tailed paired t-test | 4 mice | *p* = 0.001 (Pre vs During) |
| Fig. 1j | Two-tailed paired t-test | 4 mice | *p* = 0.959 (Baseline vs Novel object at 0 s) |
| Fig. 1n | Two-tailed paired t-test | 38 cells | *p* < 0.0001 |
| Fig. 2a | Two-tailed Mann-Whitney *U* -test | 4 mice per group | *U* = 0.000, *p* = 0.021 (PBS vs L-AAA) |
| Fig. 2c | Two-tailed Mann-Whitney *U* -test | 5 mice in PBS  4 mice in L-AAA | *U* = 9.000, *p* = 0.806 (PBS vs L-AAA) |
| Fig. 2e | Two-way repeated measure ANOVA and multivariate test (Wilks’ lambda) followed by post hoc analysis (LSD) | 18 in PBS  11 in L-AAA | Wilks’ lambda = 0.040  F(3,8) = 63.637  *p* < 0.001 (Pre-tone vs Tone)  Tone  *p* = 0.258 (PBS vs L-AAA) |
| Figure 2e  Tone | Two-tailed Mann-Whitney *U* -test | 18 in PBS  11 in L-AAA | *U =* 52.000 *p* = 0.035 (PBS vs L-AAA) |
| Fig. 2f | Two-way repeated ANOVA and multivariate test (Wilks’ lambda) followed by post hoc analysis (LSD) | 6 mice per group | Wilks’ lambda = 0.075  F(3, 3) = 12.420  *p* = 0.034 (Before vs During fox odor)  During fox odor  *p* = 0.080 (PBS vs L-AAA) |
| Fig. 2f  During fox odor | Two-tailed Mann-Whitney *U* -test | 6 mice per group | *U* = 4.000, *p* = 0.025 (PBS vs L-AAA) |
| Fig. 2g | Two-tailed Mann-Whitney *U* -test | 6 mice per group | *U* = 11.000, *p* = 0.262 (PBS vs L-AAA) |
| Fig. 2h | Two-tailed Mann-Whitney *U* -test | 5 mice per group | *U* = 6.000, *p* = 0.055 (PBS vs L-AAA) |
| Fig. 2i | Two-tailed Mann-Whitney *U* -test | 7 mice in PBS  8 mice in L-AAA | *U* = 14.000, *p* = 0.105 (PBS vs L-AAA) |
| Fig. 2j | Two-tailed Mann-Whitney *U* -test | 6 mice per group | *U* = 9.000, *p* = 0.150 (PBS vs L-AAA) |
| Fig. 2k | Two-tailed Mann-Whitney *U* -test | 6 mice per group | *U* = 13.000, *p* = 0.423 (PBS vs L-AAA) |
| Fig. 2l | Two-tailed Mann-Whitney *U* -test | 8 mice in PBS  7 mice in L-AAA | *U* = 25.000, *p* = 0.728 (PBS vs L-AAA) |
| Fig. 2m | Two-tailed Mann-Whitney *U* -test | 8 mice in PBS  7 mice in L-AAA | *U* = 22.000, *p* = 0.487 (PBS vs L-AAA) |
| Fig. 2o | Two-way repeated ANOVA and multivariate test (Wilks’ lambda) followed by Two-tailed Mann-Whitney *U* -test | 5 mice per group | Wilks’ lambda = 0.011  F(3, 2) = 61.695  *p* = 0.016 (Before vs During fox odor)  During fox odor  *U =* 1.000, *p* = 0.016 (mCherry vs hPMCA2w/b) |
| Fig. 2p | Two-tailed Mann-Whitney *U* -test | 5 mice per group | *U =* 3.000, *p* = 0.047 (mCherry vs hPMCA2w/b) |
| Fig. 2q | Two-tailed Mann-Whitney *U* -test | 5 mice per group | *U =* 1.000, *p* = 0.016 (mCherry vs hPMCA2w/b) |
| Fig. 2r | Two-tailed Mann-Whitney *U* -test | 5 mice per group | Pre hand attack  *U =* 10.000, *p* = 0.690 (mCherry vs hPMCA2w/b)  Hand attack  *U =* 3.000, *p* = 0.047 (mCherry vs hPMCA2w/b) |
| Fig. 2s | Two-tailed Mann-Whitney *U* -test | 5 mice per group | *U =* 9.000, *p* = 0.548 (mCherry vs hPMCA2w/b) |
| Fig. 2t | Two-tailed Mann-Whitney *U* -test | 5 mice per group | *U =* 12.000, *p* = 0.917 (mCherry vs hPMCA2w/b) |
| Fig. 2u | Two-tailed Mann-Whitney *U* -test | 5 mice per group | *U =* 9.000, *p* = 0.465 (mCherry vs hPMCA2w/b) |
| Fig. 2v | Two-tailed Mann-Whitney *U* -test | 5 mice per group | *U =* 9.000, *p* = 0.465 (mCherry vs hPMCA2w/b) |
| Fig. 2w | Two-tailed Mann-Whitney *U* -test | 5 mice per group | *U =* 4.000, *p* = 0.075 (mCherry vs hPMCA2w/b) |
| Fig. 3c  No. of freezing events | Two-tailed Mann-Whitney *U* -test | 16 in Control  11 in hGFAP-ChR2 | *U =* 78.500, *p* = 0.390 (1 min)  *U =* 59.000, *p* = 0.063 (2 min)  *U =* 0.000, *p* < 0.001 (3 min)  *U =* 48.000*, p* = 0.043 (4 min)  *U =* 10.000, *p* = 0.000 (5 min)  *U =* 80.000, *p* = 0.228 (6 min)  *U =* 5.500*, p* < 0.001 (7 min)  *U =* 0.000, *p* < 0.001 (8 min)  *U =* 0.000*, p* < 0.001 (9 min)  *U =* 80.000, *p* = 0.228 (10 min) |
| Fig. 3c  Total no. of freezing events | Two-tailed Mann-Whitney *U* -test | 16 in Control  11 in hGFAP-ChR2 | *U =* 2.000, *p* < 0.001 (Control vs hGFAP-ChR2) |
| Fig. 3d  Freezing time | Two-tailed Mann-Whitney *U* -test | 16 in Control  11 in hGFAP-ChR2 | *U =* 78.500, *p* = 0.390 (1 min)  *U =* 60.000, *p* = 0.073 (2 min)  *U =* 0.000, *p* < 0.001 (3 min)  *U =* 0.000*, p* = 0.001 (4 min)  *U =* 12.000, *p* < 0.001 (5 min)  *U =* 80.000, *p* = 0.228 (6 min)  *U =* 0.000*, p* < 0.001 (7 min)  *U =* 0.000, *p* < 0.001 (8 min)  *U =* 0.000*, p* < 0.001 (9 min)  *U =* 80.000, *p* = 0.228 (10 min) |
| Fig. 3d  Total freezing time | Two-tailed Mann-Whitney *U* -test | 16 in Control  11 in hGFAP-ChR2 | *U =* 0.000*, p* < 0.001 (Control vs hGFAP-ChR2) |
| Fig. 3e  No. of fleeing | Two-tailed Mann-Whitney *U* -test | 16 in Control  11 in hGFAP-ChR2 | *U =* 80.000, *p* = 1.000 (1 min)  *U =* 82.500, *p* = 0.407 (2 min)  *U =* 80.000, *p* = 0.228 (3 min)  *U =* 85.000*, p* = 0.786 (4 min)  *U =* 88.000, *p* = 1.000 (5 min)  *U =* 88.000, *p* = 1.000 (6 min)  *U =* 68.000*, p* = 0.110 (7 min)  *U =* 85.000, *p* = 0.786 (8 min)  *U =* 88.000*, p* = 1.000 (9 min)  *U =* 88.000, *p* = 1.000 (10 min) |
| Fig. 3e  Total no. of fleeing | Two-tailed Mann-Whitney *U* -test | 16 in Control  11 in hGFAP-ChR2 | *U =* 80.000, *p* = 0.608 (Control vs hGFAP-ChR2) |
| Fig. 3f  Fleeing time | Two-tailed Mann-Whitney *U* -test | 16 in Control  11 in hGFAP-ChR2 | *U =* 88.000, *p* = 1.000 (1 min)  *U =* 82.500, *p* = 0.407 (2 min)  *U =* 80.000, *p* = 0.228 (3 min)  *U =* 80.000*, p* = 0.228 (4 min)  *U =* 88.000, *p* = 1.000 (5 min)  *U =* 77.000, *p* = 0.232 (6 min)  *U =* 64.000*, p* = 0.030 (7 min)  *U =* 85.000, *p* = 0.745 (8 min)  *U =* 88.000*, p* = 1.000 (9 min)  *U =* 88.000, *p* = 1.000 (10 min) |
| Fig. 3f  Total fleeing time | Two-tailed Mann-Whitney *U* -test | 16 in Control  11 in hGFAP-ChR2 | *U =* 68.000, *p* = 0.110 (Control vs hGFAP-ChR2) |
| Fig. 3g  No. of retreat | Two-tailed Mann-Whitney *U* -test | 16 in Control  11 in hGFAP-ChR2 | *U =* 77.000, *p* = 1.000 (1 min)  *U =* 80.000, *p* = 0.407 (2 min)  *U =* 40.000, *p* = 0.228 (3 min)  *U =* 68.000*, p* = 0.228 (4 min)  *U =* 64.000, *p* = 1.000 (5 min)  *U =* 88.000, *p* = 0.232 (6 min)  *U =* 40.000*, p* = 0.030 (7 min)  *U =* 39.500, *p* = 0.745 (8 min)  *U =* 40.000*, p* = 1.000 (9 min)  *U =* 88.000, *p* = 1.000 (10 min) |
| Fig. 3g  Total no. of retreat | Two-tailed Mann-Whitney *U* -test | 16 in Control  11 in hGFAP-ChR2 | *U =* 16.000, *p* < 0.001 (Control vs hGFAP-ChR2) |
| Fig. 3h  Retreat time | Two-tailed Mann-Whitney *U* -test | 16 in Control  11 in hGFAP-ChR2 | *U =* 77.000, *p* = 0.232 (1 min)  *U =* 88.000, *p* = 1.000 (2 min)  *U =* 40.000, *p* = 0.001 (3 min)  *U =* 68.000, *p* = 0.110 (4 min)  *U =* 64.000, *p* = 0.030 (5 min)  *U =* 88.000, *p* = 1.000 (6 min)  *U =* 48.000, *p* = 0.004 (7 min)  *U =* 40.000, *p* = 0.005 (8 min)  *U =* 48.000, *p* = 0.004 (9 min)  *U =* 88.000, *p* = 1.000 (10 min) |
| Fig. 3h  Total retreat time | Two-tailed Mann-Whitney *U* -test | 16 in Control  11 in hGFAP-ChR2 | *U =*19.500 , *p* < 0.001 (Control vs hGFAP-ChR2) |
| Fig. 3i  No. of tail rattling | Two-tailed Mann-Whitney *U* -test | 16 in Control  11 in hGFAP-ChR2 | *U =* 88.000, *p* = 1.000 (1 min)  *U =* 82.500, *p* = 0.407 (2 min)  *U =* 40.000, *p* = 0.001 (3 min)  *U =* 56.000, *p* = 0.011 (4 min)  *U =* 72.000, *p* = 0.082 (5 min)  *U =* 88.000, *p* = 1.000 (6 min)  *U =* 40.000, *p* = 0.001 (7 min)  *U =* 48.000, *p* = 0.004 (8 min)  *U =* 64.000, *p* = 0.030 (9 min)  *U =* 88.000, *p* = 1.000 (10 min) |
| Fig. 3i  Total no. of tail rattling | Two-tailed Mann-Whitney *U* -test | 16 in Control  11 in hGFAP-ChR2 | *U =* 8.500, *p* < 0.001 (Control vs hGFAP-ChR2) |
| Fig. 3j  Tail rattling time | Two-tailed Mann-Whitney *U* -test | 16 in Control  11 in hGFAP-ChR2 | *U =* 88.000, *p* = 1.000 (1 min)  *U =* 82.500, *p* = 0.407 (2 min)  *U =* 40.000, *p* = 0.001 (3 min)  *U =* 56.000, *p* = 0.011 (4 min)  *U =* 72.000, *p* = 0.082 (5 min)  *U =* 88.000, *p* = 1.000 (6 min)  *U =* 40.000, *p* = 0.001 (7 min)  *U =* 48.000, *p* = 0.004 (8 min)  *U =* 64.000, *p* = 0.030 (9 min)  *U =* 88.000, *p* = 1.000 (10 min) |
| Fig. 3j  Total tail rattling time | Two-tailed Mann-Whitney *U* -test | 16 in Control  11 in hGFAP-ChR2 | *U =* 8.500, *p* < 0.001 (Control vs hGFAP-ChR2) |
| Fig. 4b | Wilcoxon signed rank test | 9 in Control  5 in hGFAP-ChR2 | Control  *p* = 0.465 (off vs on)  hGFAP-ChR2  *p* = 0.035 (off vs on) |
| Fig. 4b  Light on | Two-tailed Mann-Whitney *U* -test | 9 in Control  5 in hGFAP-ChR2 | *U =* 0.000, *p* = 0.002 (Control vs hGFAP-ChR2) |
| Fig. 4c | Wilcoxon signed rank test | 9 in Control  5 in hGFAP-ChR2 | Control  *p* = 0.859 (off vs on)  hGFAP-ChR2  *p* = 0.043 (off vs on) |
| Fig. 4c  Light on | Two-tailed Mann-Whitney *U* -test | 9 in Control  5 in hGFAP-ChR2 | *U =* 0.000 *p* = 0.003 (Control vs hGFAP-ChR2) |
| Fig. 4d | Two-tailed Mann-Whitney *U* -test | 9 mice in Control  5 mice in hGFAP-ChR2 | *U =* 0.000, *p* < 0.001 (Control vs hGFAP-ChR2) |
| Fig. 4e | Two-tailed Mann-Whitney *U* -test | 9 mice in Control  5 mice in hGFAP-ChR2 | *U =* 18.000, *p* = 0.180 (Control vs hGFAP-ChR2) |
| Fig. 4f | Two-tailed Mann-Whitney *U* -test | 9 mice in Control  5 mice in hGFAP-ChR2 | *U =* 8.500, *p* = 0.039 (Control vs hGFAP-ChR2) |
| Fig. 4g | Two-tailed Mann-Whitney *U* -test | 9 mice in Control  5 mice in hGFAP-ChR2 | *U =* 10.000, *p* = 0.037 (Control vs hGFAP-ChR2) |
| Fig. 4i | One-way ANOVA and post-hoc analysis for multiple comparison (LSD) | 8 in hSyn-oChIEF  6 in hSyn-oChIEF + GFAP-ChR2 | hSyn-oChIEF  *p* < 0.001  F(2, 21) = 22.391  *p* < 0.001 (OFF vs 40 Hz)  *p* < 0.001 (OFF vs Constant)  *p* = 0.988 (40 Hz vs Constant)  hSyn-oChIEF + hGFAP-ChR2  *p* = 0.796  F(2, 15) = 0.231  *p* = 0.810 (OFF vs 40 Hz)  *p* = 0.676 (OFF vs Constant)  *p* = 0.152 (40 Hz vs Constant) |
| Fig. 4i  hSyn-oChIEF vs  hSyn-oChIEF + hGFAP-ChR2 | Wilcoxon signed rank test | 8 in hSyn-oChIEF  6 in hSyn-oChIEF + GFAP-ChR2 | *p* = 0.655 (OFF)  *p* = 0.028 (40 Hz)  *p* = 0.046 (Constant) |
| Fig. 4j | One-way ANOVA and post-hoc analysis for multiple comparison (LSD) | 8 in hSyn-oChIEF  6 in hSyn-oChIEF + GFAP-ChR2 | hSyn-oChIEF  *p* < 0.001  F(2, 18) = 12.106  *p* < 0.001 (OFF vs 40 Hz)  *p* < 0.001 (OFF vs Constant)  *p* = 0.988 (40 Hz vs Constant)  hSyn-oChIEF + hGFAP-ChR2  *p* = 0.035  F(2, 24) = 3.856  *p* = 0.733 (OFF vs 40 Hz)  *p* = 0.037 (OFF vs Constant)  *p* = 0.017 (40 Hz vs Constant) |
| Fig. 4j  hSyn-oChIEF vs  hSyn-oChIEF + hGFAP-ChR2 | Wilcoxon signed rank test | 8 in hSyn-oChIEF  6 in hSyn-oChIEF + GFAP-ChR2 | *p* = 0.028 (OFF)  *p* < 0.018 (40 Hz)  *p* < 0.018 (Constant) |
| Fig. 4k | One-way ANOVA and post-hoc analysis for multiple comparison (LSD) | 8 in hSyn-oChIEF  6 in hSyn-oChIEF + GFAP-ChR2 | hSyn-oChIEF  *p* = 0.704  F(2, 18) = 0.358  *p* = 0.435 (OFF vs 40 Hz)  *p* = 0.529 (OFF vs Constant)  *p* = 0.877 (40 Hz vs Constant)  hSyn-oChIEF + hGFAP-ChR2  *p* < 0.001  F(2, 24) = 147.262  *p* < 0.001 (OFF vs 40 Hz)  *p* < 0.001 (OFF vs Constant)  *p* = 0.784 (40 Hz vs Constant) |
| Fig. 4k  hSyn-oChIEF vs  hSyn-oChIEF + hGFAP-ChR2 | Wilcoxon signed rank test | 8 in hSyn-oChIEF  6 in hSyn-oChIEF + GFAP-ChR2 | *p* = 0.917 (OFF)  *p* = 0.028 (40 Hz)  *p* < 0.018 (Constant) |
| Fig. 4l | One-way ANOVA and post-hoc analysis for multiple comparison (LSD) | 8 in hSyn-oChIEF  6 in hSyn-oChIEF + GFAP-ChR2 | hSyn-oChIEF  *p* < 0.001  F(2, 18) = 12.455  *p* = 0.001 (OFF vs 40 Hz)  *p* < 0.001 (OFF vs Constant)  *p* = 0.370 (40 Hz vs Constant)  hSyn-oChIEF + hGFAP-ChR2  *p* < 0.001  F(2, 24) = 28.790  *p* < 0.001 (OFF vs 40 Hz)  *p* < 0.001 (OFF vs Constant)  *p* = 0.393 (40 Hz vs Constant) |
| Fig. 4l  hSyn-oChIEF vs  hSyn-oChIEF + hGFAP-ChR2 | Wilcoxon signed rank test | 8 in hSyn-oChIEF  6 in hSyn-oChIEF + GFAP-ChR2 | *p* = 0.735 (OFF)  *p* = 0.128 (40 Hz)  *p* = 0.176 (Constant) |
| Fig. 4o | One-way ANOVA and post-hoc analysis for multiple comparison (LSD) | 8 in each group | CamKII-ChR2  *p* = 0.0003  F(2, 21) = 11.94  *p* < 0.001 (OFF vs 40 Hz)  *p* < 0.001 (OFF vs Constant)  *p* = 0.972 (40 Hz vs Constant)  CamKII-ChR2  + hGFAP-ChR2  *p* = 0.031  F(2, 21) = 4.1  *p* = 0.850 (OFF vs 40 Hz)  *p* = 0.018 (OFF vs Constant)  *p* = 0.027 (40 Hz vs Constant) |
| Fig. 4o  CamKII-ChR2  vs  CamKII-ChR2  + hGFAP-ChR2 | Wilcoxon signed rank test | 8 in each group | *p* = 0.547 (OFF)  *p* = 0.0156 (40 Hz)  *p* = 0.0078 (Constant) |
| Fig. 4p | One-way ANOVA and post-hoc analysis for multiple comparison (LSD) | 8 in each group | CamKII-ChR2  *p* = 0.205  F(2, 21) = 1.71  *p* = 0.114 (OFF vs 40 Hz)  *p* = 0.927 (OFF vs Constant)  *p* = 0.135 (40 Hz vs Constant)  CamKII-ChR2  + hGFAP-ChR2  *p* < 0.0001  F(2, 21) = 125.65  *p* < 0.0001 (OFF vs 40 Hz)  *p* < 0.0001 (OFF vs Constant)  *p* = 0.279 (40 Hz vs Constant) |
| Fig. 4p  CamKII-ChR2  vs  CamKII-ChR2  + hGFAP-ChR2 | Wilcoxon signed rank test | 8 in each group | *p* = 1 (OFF)  *p* = 0.0078 (40 Hz)  *p* = 0.0078 (Constant) |
| Fig. 4s | One-way ANOVA and post-hoc analysis for multiple comparison (LSD) | 8 in each group | mDlx-ChR2  *p* = 0.732  F(2, 21) = 0.32  *p* = 0.452 (OFF vs 40 Hz)  *p* = 0.844 (OFF vs Constant)  *p* = 0.577 (40 Hz vs Constant)  mDlx-ChR2 + hGFAP-ChR2  *p* = 0.0239  F(2, 21) = 4.48  *p* = 0.402 (OFF vs 40 Hz)  *p* = 0.0083 (OFF vs Constant)  *p* = 0.0523 (40 Hz vs Constant) |
| Fig. 4s  mDlx-ChR2  vs  mDlx-ChR2  + hGFAP-ChR2 | Wilcoxon signed rank test | 8 in each group | *p* = 0.382 (OFF)  *p* = 0.0781 (40 Hz)  *p* = 0.0156 (Constant) |
| Fig. 4t | One-way ANOVA and post-hoc analysis for multiple comparison (LSD) | 8 in each group | mDlx-ChR2  *p* = 0.598  F(2, 21) = 0.53  *p* = 0.329 (OFF vs 40 Hz)  *p* = 0.487 (OFF vs Constant)  *p* = 0.774 (40 Hz vs Constant)  mDlx-ChR2 + hGFAP-ChR2  *p* < 0.0001  F(2, 21) = 71.7  *p* < 0.0001 (OFF vs 40 Hz)  *p* < 0.0001 (OFF vs Constant)  *p* = 0.530 (40 Hz vs Constant) |
| Fig. 4t  mDlx-ChR2  vs  mDlx-ChR2  + hGFAP-ChR2 | Wilcoxon signed rank test | 8 in each group | *p* = 0.500 (OFF)  *p* = 0.0078 (40 Hz)  *p* = 0.0078 (Constant) |
| Fig. 5b  Hot plate | Wilcoxon signed-rank test | 5 mice | *p* = 0.042 (Climb vs Jumps) |
| Fig. 5b  CO_2_ | Wilcoxon signed-rank test | 10 mice | *p* = 0.011 (Climbs vs Jumps) |
| Fig. 5b  Fox odor | Wilcoxon signed-rank test | 8 mice | *p* = 0.0078 (Climbs vs Jumps) |
| Fig. 5c | One-way ANOVA and post-hoc analysis for multiple comparison (LSD) | 5 mice in Hot plate  5 mice in CO_2_  8 mice in Fox odor | *p* < 0.0001  F(2, 15) = 97.35  *p* < 0.0001 (Hot plate vs CO_2_)  *p* < 0.0001 (Hot plate vs Fox odor)  *p* = 0.006 (CO_2_ vs Fox odor) |
| Fig. 5d | One-way ANOVA and post-hoc analysis for multiple comparison (LSD) | 5 mice in Hot plate  5 mice in CO_2_  8 mice in Fox odor | *p* < 0.0001  F(2, 15) = 52.77  *p* < 0.0001 (Hot plate vs CO_2_)  *p* < 0.0001 (Hot plate vs Fox odor)  *p* = 0.0002 (CO_2_ vs Fox odor) |
| Fig. 5e | Wilcoxon signed-rank test | 8 mice | *p* = 0.012 (Air vs CO_2_) |
| Fig. 5f | Wilcoxon signed-rank test | 8 mice | *p* = 0.012 (Air vs CO_2_) |
| Fig. 5g | Wilcoxon signed-rank test | 8 mice | *p* = 0.012 (Air vs CO_2_) |
| Fig. 5h | Wilcoxon signed-rank test | 8 mice | *p* = 0.0078 (Air vs Fox odor) |
| Fig. 5i | Wilcoxon signed-rank test | 8 mice | *p* = 0.0195 (Air vs Fox odor) |
| Fig. 5k  PBS | Wilcoxon signed-rank test | 7 mice | *p* = 0.018 (Climbs vs Jumps) |
| Fig. 5k  L-AAA | Wilcoxon signed-rank test | 7 mice | *p* = 0.655 (Climbs vs Jumps) |
| Fig. 5k  Jumps | Two-tailed Mann-Whitney *U* -test | 7 mice per group | *U* = 0.000, *p* = 0.001 (PBS vs L-AAA) |
| Fig. 5l | Two-tailed Mann-Whitney *U* -test | 5 mice per group | *U* = 8.000, *p* = 0.347 (PBS vs L-AAA) |
| Fig. 5m | Two-tailed Mann-Whitney *U* -test | 5 mice per group | *U* = 0.000, *p* = 0.009 (PBS vs L-AAA) |
| Fig. 5n  PBS | Wilcoxon signed-rank test | 6 mice | *p* = 0.032 (Climbs vs Jumps) |
| Fig. 5n  L-AAA | Wilcoxon signed-rank test | 6 mice | *p* = 0.500 (Climbs vs Jumps) |
| Fig. 5n  Jumps | Two-tailed Mann-Whitney *U* -test | 6 mice per group | *U* = 0.000, *p* = 0.0022 (PBS vs L-AAA) |
| Fig. 5o | Two-tailed Mann-Whitney *U* -test | 6 mice per group | *U* = 12.0, *p* = 0.394 (PBS vs L-AAA) |
| Fig. 5p | Two-tailed Mann-Whitney *U* -test | 6 mice per group | *U* = 0.000, *p* = 0.0022 (PBS vs L-AAA) |
| Fig. 5r | Two-tailed Mann-Whitney *U* -test | 5 mice per group | *U* = 3.500, *p* = 0.052 (mCherry vs hPMCA2w/b) |
| Fig. 5s | Two-tailed Mann-Whitney *U* -test | 5 mice per group | *U* = 3.500, *p* = 0.041 (mCherry vs hPMCA2w/b) |
| Fig. 5t | Two-tailed Mann-Whitney *U* -test | 6 mice per group | *U* = 0.000, *p* = 0.0022 (mCherry vs hPMCA2w/b) |
| Fig. 5u | Two-tailed Mann-Whitney *U* -test | 6 mice per group | *U* = 0.000, *p* = 0.0022 (mCherry vs hPMCA2w/b) |
| Fig. 6a | Two-tailed paired t-test | 3 mice | GRAB ATP1.0  *p* = 0.043 (pre vs CO_2_)  EGFP  *p* = 0.578 (pre vs CO_2_) |
| Fig. 6b | Wilcoxon signed-rank test | 10 mice | *p* = 0.013 (Light off vs Light on) |
| Fig. 6c | Wilcoxon signed-rank test | 6 mice | *p* = 0.799 (Light off vs Light on) |
| Fig. 6e | Two-tailed Mann-Whitney *U* -test | 10 mice in hGFAP-ChR2 + mCherry  12 in hGFAP-ChR2 + Opto-vTrap | *U* = 26.000, *p* = 0.025 (hGFAP-ChR2 + mCherry vs hGFAP-ChR2 + Opto-vTrap |
| Fig. 6g | Two-tailed Mann-Whitney *U* -test | 5 mice per group | *U* = 0.000, *p* = 0.008 (GFAP + mCherry vs GFAP + Opto-vTrap) |
| Fig. 6h | Two-tailed Mann-Whitney *U* -test | 5 mice per group | Before  *U* = 9.000, *p* = 0.465 (mCherry vs Opto-vTrap)  During  *U* = 0.000, *p* = 0.009 (mCherry vs Opto-vTrap) |
| Fig. 6i | Two-tailed Mann-Whitney *U* -test | 5 mice per group | *U* = 5.000, *p* = 0.028 (mCherry vs Opto-vTrap) |
| Fig. 6j | Two-tailed Mann-Whitney *U* -test | 5 mice in mCherry  8 mice in Opto-vTrap | *U* = 5.000, *p* = 0.028 (mCherry vs Opto-vTrap) |
| Fig. 6k | Two-tailed Mann-Whitney *U* -test | 5 mice in GFAP-mCherry  8 mice in GFAP-Opto-vTrap | *U* = 5.000, *p* = 0.047 (mCherry vs Opto-vTrap) |
| Fig. 6m  No. of freezing events | Two-tailed Mann-Whitney *U* -test | 6 in each group | *U =* 18.00, *p* = 1 (1 min)  *U =* 15.00, *p* = 1 (2 min)  *U =* 7, *p =* 0.0974 (3 min)  *U =* 3*, p* = 0.0152 (4 min)  *U =* 9, *p* = 0.232 (5 min)  *U =* 9, *p* = 0.182 (6 min)  *U =* 7.5*, p* = 0.104 (7 min)  *U =* 1.5, *p* = 0.0065 (8 min)  *U =* 4.5*, p* = 0.0411 (9 min)  *U =* 9, *p* = 0.182 (10 min) |
| Fig. 6m  Total no. of freezing events | Two-tailed Mann-Whitney *U* -test | 6 in each group | *U =* 0.000*, p* = 0.0022 (PBS vs PPADS) |
| Fig. 6n  Freezing time | Two-tailed Mann-Whitney *U* -test | 6 in each group | *U =* 18.00, *p* = 1 (1 min)  *U =* 15.00, *p* = 1 (2 min)  *U =* 1, *p =* 0.0043 (3 min)  *U =* 1*, p* = 0.0043 (4 min)  *U =* 1, *p* = 0.0043 (5 min)  *U =* 9, *p* = 0.182 (6 min)  *U =* 0.000*, p* < 0.0001 (7 min)  *U =* 1.5, *p* < 0.0001 (8 min)  *U =* 10*, p* = 0.210 (9 min)  *U =* 6, *p* = 0.061 (10 min) |
| Fig. 6n  Total freezing time | Two-tailed Mann-Whitney *U* -test | 6 in each group | *U =* 0.000*, p* < 0.0001 (PBS vs PPADS) |
| Fig. 7c  mEPSC amplitude | Two-tailed paired t-test | 12 cells from 5 control mice (YFP)  14 cells from 6 GFAP-ChR2 mice | *p* = 0.5983 (Basal vs. Light in YFP group)  *p* = 0.00056 (Basal vs. Light in ChR2 group) |
| Fig. 7c  mEPSC frequency | Two-tailed paired t-test |  | *p* = 0.3807 (Basal vs. Light in YFP group)  *p* = 0.000057 (Basal vs. Light in ChR2 group) |
| Fig. 7d  mEPSC amplitude | One-way ANOVA and post-hoc analysis for multiple comparisons (LSD) | 13 cell from 7 mice | p < 0.0001  F(2, 36) = 11.74  *p* = 0.6497 (Basal vs PBS)  *p* < 0.0001 (Basal vs ATP)  *p* = 0.0009 (PBS vs ATP) |
| Fig. 7d  mEPSC frequency | One-way ANOVA and post-hoc analysis for multiple comparisons (LSD) |  | p < 0.0001  F(2, 36) = 58.82  *p* = 0.2878 (Basal vs PBS)  *p* < 0.0001 (Basal vs ATP)  *p* < 0.0001 (PBS vs ATP) |
| Fig. 7e  mEPSC amplitude | Two-tailed paired t-test | 10 cells from 4 control mice (YFP)  12 cells from 4 GFAP-ChR2 mice | *p* = 0.8180 (Basal vs. Light+PPADS in YFP group)  *p* = 0.1296 (Basal vs. Light+PPADS in ChR2 group) |
| Fig. 7e  mEPSC frequency | Two-tailed paired t-test |  | *p* = 0.7101 (Basal vs. Light+PPADS in YFP group)  *p* = 0.1022 (Basal vs. Light+PPADS in ChR2 group) |
| Fig. 7d  mEPSC frequency | Two-tailed paired t-test | 9 cells from 3 control mice (YFP)  12 cells from 5 GFAP-ChR2 mice | *p* = 0.841 (Basal vs. Light in YFP group)  *p* = 0.189 (Basal vs. Light in ChR2 group) |
| Fig. 7e  mEPSC amplitude | Two-tailed paired t-test |  | *p* = 0.287 (Basal vs. Light in YFP group)  *p* = 0.118 (Basal vs. Light in ChR2 group) |
| Fig. 7e  mEPSC frequency | One-way ANOVA and post-hoc analysis for multiple comparisons (LSD) | 11 cells from 5 mice | p = 0.130  F(2, 30) = 2.19  *p* = 0.129 (Basal vs PBS)  *p* = 0.672 (Basal vs ATP)  *p* = 0.096 (PBS vs ATP) |
| Fig. 7e  mEPSC amplitude | One-way ANOVA and post-hoc analysis for multiple comparisons (LSD) |  | *p* = 0.218  F(2, 30) = 1.6  *p* = 0.445 (Basal vs PBS)  *p* = 0.0845 (Basal vs ATP)  *p* = 0.320 (PBS vs ATP) |
| Supplementary fig. 1b | One-way ANOVA and post-hoc analysis for multiple comparisons (LSD) | 3 mice in Home cage  4 mice in Foot shock  3 mice in Fox odor  3 mice in Hand attack  3 mice in RC car attack  4 mice in Pain  4 mice in Novel object  4 mice in Female mouse  4 mice in Sucrose | *p* < 0.001  F(2, 23) = 47.931  *p* < 0.001 (Home cage vs Foot shock)  *p* = 0.009 (Home cage vs Fox odor)  *p* < 0.001 (Home cage vs Hand attack)  *p* < 0.001 (Home cage vs RC car attack)  *p* = 0.537 (Home cage vs Acute pain)  *p* = 0.934 (Home cage vs Novel object)  *p* = 0.976 (Home cage vs Female mouse)  *p* = 0.122 (Home cage vs Sucrose)  *p* < 0.001 (Foot shock vs Fox odor)  *p* = 0.031 (Foot shock vs Hand attack)  *p* = 0.026 (Foot shock vs RC car)  *p* < 0.001 (Foot shock vs Acute pain)  *p* < 0.001 (Foot shock vs Novel object)  *p* < 0.001 (Foot shock vs Female mouse)  *p* < 0.001 (Foot shock vs Sucrose)  *p* < 0.001 (Fox odor vs Hand attack)  *p* < 0.001 (Fox odor vs RC car)  *p* = 0.001 (Fox odor vs Acute pain)  *p* = 0.004 (Fox odor vs Novel object)  *p* = 0.005 (Fox odor vs Female mouse)  *p* < 0.001 (Fox odor vs Sucrose)  *p* = 0.935 (Hand attack vs RC car)  *p* < 0.001 (Hand attack vs Acute pain)  *p* < 0.001 (Hand attack vs Novel object)  *p* < 0.001 (Hand attack vs Female mouse)  *p* < 0.001 (Hand attack vs Female mouse)  *p* < 0.001 (Hand attack vs Sucrose)  *p* < 0.001 (RC car vs Acute pain)  *p* < 0.001 (RC car vs Novel object)  *p* < 0.001 (RC car vs Female mouse)  *p* < 0.001 (RC car vs Female mouse)  *p* < 0.001 (RC car vs Sucrose)  *p* = 0.563 (Acute pain vs Novel object)  *p* = 0.526 (Acute pain vs Female mouse)  *p* = 0.301 (Acute pain vs Sucrose)  *p* = 0.955 (Novel object vs Female mouse)  *p* = 0.114 (Novel object vs Sucrose)  *p* = 0.102 (Female mouse vs Sucrose) |
| Supplementary fig. 1e | Two-tailed Mann-Whitney *U* -test | 20 | *U* = 10.000, *p* < 0.0001 (CamKII vs GABA) |
| Supplementary fig. 2e | One-way repeated ANOVA | 3 mice | *p* = 0.787  F(2, 6) = 0.248 |
| Supplementary fig. 2f | One-way repeated ANOVA and post-hoc analysis for multiple comparisons (Bonferroni) | 3 mice | *p* = 0.007  F(2, 6) = 13.066  *p* = 0.007 (Dorsal vs. Lateral)  *p* = 0.161 (Dorsal vs. Ventrolateral)  *p* = 0.0288 (Lateral vs. Ventrolateral) |
| Supplementary fig. 4b | Two-tailed Mann-Whitney *U* -test | 4 in each group | *U* = 7, *p* = 0.886  (Iba1: PBS vs L-AAA)  *U* = 4, *p* = 0.343  (CC1: PBS vs L-AAA) |
| Supplementary fig. 4d | Two-tailed Mann-Whitney *U* -test | 6 mice per group | *U* = 11.000, *p* = 0.262  (PBS vs L-AAA) |
| Supplementary fig. 4e | Two-tailed Mann-Whitney *U* -test | 6 mice per group | *U* = 11.000, *p* = 0.262  (PBS vs L-AAA) |
| Supplementary fig. 4f | Two-tailed Mann-Whitney *U* -test | 6 mice per group | *U* = 10.000, *p* = 0.200  (PBS vs L-AAA) |
| Supplementary fig. 4g | Two-tailed Mann-Whitney *U* -test | 6 mice per group | *U* = 10.000, *p* = 0.262  (PBS vs L-AAA) |
| Supplementary fig. 4h | Two-tailed Mann-Whitney *U* -test | 6 mice per group | *U* = 10.000, *p* = 0.262  (PBS vs L-AAA) |
| Supplementary fig. 4g | Two-way repeated ANOVA and multivariate test (Wilks’ lambda) | 17 in PBS  13 in L-AAA | Wilks’ lambda = 0.194  F(1, 12) = 49.847  *p* < 0.001 (Baseline vs Context) |
| Supplementary fig. 4g Context | Two-tailed Mann-Whitney *U* -test | 17 in PBS  13 in L-AAA | *U* = 93.000, *p* = 0.464  (PBS vs L-AAA) |
| Supplementary fig. 4j | Two-tailed Mann-Whitney *U* -test | 7 in PBS  5 in L-AAA | *U* = 14.000, *p* = 0.570  (PBS vs L-AAA) |
| Supplementary fig. 4k | Two-tailed Mann-Whitney *U* -test | 7 in PBS  5 in L-AAA | *U* = 13.000, *p* = 0.465  (PBS vs L-AAA) |
| Supplementary fig. 4l | Two-tailed Mann-Whitney *U* -test | 7 in PBS  5 in L-AAA | *U* = 13.000, *p* = 0.465  (PBS vs L-AAA) |
| Supplementary fig. 4o | Two-way repeated ANOVA and multivariate test (Wilks’ lambda) followed by two-tailed Mann-Whitney *U*-test | 5 per group | Wilks’ lambda = 0.256  F(1, 8) = 23.242  *p* = 0.001 (Baseline vs Context)  Context  *p* = 0.917 (mCherry vs hPMCA2w/b) |
| Supplementary fig. 4p | Two-way repeated ANOVA and multivariate test (Wilks’ lambda) followed by two-tailed Mann-Whitney *U*-test | 5 per group | Wilks’ lambda = 0.329  F(1, 8) = 16.336  *p* = 0.004 (Baseline vs Context)  Tone  *p* = 0.465 (mCherry vs hPMCA2w/b) |
| Supplementary fig. 4q | Two-tailed Mann-Whitney *U* -test | 5 per group | *U* = 11.000, *p* = 0.754 (mCherry vs hPMCA2w/b) |
| Supplementary fig. 4r | Two-tailed Mann-Whitney *U* -test | 5 per group | *U* = 11.000, *p* = 0.754 (mCherry vs hPMCA2w/b) |
| Supplementary fig. 4s | Two-tailed Mann-Whitney *U* -test | 5 per group | *U* = 10.000, *p* = 0.602 (mCherry vs hPMCA2w/b) |
| Supplementary fig. 4t | Two-tailed Mann-Whitney *U* -test | 5 per group | *U* = 8.000, *p* = 0.347 (mCherry vs hPMCA2w/b) |
| Supplementary fig. 4u | Two-tailed Mann-Whitney *U* -test | 5 per group | *U* = 7.000, *p* = 0.251 (mCherry vs hPMCA2w/b) |
| Supplementary fig. 4v | Two-tailed Mann-Whitney *U* -test | 5 per group | *U* = 9.000, *p* = 0.465 (mCherry vs hPMCA2w/b) |
| Supplementary fig. 4w | Two-tailed Mann-Whitney *U* -test | 5 per group | *U* = 4.500, *p* = 0.172 (mCherry vs hPMCA2w/b) |
| Supplementary fig. 4x | Two-tailed Mann-Whitney *U* -test | 5 per group | *U* = 10.000, *p* = 0.093 (mCherry vs hPMCA2w/b) |
| Supplementary fig. 4y | Two-tailed Mann-Whitney *U* -test | 5 per group | *U* = 10.000, *p* = 0.597 (mCherry vs hPMCA2w/b) |
| Supplementary fig. 4z | Two-tailed Mann-Whitney *U* -test | 5 per group | *U* = 7.000, *p* = 0.251 (mCherry vs hPMCA2w/b) |
| Supplementary fig. 5c | One-way repeated ANOVA and post-hoc analysis for multiple comparisons (Bonferroni) | 3 mice | *p* < 0.001  F(2, 4) = 644.196  *p* = 0.001 (S100b^+^ vs NeuN^+^)  *p* = 0.002 (S100b^+^ vs Iba1^+^)  *p* = 0.290 (NeuN^+^ vs Iba1^+^) |
| Supplementary fig. 5k | Two-tailed Mann-Whitney *U* -test | 8 mice in Vehicle-injected (Control)  7 mice in Tamoxifen-injected (hGFAP-ChR2) | *U* = 26.000, *p* = 0.817 (mCherry vs Opto-vTrap) |
| Supplementary fig. 5l | Two-tailed Mann-Whitney *U* -test | 8 mice in Vehicle-injected (Control)  7 mice in Tamoxifen-injected (hGFAP-ChR2) | *U* = 20.000, *p* = 0.355 (Vehicle-injected (Control) vs Tamoxifen-injected (hGFAP-ChR2)) |
| Supplementary fig. 5m | Two-tailed Mann-Whitney *U* -test | 8 mice in Vehicle-injected (Control)  7 mice in Tamoxifen-injected (hGFAP-ChR2) | *U* = 18.500, *p* = 0.271 (Vehicle-injected (Control) vs Tamoxifen-injected (hGFAP-ChR2)) |
| Supplementary fig. 5n | Two-tailed Mann-Whitney *U* -test | 8 mice in Vehicle-injected (Control)  7 mice in Tamoxifen-injected (hGFAP-ChR2) | *U* = 25.000, *p* = 0.728 (Vehicle-injected (Control) vs Tamoxifen-injected (hGFAP-ChR2)) |
| Supplementary fig. 6f | Wilcoxon signed-rank test | 5 mice | *p* = 0.043 (Light off vs Light on) |
| Supplementary fig. 6h | Wilcoxon signed-rank test | 5 mice | *p* = 0.043 (Light off vs Light on) |
| Supplementary fig. 7b | Two-tailed Mann-Whitney *U* -test | 5 mice in Control  7 in hGFAP-ChR2 | *U* = 14.000, *p* = 0.570 (Control vs hGFAP-ChR2) |
| Supplementary fig. 7c | Two-tailed Mann-Whitney *U* -test | 5 mice in Control  7 in hGFAP-ChR2 | *U* = 10.000, *p* = 0.223 (Control vs hGFAP-ChR2) |
| Supplementary fig. 7d | Two-tailed Mann-Whitney *U* -test | 5 mice in Control  8 in hGFAP-ChR2 | *U* = 16.000, *p* = 0.558 (Control vs hGFAP-ChR2) |
| Supplementary fig. 7e | Two-tailed Mann-Whitney *U* -test | 5 mice in Control  8 in hGFAP-ChR2 | *U* = 15.500, *p* = 0.373 (Control vs hGFAP-ChR2) |
| Supplementary fig. 7f | Two-tailed Mann-Whitney *U* -test | 5 mice in Control  8 in hGFAP-ChR2 | *U* = 19.000, *p* = 0.884 (Control vs hGFAP-ChR2) |
| Supplementary fig. 7g | Two-tailed Mann-Whitney *U* -test | 5 mice in Control  8 in hGFAP-ChR2 | *U* = 18.000, *p* = 0.770 (Control vs hGFAP-ChR2) |
| Supplementary fig. 7i | Two-tailed Mann-Whitney *U* -test | 5 mice in Control  6 in hGFAP-ChR2 | Control vs hGFAP-ChR2  *U* = 9.500, *p =* 0.314 (1 min)  *U* = 11.000, *p =* 0.461 (2 min)  *U* = 0.000, *p =* 0.006 (3 min)  *U* = 0.000, *p =* 0.006 (4 min)  *U* = 0.000, *p =* 0.006 (5 min)  *U* = 0.000, *p =* 0.006 (6 min)  *U* = 0.000, *p* = 0.004 (7 min)  *U* = 0.000, *p* = 0.004 (8 min)  *U* = 0.000, *p* = 0.004 (9 min)  *U* = 2.500, *p* = 0.013 (10 min)  *U* = 0.000, *p* = 0.013 (11 min)  *U* = 0.000, *p* = 0.005 (12 min) |
| Supplementary fig. 7j | Two-tailed Mann-Whitney *U* -test | 5 mice in Control  6 in hGFAP-ChR2 | *U* = 0.000, *p* = 0.006 (Control vs hGFAP-ChR2) |
| Supplementary fig. 7k | Two-way repeated ANOVA and multivariate test (Wilks’ lambda) followed two-tailed Mann-Whitney *U*-test | 6 mice in Control  6 in hGFAP-ChR2 | Wilks lambda = 0.896  F(1, 10) = 1.156  *p* = 0.308  Pre test  *U =* 14.000, *p* = 0.521 (Control vs hGFAP-ChR2)  Test  *U* = 12.000, *p* = 0.337 (Control vs hGFAP-ChR2) |
| Supplementary fig. 7n | Two-way repeated ANOVA and multivariate test (Wilks’ lambda) followed by LSD post hoc analysis | 6 mice in Control  6 in hGFAP-ChR2 | Wilks lambda = 0.973  F(1, 18) = 0.493  *p* = 0.492  Pre test  *U =* 24.000, *p* = 0.949 (Control vs hGFAP-ChR2)  Test  *U =* 19.000, *p* = 0.482 (Control vs hGFAP-ChR2)  Post test  *U =* 11.000, *p* = 0.084 (Control vs hGFAP-ChR2) |
|  |  |  |  |
|  |  |  |  |
|  |  |  |  |
| Supplementary fig. 8b | Wilcoxon signed-rank test | 5 mice in Control  7 mice in hGFAP-ChR2 | Control  *p* = 0.317 (off vs on)  hGFAP-ChR2  *p* = 0.014 (off vs on) |
| Supplementary fig. 8b  Light on | Two-tailed Mann-Whitney *U* -test | 5 mice in Control  7 mice in hGFAP-ChR2 | *U* = 0.000, *p* = 0.003 (Control vs hGFAP-ChR2) |
| Supplementary fig. 8c | Wilcoxon signed-rank test | 5 mice in Control  7 mice in hGFAP-ChR2 | Control  *p* = 0.131 (off vs on)  hGFAP-ChR2  *p* = 0.043 (off vs on) |
| Supplementary fig. 8c  Light on | Two-tailed Mann-Whitney *U* -test | 5 mice in Control  7 mice in hGFAP-ChR2 | *U* = 0.000, *p* = 0.014 (Control vs hGFAP-ChR2) |
| Supplementary fig. 8d | Two-tailed Mann-Whitney *U* -test | 4 mice in Control  5 mice in hGFAP-ChR2 | *U* = 0.000, *p* = 0.014 (Control vs hGFAP-ChR2) |
| Supplementary fig. 8e | Two-tailed Mann-Whitney *U* -test | 4 mice in Control  5 mice in hGFAP-ChR2 | *U* = 6.000, *p* = 0.180 (Control vs hGFAP-ChR2) |
| Supplementary fig. 8f | Two-tailed Mann-Whitney *U* -test | 4 mice in Control  5 mice in hGFAP-ChR2 | *U* = 6.000, *p* = 0.180 (Control vs hGFAP-ChR2) |
| Supplementary fig. 8g | Two-tailed Mann-Whitney *U* -test | 4 mice in Control  5 mice in hGFAP-ChR2 | *U* = 4.000, *p* = 0.081 (Control vs hGFAP-ChR2) |
| Supplementary fig. 9g | One-way repeated ANOVA and post-hoc analysis for multiple comparisons (LSD) | 7 mice | hSyn-mCherry + Vehicle  *p* = 0.601  F(2, 24) = 0.519  *p* = 0.516 (OFF vs 40 Hz)  *p* = 0.735 (OFF vs Constant)  *p* = 0.326 (40 Hz vs Constant) |
| Supplementary fig. 9h | One-way repeated ANOVA and post-hoc analysis for multiple comparisons (LSD) | 7 mice | hSyn-mCherry + Vehicle  *p* = 0.650  F(2, 18) = 0.442  *p* = 0.488 (OFF vs 40 Hz)  *p* = 0.858 (OFF vs Constant)  *p* = 0.366 (40 Hz vs Constant) |
| Supplementary fig. 9i | One-way repeated ANOVA and post-hoc analysis for multiple comparisons (LSD) | 7 mice | hSyn-mCherry + Vehicle  *p* = 0.139  F(2, 18) = 2.206  *p* = 0.478 (OFF vs 40 Hz)  *p* = 0.195 (OFF vs Constant)  *p* = 0.053 (40 Hz vs Constant) |
| Supplementary fig. 9j | One-way repeated ANOVA and post-hoc analysis for multiple comparisons (LSD) | 7 mice | hSyn-mCherry + Vehicle  *p* = 0.075  F(2, 18) = 2.997  *p* = 0.176 (OFF vs 40 Hz)  *p* = 0.025 (OFF vs Constant)  *p* = 0.317 (40 Hz vs Constant) |
| Supplementary fig. 10c | Two-tailed Mann-Whitney *U* -test | 4 mice per group | *U* = 0.000, *p* = 0.021 (PBS vs L-AAA) |
| Supplementary fig. 10e | Two-tailed Mann-Whitney *U* -test | 4 mice per group | *U* = 5.000, *p* = 0.386 (PBS vs L-AAA) |
| Supplementary fig. 10h | One-way repeated ANOVA and post-hoc analysis for multiple comparisons (LSD) | 7 mice in hSyn-oChIEF + PBS  7 mice in hSyn-oChIEF + L-AAA | hSyn-oChIEF + PBS  *p* < 0.001  F(2, 18) = 32.211  *p* < 0.001 (OFF vs 40 Hz)  *p* < 0.001 (OFF vs Constant)  *p* = 0.842 (40 Hz vs Constant)  hSyn-oChIEF + L-AAA  *p* = 0.128  F(2, 18) = 2.306  *p* = 0.050 (OFF vs 40 Hz)  *p* = 0.170 (OFF vs Constant)  *p* = 0.509 (40 Hz vs Constant) |
| Supplementary fig. 10h  hSyn-oChIEF + PBS vs  hSyn-oChIEF + L-AAA | Student’s t-test | 7 mice in hSyn-oChIEF + PBS  7 mice in hSyn-oChIEF + L-AAA | *p* = n/a (OFF)  *p* = 0.076 (40 Hz)  *p* = 0.043 (Constant) |
| Supplementary fig. 10i | One-way repeated ANOVA and post-hoc analysis for multiple comparisons (LSD) | 7 mice in hSyn-oChIEF + PBS  7 mice in hSyn-oChIEF + L-AAA | hSyn-oChIEF + PBS  *p* < 0.001  F(2, 18) = 23.581  *p* < 0.001 (OFF vs 40 Hz)  *p* < 0.001 (OFF vs Constant)  *p* = 0.769 (40 Hz vs Constant)  hSyn-oChIEF + L-AAA  *p* =0.058  F(2, 18) = 3.352  *p* = 0.044 (OFF vs 40 Hz)  *p* = 0.033 (OFF vs Constant)  *p* = 0.878 (40 Hz vs Constant) |
| Supplementary fig. 10i  hSyn-oChIEF + PBS vs  hSyn-oChIEF + L-AAA | Student’s t-test | 7 mice in hSyn-oChIEF + PBS  7 mice in hSyn-oChIEF + L-AAA | *p* = 0.233 (OFF)  *p* = 0.044 (40 Hz)  *p* = 0.007 (Constant) |
| Supplementary fig. 10j | One-way repeated ANOVA and post-hoc analysis for multiple comparisons (LSD) | 7 mice in hSyn-oChIEF + PBS  7 mice in hSyn-oChIEF + L-AAA | hSyn-oChIEF + PBS  *p* = 0.935  F(2, 18) = 0.068  *p* = 0.879 (OFF vs 40 Hz)  *p* = 0.834 (OFF vs Constant)  *p* = 0.719 (40 Hz vs Constant)  hSyn-oChIEF + L-AAA  *p* = 0.085  F(2, 18) = 2.842  *p* = 0.045 (OFF vs 40 Hz)  *p* = 0.066 (OFF vs Constant)  *p* = 0.841 (40 Hz vs Constant) |
| Supplementary fig. 10j  hSyn-oChIEF + PBS vs  hSyn-oChIEF + L-AAA | Student’s t-test | 7 mice in hSyn-oChIEF + PBS  7 mice in hSyn-oChIEF + L-AAA | *p* = 0.064 (OFF)  *p* = 0.011 (40 Hz)  *p* = 0.037 (Constant) |
| Supplementary fig. 10k | One-way repeated ANOVA and post-hoc analysis for multiple comparisons (LSD) | 7 mice in hSyn-oChIEF + PBS  7 mice in hSyn-oChIEF + L-AAA | hSyn-oChIEF + PBS  *p* < 0.001  F(2, 18) = 14.964  *p* < 0.001 (OFF vs 40 Hz)  *p* < 0.001 (OFF vs Constant)  *p* = 0.546 (40 Hz vs Constant)  hSyn-oChIEF + L-AAA  *p* = 0.958  F(2, 18) = 0.043  *p* = 0.879 (OFF vs 40 Hz)  *p* = 0.772 (OFF vs Constant)  *p* = 0.891 (40 Hz vs Constant) |
| Supplementary fig. 10k  hSyn-oChIEF + PBS vs  hSyn-oChIEF + L-AAA | Student’s t-test | 7 mice in hSyn-oChIEF + PBS  7 mice in hSyn-oChIEF + L-AAA | *p* = 0.679 (OFF)  *p* = 0.095 (40 Hz)  *p* = 0.003 (Constant) |
| Supplementary fig. 11a | Two-tailed paired t-test | 5 mice | *p* = 0.009 (Air vs CO_2_) |
| Supplementary fig. 11b | Wilcoxon signed-rank test | 5 mice | *p* = 0.043 (Air vs CO_2_) |
| Supplementary fig. 11c | Wilcoxon signed-rank test | 5 mice | *p* = 0.043 (Air vs CO_2_) |
| Supplementary fig. 11d | One-way ANOVA and post-hoc analysis Dunnett’s t-test to compare all other groups against CO_2_ | 5 mice in CO_2_  5 mice in Base  4 mice in Fox odor  5 mice in Risk assess  5 mice in RC car attack  5 mice in Hand attack | *p* < 0.001  F(5, 23) = 10.625  *p* < 0.001 (CO_2_ vs Base)  *p* = 0.004 (CO_2_vs Fox odor)  *p* < 0.001 (CO_2_ vs Risk assess)  *p* < 0.001 (CO_2_ vs RC car attack)  *p* = 0.030 (CO_2_vs Hand attack) |
| Supplementary fig. 12b | One-way repeated ANOVA and post-hoc analysis for multiple comparisons (LSD) | 8 mice in Control  9 mice in hGFAP-ChR2 | Control  F(2, 21) = 0.131  *p* = 0.518  *p* = 0.750 (Light off 1 vs Light off 2)  *p* = 0.618 (Light off 1 vs Light on)  *p* = 0.857 (Light off 2 vs Light on)  hGFAP-ChR2  F(2, 24) = 3.659  *p* = 0.041  *p* = 0.366 (Light off 1 vs Light off 2)  *p* = 0.014 (Light off 1 vs Light on)  *p* = 0.094 (Light off 2 vs Light on) |
| Supplementary fig. 12b  Light on | Two-tailed Mann-Whitney *U* -test | 8 mice in Control  9 mice in hGFAP-ChR2 | *U* = 9.000, *p* = 0.009 (Light on, Control vs hGFAP-ChR2) |
| Supplementary fig. 13b | Wilcoxon signed-rank test | 6 mice | *p* = 0.028 (Light off vs Light on) |
| Supplementary fig. 13c | Wilcoxon signed-rank test | 5 mice | *p* = 0.686 (Light off vs Light on) |
| Supplementary fig. 13d | Wilcoxon signed-rank test | 5 mice | *p* = 0.257 (Light off vs Light on) |
| Supplementary fig. 13e | Wilcoxon signed-rank test | 4 mice per group | *p* = 0.144 (Pre-fox odor vs Fox odor) |
| Supplementary fig. 13f | Wilcoxon signed-rank test | 4 mice per group | *p* = 0.068 (Pre-fox odor vs Fox odor) |
| Supplementary fig. 13h | Wilcoxon signed-rank test | 10 mice in hGFAP-ChR2 + mCherry  12 mice in hGFAP-ChR2 + Opto-vTrap | hGFAP-ChR2 + mCherry  *p* = 0.005 (Light off vs Light on)  hGFAP-ChR2 + Opto-vTrap  *p* = 0.051 (Light off vs Light on) |
| Supplementary fig. 13i | Two-tailed Mann-Whitney *U* -test | 5 mice per group | *U* = 11.500 *p* = 0.841 (mCherry vs Opto-vTrap) |
| Supplementary fig. 13j | Two-tailed Mann-Whitney *U* -test | 5 mice per group | *U* = 9.500 *p* = 0.548 (mCherry vs Opto-vTrap) |
| Supplementary fig. 13k | Two-tailed Mann-Whitney *U* -test | 5 mice in mCherry  8 mice in Opto-vTrap | *U* = 17.500, *p* = 0.713 (1 min)  *U* = 20.000, *p* = 1.000 (2 min)  *U* = 14.000, *p* = 0.380 (3 min)  *U* = 19.000, *p* = 0.884 (4 min)  *U* = 16.500, *p* = 0.608 (5 min) |
| Supplementary fig. 13l | Two-tailed Mann-Whitney *U* -test | 5 mice in mCherry  8 mice in Opto-vTrap | *U* = 14.000, *p* = 0.380 (1 min)  *U* = 20.000, *p* = 1.000 (2 min)  *U* = 17.500, *p* = 0.714 (3 min)  *U* = 15.000, *p* = 0.464 (4 min)  *U* = 15.000, *p* = 0.464 (5 min) |
| Supplementary fig. 13m | Two-tailed Mann-Whitney *U* -test | 5 mice in mCherry  8 mice in Opto-vTrap | *U* = 13.000, *p* = 0.306 (1 min)  *U* = 20.000, *p* = 1.000 (2 min)  *U* = 17.000, *p* = 0.661 (3 min)  *U* = 15.000, *p* = 0.464 (4 min)  *U* = 17.000, *p* = 0.661 (5 min) |
| Supplementary fig. 13n | Two-tailed Mann-Whitney *U* -test | 5 mice in mCherry  8 mice in Opto-vTrap | *U* = 15.500, *p* = 0.497 (1 min)  *U* = 11.500, *p* = 0.191 (2 min)  *U* = 16.000, *p* = 0.538 (3 min)  *U* = 19.000, *p* = 0.882 (4 min)  *U* = 14.500, *p* = 0.417 (5 min) |
| Supplementary fig. 14b | Two-tailed Mann-Whitney *U* -test | 8 in PSD95  6 in Gephyrin | *U* = 17.500, *p* = 0.0013 (PSD95 vs Gephyrin) |
